# Ingestive behaviors in bearded capuchins (*Sapajus libidinosus*)

**DOI:** 10.1038/s41598-020-77797-2

**Published:** 2020-11-30

**Authors:** Myra F. Laird, Barth W. Wright, Annie O. Rivera, Mariana Dutra Fogaça, Adam van Casteren, Dorothy M. Fragaszy, Patricia Izar, Elisabetta Visalberghi, Robert S. Scott, David S. Strait, Callum F. Ross, Kristin A. Wright

**Affiliations:** 1 Department of Integrative Anatomical Sciences, University of Southern California, Bishop Hall 401, 1333 San Pablo Street, Los Angeles, CA 90033 USA; 2 Department of Anatomy, Kansas City University of Medicine and Biosciences, 1750 Independence Ave., Kansas City, MO 64106 USA; 3 Department of Anthropology, Northwestern University, 1810 Hinman Avenue, Evanston, IL 60208 USA; 4 Department of Biomedical Sciences, Institute of Population Genetics, University of Veterinary Medicine, Veterinärplatz 1, 1210 Vienna, Austria; 5 Neotropical Primates Research Group-NeoPReGo, Manoel Oliveira Bueno, 469, São Paulo, 03643-010 Brazil; 6 Department of Anthropology, Washington University in St. Louis, Campus Box 1114 One Brookings Drive, St. Louis, MO 63130 USA; 7 Department of Psychology, University of Georgia, 125 Baldwin Street, Athens, GA 30602 USA; 8 Department of Experimental Psychology, University of São Paulo, Av. Mello Moraes, 1721, São Paulo, 05508-030 Brazil; 9 Institute of Cognitive Sciences and Technologies, National Research Council (CNR), Via U. Aldrovandi 16b, 00197 Rome, Italy; 10 Department of Anthropology, Rutgers, The State University of New Jersey, 131 George Street, New Brunswick, NJ 08901 USA; 11 Palaeo-Research Institute, University of Johannesburg, Cnr Kingsway and University Road Auckland Park, PO Box 524, Auckland Park, 2006 South Africa; 12 Department of Organismal Biology and Anatomy, University of Chicago, 1027 E 57th St., Chicago, IL 60637 USA; 13 Department of Biomedical Sciences, University of Missouri Kansas City School of Medicine, 2411 Holmes Street, Kansas City, MO 64108 USA

**Keywords:** Biological anthropology, Behavioural ecology

## Abstract

The biomechanical and adaptive significance of variation in craniodental and mandibular morphology in fossil hominins is not always clear, at least in part because of a poor understanding of how different feeding behaviors impact feeding system design (form–function relationships). While laboratory studies suggest that ingestive behaviors produce variable loading, stress, and strain regimes in the cranium and mandible, understanding the relative importance of these behaviors for feeding system design requires data on their use in wild populations. Here we assess the frequencies and durations of manual, ingestive, and masticatory behaviors from more than 1400 observations of feeding behaviors video-recorded in a wild population of bearded capuchins (*Sapajus libidinosus*) at Fazenda Boa Vista in Piauí, Brazil. Our results suggest that ingestive behaviors in wild *Sapajus libidinosus* were used for a range of food material properties and typically performed using the anterior dentition. Coupled with previous laboratory work indicating that ingestive behaviors are associated with higher mandibular strain magnitudes than mastication, these results suggest that ingestive behaviors may play an important role in craniodental and mandibular design in capuchins and may be reflected in robust adaptations in fossil hominins.

## Introduction

**Figure 1:**
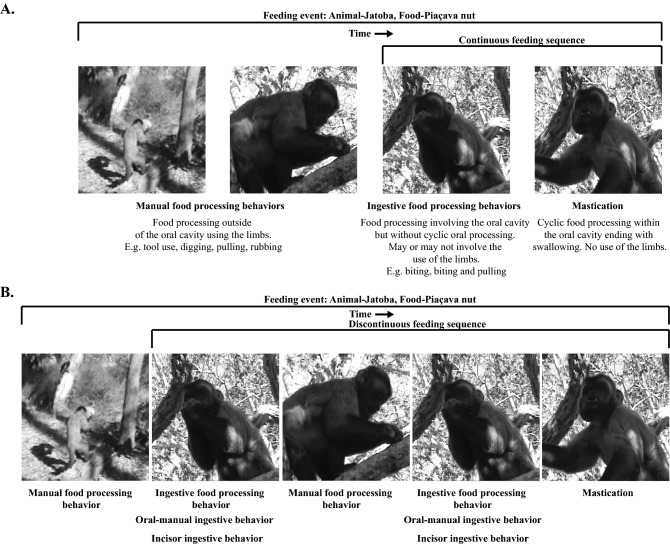
(**A**) Sample feeding events for ‘piaçava’ nuts (*Obrignya* sp.) captured from a video. Manual food processing behaviors take place outside of the oral cavity and involve using the limbs and, in some cases, tools in various combinations. In contrast, ingestive behaviors are defined as food passing into the oral cavity and can exclusively include the oral cavity or a combination of the limbs and oral cavity. Ingestion is typically followed by mastication, which takes place on the postcanine teeth and is characterized by cyclic jaw movements, presumably associated with upward, medial, and anterior movements of the lower teeth relative to the upper teeth during the slow close phase of the gape cycle^68^. A chewing sequence typically begins when the food enters the oral cavity (ingestion) and continues until the food is swallowed or discarded. (**B**) However, chewing sequences may be discontinuous. For example, primates may ingest a food item then later remove it from the oral cavity, then engage in manually processing before ingesting it for a second time. Importantly, not all ingested materials undergo manual processing before ingestion and not all ingested materials are masticated and swallowed. Some food items may be ingested and subsequently discarded. Feeding sequences and feeding events end when the food item is swallowed or discarded.

The relationships between feeding behavior and craniodental and mandibular morphology have been well studied in primates and fossil hominins, but mismatches of craniodental and mandibular morphology with behavior and biomechanical expectations are frequently highlighted^1–6^. For example, members of *Cercocebus* do not differ from members of *Lophocebus* in mandibular robusticity, despite the former frequently consuming mechanically challenging seeds requiring high forces for fracture^5^. One possible explanation for these mismatches is that, with notable exceptions^7–13^, the biomechanical context of ingestive behaviors—how food is brought into the oral cavity (Fig. 1; Supplementary Information Table 1)—is not well understood.

**Table 1:** Fazenda Boa Vista individuals and their social group, sex, and age classifications. Age classifications were at the time of data collection in June 2015 and are based on records kept by field site managers. Social group abbreviations: *CH *Chicao Group, *MN *Mansinho Group.

Name	Social group	Sex	Age (year of birth, if known)	Percentage of data
Catu	MN	Male	Adult (2007)	0.03
Cenoura	CH	Female	Subadult (2013)	0.07
ChuChu	CH	Female	Adult (unknown)	0.03
Coco	MN	Male	Subadult (2009)	0.12
Dita	CH	Female	Adult (unknown)	0.03
Divina	CH	Female	Subadult (2012)	0.03
Donzela	CH	Female	Subadult (2013)	0.03
Doree	CH	Female	Adult (2007)	0.04
Jatobá	CH	Male	Adult (unknown)	0.07
Pacoca	CH	Female	Adult (2009)	0.07
Pamonha	CH	Female	Adult (2009)	0.05
Patricia	CH	Female	Subadult (2013)	0.07
Piaçava	CH	Female	Adult (unknown)	0.01
Presente	CH	Male	Subadult (2011)	0.21
Tais	MN	Female	Subadult (2011)	0.10
Teninha	MN	Female	Adult (unknown)	0.02

Craniomandibular loading regimes (external forces on the skull), stress regimes, and strain regimes (patterns of internal forces or strains)^14^ are associated with a variety of feeding behaviors involving the jaws and tongue^1,5,15–19^. The use of limbs and tools to process food items prior to ingestion may also alter craniomandibular loading during subsequent ingestion and mastication (Fig. 1; Supplementary Table S1)^20,21^. Laboratory studies of macaques and capuchins have found that ingestive biting on the incisors, premolars, and molars produces mandibular bone strain magnitudes equal to or higher than strains recorded during mastication, suggesting these behaviors might be important in feeding system design^7,8,13^. These laboratory studies have been complemented by finite element analyses (a modeling technique used to simulate how structures of complex geometry respond to loading) showing that ingestive biting on the premolars and incisors elicits strains in the rostrum that are higher and different in mode from those associated with molar bites, e.g., Refs.^4,22^.

In combination, these laboratory and simulation studies suggest that both ingestive and masticatory behaviors may be important components of feeding system design (form–function relationships), so it is necessary to understand the biomechanical context of a full range of feeding behaviors. The frequencies and durations of different feeding behaviors are likely determinants of overall, summed loading regimes acting on primate skeletons^5,7,13,23,24^. For example, a single bite may produce higher strain magnitudes, but frequent mastication for longer periods of time may result in greater summed loading, necessitating design features to resist fatigue of bones, joints, and muscles. Thus, integration of laboratory and field data is necessary to assess the frequencies and durations (of time) of loading and stresses and strains on the feeding system and their relationships to craniodental and mandibular adaptations in non-human primates and fossil hominins^25^.

One particularly important determinant of the biomechanical impact of feeding behaviors on feeding system design is the location of biting along the toothrow. Location impacts craniodental and mandibular loading regimes through changes in orientation and magnitude of bite force, through changes in bending and/or torsional moments, and through changes in patterns of muscle activity, all of which can affect stress and strain regimes throughout the facial skeleton^26–28^. In humans, for example, the greatest amount of bite force is produced at the first molar and decreases anteriorly and posteriorly along the toothrow^27^. Summed loading on the feeding system presumably reflects differences in location along the toothrow, but field data are necessary to precisely describe the locations of food items within the mouth during natural feeding sequences.

Here we assess the relationships between food material properties (FMPs), and the frequencies (rate of behavioral occurrence across the entire recording time), and durations (time, in seconds [s]) associated with different ingestive behaviors in a population of wild bearded capuchins (*Sapajus libidinosus*, Cebidae). With an eye to the biomechanical impact of these behaviors, data were collected during the dry season, when the capuchins at Fazenda Boa Vista consume food tissues with highest annual stiffness and toughness^29^.

Robust capuchins (a group that includes bearded capuchins) have been the focus of numerous morphological comparisons, in vivo physiological and biomechanical modeling studies, and are commonly used as an extant model of hard object feeding for comparison to *Australopithecus*^13,28,30–32^. Importantly, robust capuchins are associated with craniofacial adaptations for producing high bite forces, particularly on the anterior dentition. These features include thick enamel, large and anteriorly positioned masticatory muscles, a large mandibular corpus and symphysis, and a broad face^16,28,31,33,34^. A laboratory study on soft- and hard-shell seeds suggests robust capuchins favor the use of their anterior teeth during ingestion^35^. Bearded capuchins also engage in a range of ingestive behaviors, including digging, biting, and tool use, which typically involves cracking nuts (particularly ‘piaçava’ nuts [*Orbignya* sp.]^36^) using a stone hammer (Fig. 1). Integrating laboratory and fieldwork data on robust capuchin ingestive behaviors can provide a comparative framework for future analyses to determine biomechanical patterns associated with hard object feeding in non-human primates and fossil hominins.

Food material properties are commonly used as proxies for force generation in the feeding system, with the assumption that the processing of mechanically challenging foods induces higher stresses and strains that might necessitate morphological adaptations in response, e.g., Refs.^37,38^ Primate laboratory and field studies have focused on two FMPs—toughness and elastic modulus. Toughness is defined as the work needed to initiate and propagate a crack through the food item^39,40^, and elastic modulus is stiffness, defined as the ratio of stress to strain in the elastic region of a given food tissue, e.g., Refs.^39,40^ We address three hypotheses regarding relationships between variation in FMPs and variation in the types, frequencies, and durations of ingestive behaviors.

### Hypotheses

Our null hypotheses are that variation in FMPs is not associated with variation in feeding behavior, and that all feeding behaviors are used in equal proportion, regardless of FMPs. We test three alternative hypotheses suggesting relationships between variation in FMPs and variation in types, frequencies and durations of different feeding behaviors.

### Hypothesis 1: Variation in food material properties is related to frequencies, durations and relative proportions of different behaviors—ingestive behaviors, manual behaviors and mastication—across the feeding sequence

Ingestive (including oral and oral-manual) behaviors are predicted to be associated with higher toughness and elastic modulus values than solely manual behaviors, across all foods. In association with mechanically challenging foods, we also predict (oral and oral-manual) ingestive behaviors to occur at higher frequencies and take longer durations of time compared to manual behaviors or mastication, reflecting the difficulty in accessing food items with these properties.

Importantly, some food parts (tissues) are typically associated with one type of behavior, e.g., bearded capuchins (*S. libidinosus*) typically process the exocarp of ‘piaçava’ nuts using stone tools^36^. We hypothesize that feeding on food parts with higher toughness and elastic modulus values will be associated with higher frequencies of oral and oral-manual ingestive behaviors, and longer durations of time spent on these ingestive behaviors compared to manual behaviors *within* each food. While we were unable to test for FMP differences between ingestive behaviors and mastication, we predict that *S. libidinosus* uses higher frequencies of ingestive behaviors and spends longer durations of time on ingestive behaviors compared to mastication *within* foods (between tissues).

### Hypothesis 2: Variation in FMPs is related to variation in frequency and duration among oral and oral-manual ingestive behaviors

Oral ingestive behaviors involve the oral cavity only, and not the limbs; whereas, oral-manual ingestive behaviors involve the oral cavity and the limbs. We predict that, across foods, feeding on foods with higher toughness and stiffness will be associated with increased frequency and longer durations of oral-manual behaviors compared with oral-only ingestive behaviors, reflecting differences in force capacity of the feeding system compared to the feeding system and limbs.

### Hypothesis 3: Variation in FMPs impacts the frequency and duration of the location along the toothrow for ingestive behaviors

Robust capuchins have adaptations for producing high bite forces on the anterior dentition, but Wright (2005) suggests the anterior dentition is infrequently used. We predict that the anterior toothrow—mesial to the premolars and molars—will be associated with higher FMP values, higher frequencies, and longer durations of (oral and oral-manual) ingestive behaviors.

## Results

### Hypothesis 1: Food material properties and manual behaviors, ingestive behaviors, and mastication

**Figure 2:**
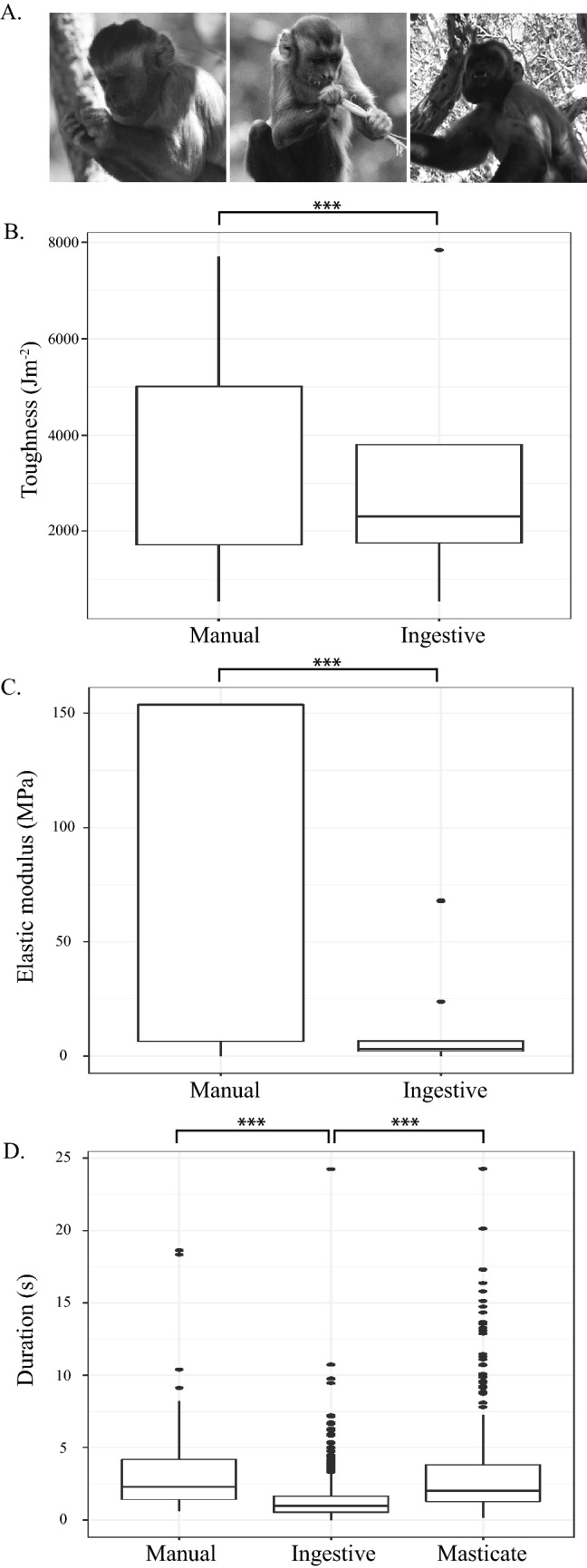
(**A**) Photos of manual, oral-manual ingestive, and masticatory behaviors in *Sapajus libidinosus* taken at Fazenda Boa Vista by Mariana Dutra Fogaça. (**B**) Boxplot of variation in food toughness between manual and ingestive behaviors. (**C**) Boxplot of variation in food elastic modulus between manual and ingestive behaviors, and (**D**) boxplot of variation in the duration of manual, ingestive, and masticatory behaviors. For all plots, the upper and lower bound of the boxes corresponds with the 25th and 75th percentiles and the whiskers extend 1.5 times the interquartile range in either direction. The median is represented by a horizontal line inside the boxes. A significance level, p < 0.01, is indicated by three asterisks. Figure generated in R (2017; https://www.R-project.org).

Across all foods, there were significant differences in FMPs between manual behaviors and ingestive behaviors: manual behaviors were associated with foods with significantly higher toughness (*p* < 0.01; Fig. 2) and stiffness values (*p* < 0.01; Fig. 2) than were ingestive behaviors. Across all foods, ingestive behaviors were the most frequent, followed by mastication; manual behaviors were the least common during the recording period. Ingestive behaviors accounted for 70.0%, mastication for 24.7%, and manual behaviors for 3.7% of all recorded behavioral sequences. Across all foods pooled there were significant differences in durations of these three behavioral categories (Fig. 2). Pairwise comparisons reveal ingestive behaviors were significantly shorter in duration than either manual behaviors or mastication (both *p* < 0.01) but there was no difference in duration between mastication and manual behaviors (*p* = 0.80). Overall our wild bearded capuchin subjects spent similar amounts of time in manual preparation and mastication of their food, and less time in ingestion, but ingestive behaviors were more frequent.

Within individual foods (between food tissues), there was no difference in toughness or elastic modulus values associated with manual and ingestive behaviors. There were no differences in behavioral durations between manual, ingestive, and masticatory behaviors within ‘bananinha’ (the term used for a small yellow fruit in the Fabaceae family), pods (Fabaceae family), bromeliads (Bromeliaceae family), and berries (unknown family). However, there were significant difference in durations of behaviors used in consuming fruits (multiple families, Table 3), ‘piaçava’ (*Obrignya* sp.), insects (Formicidae family), sugar cane (*Saccharum* sp.), tucum (*Astrocaryum campestre*), and underground storage organs (USOs; Poaceae family). For all of these foods, behavioral durations during ingestion were significantly shorter than either mastication (fruit, ‘piaçava’, sugar cane, tucum, and USO *p* < 0.01; insects *p* = 0.03) or manual behaviors (fruit and ‘piaçava’ *p* < 0.01).

### Hypothesis 2: Oral and oral-manual ingestive behaviors

**Figure 3:**
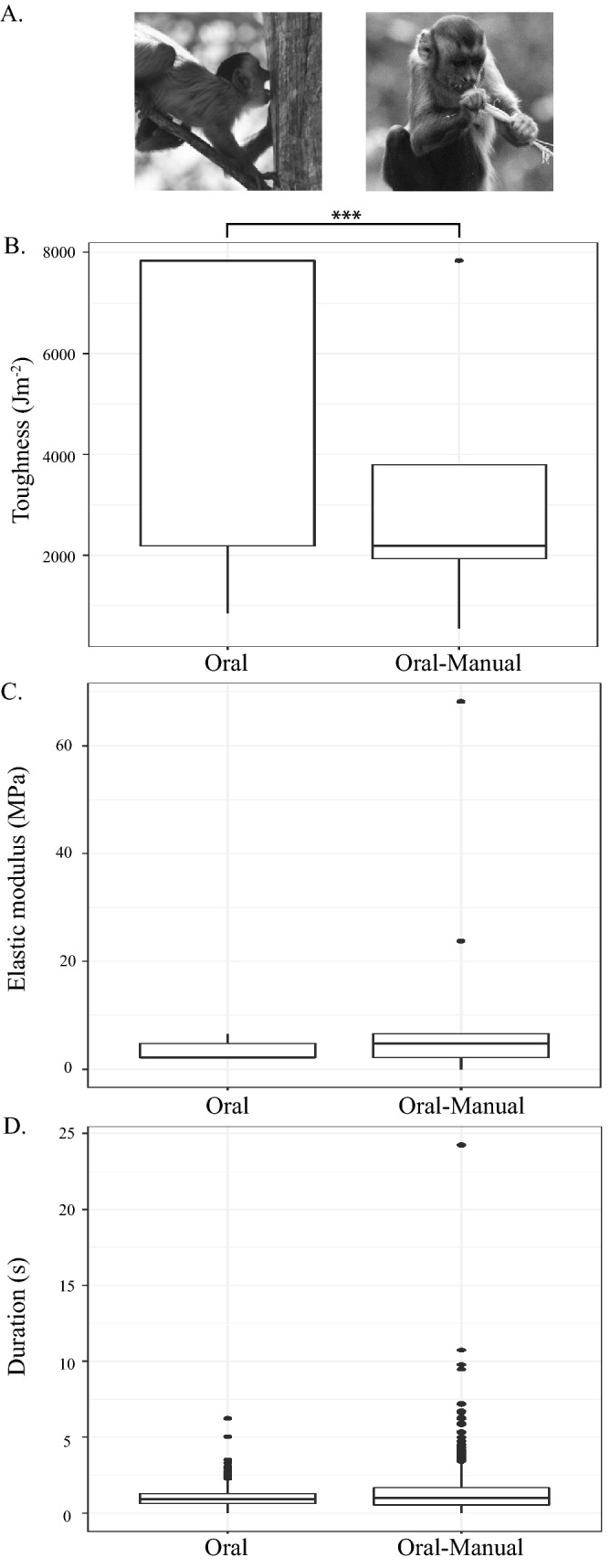
(**A**) Photos of oral and oral-manual ingestive behaviors in *Sapajus libidinosus* taken at Fazenda Boa Vista by Mariana Dutra Fogaça. (**B**) Boxplot of variation in food toughness between oral and oral-manual ingestive behaviors. (**C**) Boxplot of variation in food elastic modulus between oral and oral-manual ingestive behaviors, and (**D**) boxplot of variation in the duration of oral and oral-manual ingestive behaviors. The upper and lower bound of the boxes corresponds with the 25th and 75th percentiles and the whiskers extend 1.5 times the interquartile range in either direction. The median is represented by a horizontal line inside the boxes. The sample size of toughness and elastic modulus measures for oral-manual behaviors is small and the median line corresponding with lower bound of the box. A significance level, p < 0.01, is indicated by three asterisks. Figure generated in R (2017; https://www.R-project.org).

Ingestive behaviors were subdivided into oral or oral-manual behaviors. Oral behaviors composed 13.0% and oral-manual behaviors 87.0% of recorded ingestive behaviors, but oral and oral-manual ingestive behaviors did not vary in overall duration (*p* = 0.11; Fig. 3). Across all foods, oral ingestive behaviors were associated with foods of higher toughness compared to oral-manual behaviors (*p* < 0.01; Fig. 3); there were no differences in elastic modulus values between oral and oral-manual ingestive behaviors.

### Hypothesis 3: Toothrow positioning

**Figure 4:**
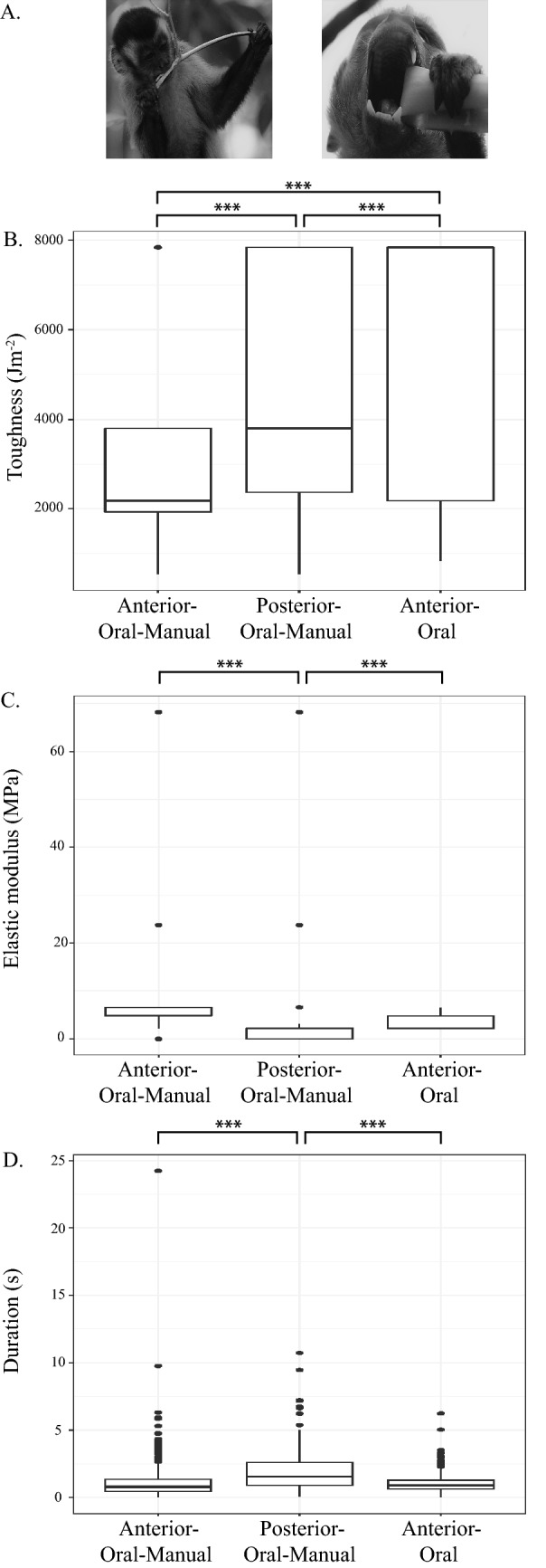
(**A**) Photos of oral and oral-manual ingestive behaviors at the anterior and posterior dentition in *Sapajus libidinosus* taken at Fazenda Boa Vista by Mariana Dutra Fogaça. (**B**) Boxplot of variation in food toughness between oral and oral-manual ingestive behaviors on the anterior and posterior dentition. (**C**) Boxplot of variation in food elastic modulus between oral and oral-manual ingestive behaviors on the anterior and posterior dentition, and (**D**) boxplot of variation in the duration of oral and oral-manual ingestive behaviors on the anterior and posterior dentition. The upper and lower bound of the boxes corresponds with the 25th and 75th percentiles and the whiskers extend 1.5 times the interquartile range in either direction. The median is represented by a horizontal line inside the boxes. The sample size of toughness and elastic modulus measures for each behavior location is small, and the median line corresponds with upper or lower bound of the box. A significance level, p < 0.01, is indicated by three asterisks. Figure generated in R (2017; https://www.R-project.org).

Ingestive feeding behaviors (oral and oral-manual behaviors) were grouped by position along the tooth row; i.e., the anterior (canine and incisor) or posterior (postcanine) dentition. Thirteen percent of oral behaviors occurred on the anterior dentition. The remaining 87.0% of ingestive behaviors were oral manual, and 24.5% occurred on the posterior dentition and 62.5% on the anterior dentition. The total duration of feeding time during ingestive behaviors differed significantly based on tooth row position (anterior and posterior dentition; *p* = < 0.01; Fig. 4). *Sapajus libidinosus* spent greater amounts of time ingesting food items on the postcanine dentition than on the anterior dentition (both *p* < 0.01).

There were significant differences in toughness and elastic modulus values between ingestive behaviors on the anterior and posterior dentition (*p* < 0.01; Fig. 4). Food processed using oral and oral-manual behaviors on the anterior dentition had significantly higher elastic modulus values than foods processed using oral-manual behaviors on the postcanine dentition (all *p* < 0.01). Anterior oral-manual ingestive behaviors were used on foods with significantly higher toughness values compared to posterior oral-manual behaviors, and posterior oral-manual behaviors were used on significantly tougher foods than were anterior oral behaviors (both *p* < 0.01).

The total duration of feeding time during ingestive behaviors differed significantly based on tooth row position (anterior and posterior dentition; *p* = < 0.01; Fig. 4). *Sapajus libidinosus* spent greater amounts of time ingesting food items on the postcanine dentition than on the anterior dentition (both *p* < 0.01). Oral-manual ingestive behaviors composed 24.50% of ingestive behaviors, oral-manual behaviors on the anterior dentition was 62.50% of ingestive behaviors, and oral anterior behaviors were 13.00% of ingestive behaviors.

## Discussion

It is generally agreed that progress in our understanding of form-function relationships in the feeding systems requires better information on the impact of food material properties on feeding behavior, and hence loading regimes, in the feeding systems of wild primates^25^. Data for this study of feeding behavior in bearded capuchins were collected over the span of one month during the dry season. While the short time period covered by this study does not capture annual—or supra-annual, climatic—variation in feeding behavior, the dry season is the time when annual maximum food stiffness and toughness values are the highest^29^. Hence, the results of this study are relevant to hypotheses linking feeding system morphology to consumption of particularly mechanically challenging food items during times of scarcity—“fallback foods”^41^.

Variation in FMPs of the foods eaten by wild robust capuchins noted in the present study (and Refs.^28,29^) was far greater than that of FMPs tested in experimental settings. For example, the toughest and stiffest food used in laboratory tests to date has been popcorn seeds, with toughness and stiffness values of 2979 ± 678.3 J m^−2^ and 325 ± 218.8 MPa^13^, whereas the highest average toughness values from our field data were 7838.1 J m^−2^ for sugar cane, and the highest elastic modulus measures were 16,000.6 MPa from ‘piaçava’ endocarp. (Of note, our elastic modulus values for ‘piaçava’ endocarp are minimum values and we recorded, but do not report here, several other higher values that we consider potentially anomalous.) This suggests that wild *S. libidinosus* foods are orders of magnitude tougher and stiffer than the foods used to measure strain in previous laboratory experiments, e.g. Ref.^13^ Although previous experimental studies suggest that FMPs have a minimal influence on strain magnitudes at lower stiffness and toughness values^13^, variation in FMPs may still be related to a greater proportion of variation in strain magnitude for these highly mechanically challenging food items.

Our first hypothesis posited that in wild *Sapajus libidinosus* the most mechanically challenging parts of food items would be preferentially processed using oral and oral-manual ingestive behaviors, rather than manual behaviors. In fact, in this study oral and oral-manual ingestive behaviors were associated with *lower* food toughness and elastic modulus values than manual behaviors. Assuming that all food items are eventually processed intra-orally, these results suggest that bearded capuchins process their toughest and stiffest food items manually before processing them orally.

The summed effect of loading of the feeding system is a function of both durations and frequencies of different feeding behaviors. During the recording period of this study bearded capuchins engaged in manual behaviors (and mastication) for longer overall durations than ingestive behaviors, but ingestive behaviors were used far more frequently. Laboratory studies show that in capuchins, as in macaques and rabbits, the mandible experiences higher strain magnitudes during ingestion than during mastication^7,13^. The combination of a high frequency of ingestive behaviors (measured in this field study) and high strain magnitudes during ingestion (measured in previous laboratory work using strain gauges in *Sapajus apella*^13^) suggests ingestive behavior may be an important source of loading, stress and strain in the cranium and mandible and, by extension, that capuchin craniomandibular morphology might include adaptations for generating and resisting forces required for ingestion-related feeding behaviors.

Our results also indicate that while feeding behavior is affected by FMPs, feeding behavior is also probably food-specific. While tissue-specific FMPs were not available for all foods, there were no differences in FMP values for manual and ingestive behaviors within foods (between food tissues). This suggests that factors outside of FMPs play a significant role in food processing across foods and within foods. For example, a primate’s feeding behavior is also likely to be influenced by food geometry (size and shape) and other properties, such as texture, e.g., Ref.^42^ Food size at ingestion influences gape and available bite forces at a given gape^43^, and likely has an impact on the location of ingestion (along the toothrow) and choice of behavior. Food size is argued to impact gape during ingestion in strepsirrhines and catarrhines and may influence ingestive behaviors^44,45^. Factors such as food size, in addition to FMPs, are important considerations for future studies on ingestive behaviors.

Our second hypothesis predicted that oral-manual behaviors would be associated with higher values of stiffness and toughness, longer durations, and higher frequencies relative to oral ingestive behaviors. Oral-manual ingestive behaviors imply that some craniodental loading regimes include forces arising outside of the facial and masticatory musculature. For example, when processing sugar cane, robust capuchins used their forelimbs or hind limbs to pull/strip away parts of the sugar cane sheath and/or torque items while holding the sugar cane in their mouths and pulling in the opposite direction with their nuchal region and head. Our results revealed no differences in the durations of oral versus oral-manual behaviors, nor in the elastic moduli of the foods eaten using these behaviors, but compared to oral ingestive behaviors, oral-manual behaviors were associated with foods of *lower* toughness.

In the laboratory, use of the hands during feeding seems to be associated with a wider range of mandibular strain magnitudes and orientations than when the hands were restricted^13^, possibly because of addition of forces from outside of the feeding system. The fact that hand use during ingestion is associated with foods of lower toughness in our study suggests that these behaviors may be associated with more variable strain magnitudes and orientations in wild capuchins as well. Lower toughness may be associated with a more variable strain environment because there may simply be fewer mechanical solutions to the problem of fracturing a very tough food.

Our third hypothesis predicted that the anterior dentition would be used with longer durations and higher frequencies during ingestive behaviors on foods with higher values of stiffness and toughness. Field observations of robust capuchins have noted the use of the anterior dentition for mechanically challenging food items^46–48^, but few studies have quantitatively assessed the impact of FMPs on placement of food items along the toothrow in wild robust capuchins (but see Refs.^28,49^). We found that the most mechanically challenging food parts were placed on the anterior dentition during oral ingestive behaviors, which is consistent with data from captive robust capuchins^35^. Foods such as pods (Fabaceae family) and USOs, were most frequently processed on the postcanine dentition, whereas the anterior dentition was commonly used to initiate cracks in foods such as sugar cane and tucum. Postcanine ingestive behaviors occurred for longer durations relative to ingestive behaviors on the anterior dentition, reflecting repeated postcanine biting on tough foods of smaller sizes. Overall, the postcanine teeth were used for longer durations of time, but ingestive behaviors on the anterior dentition were used more frequently during the recording period.

The location and magnitude of biting forces in robust capuchins’ feeding has been a subject of debate. The mandible of robust capuchins is suggested to reflect adaptations for powerful mastication in comparison to powerful ingestion^50^, but differences in the anterior mandibular corpus suggesting increased resistance to torsion could reflect increased resistance to stresses and strains during ingestion or mastication^16^. Indeed, the anterior positioning of the masseter and temporalis muscles in robust capuchins may allow the group to produce relatively high bite forces on the anterior dentition, even though high anterior bite forces are thought to be used relatively infrequently^28,32^. Foods in our study processed on the anterior dentition during oral ingestion were stiffer and tougher than foods orally ingested on the postcanine dentition, supporting the hypothesis that feeding on the anterior dentition may be associated with greater stresses and strains in the teeth and jaws, and hence may be an important influence on mandibular design. However, it is important to note the difference between longer duration of time spent using the postcanine dentition and the higher frequency of behaviors employing the anterior dentition during the recording period. These results imply that differences in mandibular loading along the toothrow during ingestive behaviors are related to differences in FMPs as well as a combination of behavioral duration and frequency.

Ingestive behaviors and their influence on craniomandibular morphology appear to vary by taxon^9^—e.g., marmosets have adaptations for wide gapes used for specialized tree-gouging behaviors^51^—but the extent to which ingestive behaviors influence skull morphology is not completely understood. Strain magnitudes and orientations are thought to differ across ingestive behaviors in robust capuchins^13^, and our results suggest the use and location of ingestive behaviors in *S. libidinosus* are food-specific and influenced by FMPs. Among the few primates in which ingestive behaviors, FMPs, and craniomandibular morphology have been extensively studied are *Cercocebus* and *Lophocebus*^5,6,11,52,53^. In particular, *Cercocebus atys* has been compared to *Australopithecus* as both taxa possess enlarged premolars and thick molar enamel^6^. *Cercocebus* cracks hard seeds on the premolars, whereas *Lophocebus* uses the anterior dentition to process less mechanically challenging materials^5^. However, there is only weak support for morphological differences between *Cercocebus* and *Lophocebus* relating to loading and stress and strain^5^. What are the implications of experimental and behavioral results from robust capuchins for feeding adaptations in fossil hominins?

Robust capuchins, as represented by *S. libidinosus,* exhibit morphological traits that enhance their ability to produce elevated bite forces, e.g., Ref.^28^; our analyses show that a large proportion of their feeding sequences entail ingestive behaviors and that the plant tissues processed during ingestion are often tougher than those processed using manual behaviors. It follows that there may be morphological traits in extant capuchins that are adaptations for resisting the forces generated during ingestion of resistant foods. Generalizing across primates, including early hominins, one cannot discount the possibility that robust traits in the feeding apparatus of some taxa are adaptations for ingestion rather than mastication. Some derived australopith craniodental traits are hypothesized to be adaptations for ingesting mechanically resistant foods^4,22^. Robust capuchin behaviors do not speak directly to this hypothesis, but they provide comparative data that are compatible with it.

Of course, one should not suppose that ingestive behavior in robust capuchins is perfectly analogous with that of australopiths. Robust capuchins and australopiths share flaring zygomatics, as well as relatively large premolars^28, 34,46,48,54^. However, robust capuchins also have morphological adaptations for processing objects on the anterior detention and behaviorally favor placing mechanically challenging foods on the anterior dentition. If ingestion played a role in the evolution of derived craniodental traits in some australopiths, then those behaviors presumably took place on the postcanine teeth or are not reflected in tooth size. In this regard, the ingestion of hard objects on the premolars might have been adaptively significant in australopiths^4,22^. Ingestion is unlikely to explain the evolution of all derived craniodental traits in australopiths, but given that discourse about these traits has traditionally focused on mastication, our findings suggest that ingestive behaviors should be given due consideration as a potential influence on australopith feeding adaptations.

This study also highlights the benefits of integrating laboratory data and fieldwork to understand the relationships between craniodental morphology and behavior. Additional field work detailing ingestive behaviors in other primate species will help address the extent to which FMPs drive behavioral changes and the locations of loading (on the toothrow), and experimental work is needed to examine the role of the postcrania in loading the craniodental system. These data can be used to include ingestive behaviors in finite element analyses (FEA) of the feeding system. To date, studies examining feeding loads, stresses and strains in fossil hominins and primates have exclusively examined simple biting^4,22^. This might reasonably approximate mastication, but our results suggest that ingestion-related loading is more complicated and variable than mastication, and ingestive strain magnitudes are high compared to mastication^7,8,13^. Expanding FEA studies to include a range of ingestive and oral-manual behaviors may improve our understanding of which aspects of morphology might be expected to reflect variation in ingestive behaviors. Integrating field, experimental, and modeling data is critical to addressing questions of primate feeding adaptations^25^.

## Methods

### Data collection

*Sapajus libidinosus* (bearded capuchins) were studied in a population inhabiting the area in and around the site of Fazenda Boa Vista from May to June 2015. This site occupies privately owned land in the Cerrado-Caatinga (open woodland) ecotone in Gilbués, Piauí, Brazil (9° 39′ S, 45° 25′ W)^55,56^. The region has low-nutrient sandy soils and highly seasonal and interannually variable precipitation, 800–1600 mm, with the vast majority of this precipitation coming in November–April^54^. Sandstone ridges, pinnacles, and mesas rising steeply to 20–100 m above the plain punctuate the landscape^56^. The site is approximately 420 m.a.s.l. Previous observations of robust capuchins in South America suggest that their diet varies seasonally. The dry season in the Amazon has been associated with increased consumption of insects (Formicidae family), palm (*Attalea *sp.), and *Astrocaryum* sp*.* seeds; whereas food preference shifted to soft fruits in the wet season^29,54^. As in wet tropical rainforest, the more seasonal forest of Fazenda Boa Vista sees a period of more abundant soft fruit fruiting at the tail end of the wet season^57^, however our period of study was deeper in the dry season and thus after ripe fruit had been lost due to predation or over ripening. Thus, the period of data collection in this study corresponds to a period of fallback food use^41^ when biomechanical performance might reasonably be expected to influence fitness.

Over 1400 observations of feeding events (manual or ingestive feeding behaviors or mastication) were obtained from over 30 h of video recordings of 15 (4 males, 11 females) adult and subadult wild bearded capuchin monkeys (Table 1). Observations were collected from individuals in two separate social groups (Chicao-CH and Mansinho-MN). The CH group consisted of 21 members (2 adult males, 6 adult females, 1 subadult male, 1 subadult female, 5 juveniles, and 6 infants) and the smaller MN group consisting of 7 members (2 adult males, 1 adult female, 1 subadult male, 1 subadult female, 1 juvenile, and 1 infant). Here we included six subadults that were at least two years of age; adults and subadults were analyzed together. A previous study found that *Sapajus libidinosus* subadults and adults at Fazenda Boa Vista process foods of similar FMPs^58^. Males and females were analyzed together due to the low number of males, although FMP sex differences have been noted in other studies^35^. Data collection was approved by Institutional Animal Care and Use Committees at Kansas City University of Medicine and Biosciences protocol 629641-1 and University of Albany protocol 14-009. All data were collected in accordance with the relevant guidelines and regulations.

### Behavioral sampling

Videos of fully habituated wild bearded capuchin feeding behaviors were captured ad libitum using Sony Handycam video cameras (HD CRX405) by six of the authors for a period of approximately 1 month, from May–June 2015. Researchers worked in pairs with one-person filming and the other collecting food items. Daily follows of groups commenced early in the morning with the first animal encountered, and video recording was discontinuous in order to capture just the feeding events^59^. The terrain at Boa Vista is easily navigated and relatively open, and no animals were filmed concurrently. Recording continued until the monkey went out of view for over a minute. Subsequently, the next animal encountered would be filmed until out of view for over a minute. It was sometimes not possible to record complete feeding sequences because individuals frequently moved temporarily (less than a minute) out of view. The most common cause of a recording gap was the monkey turning away from the cameraperson. In continuous recordings, if a behavior was consistent over a temporary recording gap, the behavioral frequencies and durations were interpolated. If the behavior changed over a temporary recording gap, the original behavior (e.g., manual processing) was recorded as ending when the animal went out of view and the new behavior (e.g., mastication) commenced once the animal came into view. This approach likely introduces recording-based bias in the behavior frequencies and durations. However, the temporary gaps were less than a minute, and the overall effect of this bias is likely to be small. For each video segment, the age, sex, and, if known, name of the individual, as well as encountered food items, were recorded in the audio of the video recording. Individual and food identifications were reviewed and updated in the lab (Table 1). The population of bearded capuchins at the Fazenda Boa Vista site have been continuously observed since 2005. Records of births and deaths, and photo records were used to identify the monkeys recorded in videos as to age, sex, and individual.

Each usable video sequence was viewed frame-by-frame by one of the authors (MFL) to record specific behaviors performed by the focal individual, the duration of these behaviors (calculated from the video frame rate, 30 frames per second; Table 2), and food information (Supplementary Tables S2 and S3). Behaviors were first recorded as either manual, ingestive, or mastication. Manual behaviors commenced when the individual used their hands to manipulate the food item and ended when the behavior ended or the food was ingested. Ingestive behaviors were defined as the food item entering the oral cavity and ended with the food exiting the oral cavity or the start of mastication. Mastication was defined as the start to end of rhythmic gape cycles. Ingestive behaviors were subclassified as either oral or oral-manual and the location of the ingestive behavior on the toothrow (anterior or postcanine dentition) was recorded. To test for intraobserver error, four of the videos were coded three times. A one-way analysis of variance was used to test for differences in behavior duration between coding instances, and there were no significant differences (f-value = 0.0549, df = 1, *p* = 0.82).

**Table 2:** Average (and standard deviations) of behavior durations in seconds for each food group.

Common name	Bananinha	Berry	Bromeliad	Cane	Fruit^a^	Insects	Piaçava	Pods	Tucum	USO^b^
Manual behaviors	–	7.74 (7.74)	–	–	1.71 (0.84)	–	4.07 (3.83)	–	2.31 (0.81)	4.31 (3.62)
Mastication	5.48 (3.78)	1.45 (9.8)	1.75 (1.39)	2.84 (1.31)	2.12 (1.61)	1.85 (0.97)	5.3 (4.18)	11.7 (2.98)	3.38 (3.87)	4.1 (4.61)
Ingestive behaviors	1.06 (2.65)	1.83 (6.77)	1.46 (1.08)	1.7 (1.3)	1.03 (0.74)	1.16 (1.19)	1.07 (1.02)	0.58 (0.55)	1.26 (1.37)	1.45 (2.03)
Oral ingestive behaviors	–	–	–	1.26 (0.99)	1.02 (0.38)	0.9 (0.45)	–	0.38 (0.21)	–	0.85 (0.62)
Oral-manual ingestive behaviors	1.06 (2.65)	1.83 (6.77)	1.46 (1.08)	2.05 (1.41)	1.03 (0.74)	1.34 (1.48)	1.07 (1.02)	0.64 (0.62)	1.26 (1.37)	1.47 (2.06)
Incisor oral-manual ingestive behaviors	1.06 (2.65)	2.49 (9.63)	1.81 (1.8)	1.36 (0.99)	1.01 (0.73)	0.8 (0.73)	1.05 (1.09)	0.64 (0.62)	1.14 (1.17)	1.23 (2.07)
Canine oral-manual ingestive behaviors	–	–	–	0.72 (0.02)	1.43 (1.12)	–	1.27 (0.05)	–	–	0.92 (0.07)
Postcanine oral-manual ingestive behaviors	–	1.2 (0.92)	1.32 (0.59)	2.33 (1.45)	1.14 (0.63)	3.22 (1.94)	1.14 (0.36)	–	4.1 (3.58)	2.06 (1.96)
Incisor oral ingestive behaviors	–	–	–	1.26 (0.99)	1.02 (0.38)	0.9 (0.45)	–	0.38 (0.21)	–	0.85 (0.62)
Canine oral ingestive behaviors	–	–	–	–	–	–	–	–	–	–
Postcanine oral ingestive behaviors	–	–	–	–	–	–	–	–	–	–

^a^All fruit taxa and tissue types from Table 3 were combined for duration measures in Table 2.

^b^Underground storage organ (USO).

**Table 3:** Food material property values and associated behaviors. Scientific names follow Santos (2015)^69^.

Family	Scientific name	Tissue type	Common name	Average elastic modulus (SD) (MPa); number of tests	Maximum elastic modulus (MPa)	Average toughness (R) (SD) (J m^−2^); number of tests	Maximum toughness (R) (J m^−2^)	Manual or ingestive behaviors
Leguminosae-Fabaceae	?		Bananinha	0.02 (0.01); n = 5	0.04			Manual
Leguminosae-Fabaceae	?		Bananinha	0.01 (0.01); n = 6	0.03	1500.03 (688.72); n = 3	2091.38	Ingestive
?	?		Berry	0.02 (0.02); n = 12	0.06	2371.39 (2227.02); n = 2	3946.13	Ingestive and manual
Bromeliaceae	?		Bromeliad leaf			547.09 (333.64); n = 2	783.01	Ingestive
Formicidae	?		Insects	6.56 (1.13); n = 3	7.86	845.43 (219.96); n = 3	1073.5	Ingestive and manual
Poaceae	*Saccharum* sp.		Cane	2.17 (0.94); n = 7	2.97	7838.09 (3816.06); n = 6	11,410.39	Manual
Poaceae	*Saccharum* sp.		Cane	0.15 (0.14); n = 3	0.3	2077.23 (861.43); n = 6	3215.3	Ingestive
Lecythidaceae	*Lecythis* sp.	Exocarp	Fruta d'anta			522.47 (98.19); n = 8	630.7	Manual
Lecythidaceae	*Emmotum nitens/ Lecythis* sp.	Mesocarp	Fruta d'anta	3.12 (1.11); n = 3	4.36	596.84 (46.93); n = 3	649.3	Ingestive
Lecythidaceae	*Emmotum nitens/ Lecythis* sp.	Endocarp	Fruta d'anta	4.8 (2.24); n = 3	8.66	168.6 (114.41); n = 2	249.5	Manual
Anacardiaceae	*Anacardium occidentale*	Exocarp	Fruta de caju^a^			4650.17 (241.84); n = 3	649.3	Manual
Anacardiaceae	*Anacardium occidentale*	Mesocarp	Fruta de caju			2427.83 (671.35); n = 3	2312.6	Ingestive
Anacardiaceae	*Anacardium occidentale*	Endocarp	Fruta de caju			548.1 (9.19); n = 3	554.6	Manual
Leguminosae-Caesalpinaceae	*Copaifera langsdorffi*	Mesocarp	Fruta podoin			2427.83 (2150.83); n = 3	4881.8	Ingestive
Leguminosae-Caesalpinaceae	*Copaifera langsdorffi*	Endocarp	Fruta podoin	15.29 (NA); n = 1	15.29			Manual
Leguminosae-Caesalpinaceae	*Copaifera langsdorffi*	Seed	Fruta podoin			1007.6 (300.60); n = 3	1318.6	Manual
Palmae-Arecaceae	*Orbignya* sp.	Exocarp	Piaçava	153.76 (66.61); n = 4	212.7	5010.05 (3939.77); n = 4	8792.6	Manual
Palmae-Arecaceae	*Orbignya* sp.	Mesocarp	Piaçava	68.17 (45.85); n = 10	129.61	678.30 (330.26); n = 8	1346.7	Ingestive and manual
Palmae-Arecaceae	*Orbignya* sp.	Kernel	Piaçava	6.22 (1.13); n = 5	8.16	641.21 (349.56); n = 7	1346.00	Manual
Palmae-Arecaceae	*Orbignya* sp.	Endocarp	Piaçava	16,000.62^b^				Manual (Tool-use)
Fabaceae	?		Pod	0.15 (0.08); n = 8	0.28	1176.03 (576.52); n = 5	2106.69	Manual
Fabaceae	?		Pod			1742.37 (1451.56); n = 7	2768.77	Ingestive
Poaceae	?		USO^c^			7705.26 (6177.99); n = 6	16,772.3	Manual
Poaceae	?		USO^c^			3802.62 (2342.89); n = 4	6021.05	Ingestive
Palmae-Arecaceae	*Astrocaryum campestre*	Exocarp	Tucum^d^			1532.53 (534.28); n = 6	2135.4	Manual
Palmae-Arecaceae	*Astrocaryum campestre*	Mesocarp	Tucum	23.76 (15.16); n = 4	36.85	1931.1 (523.58); n = 6	2479.5	Ingestive
Palmae-Arecaceae	*Astrocaryum campestre*	Kernel	Tucum	67.80 (21.78); n = 8	98.46	2310.28 (838.32); n = 6	3620.2	Ingestive

^a^Food material properties and behaviors were collected for the accessory hypocarp (also known as the pseudo-fruit or apple) of fruta de caju (cashew fruits).

^b^Minimum elastic modulus values for piaçava endocarp. We recorded, but do not present, several other much higher elastic modulus values for piaçava endocarp that we consider potentially anomalous.

^c^Underground storage organ (USO).

^d^We were not able to record food material properties for tucum endocarp, but this material likely has high elastic modulus values similar to piaçava endocarp.

### Food material properties

Immediately following video recording, sample foods were collected from the same source (in order of preference, branch, and tree) from which the animal obtained their food item using branch clippers or by climbing the tree. In some instances, the actual food item dropped by the capuchin was collected for testing. The capuchins were not provisioned, although the smaller group foraged for sugar cane (*Saccharum* sp.) in a small local plantation. Although food size and other geometric properties likely influence processing behavior, we were unable to reliably measure the geometric sizes of the food items in the mouths or hands.

The FMPs were measured using a Lucas Scientific FLS-1 portable mechanical tester^60–62^. Food material properties were collected from all food samples within 24 h of collection and most testing took place within 12 h of collection. All samples were stored in plastic bags at room temperature to minimize moisture loss. Toughness and elastic modulus measures were collected in the orientation in which the plant tissues were breached in order to be most relevant to food processing behaviors. For example, torn plant tissues were tested parallel to the fibers. Toughness was measured using scissor and wedge tests. In both these tests a controlled crack is propagated through the material and the energy needed to do so is estimated from measured forces and crack area^63^. Scissors tests were used to measure the toughness of thinner tissues (e.g., the exocarp of fruits), where a wedge test was used to evaluate toughness on thicker blocks of tissue (e.g., the mesocarp of fruits). Scissor and wedge tests yield moderate but significant differences in toughness measures from on the same food^64^. However, it was not possible to collect toughness measures using a single type of test for this study, and we compared toughness values from scissor and wedge tests with the caveat that some differences may be the result of testing method.

Elastic modulus was tested on most species using blunt-indent tests. This test requires that a hemispherical indenter is loaded slowly at a consistent rate for around 10 s and the resultant force is recorded. Following this initial loading, the displacement is held constant while recording the force decay for a further 90 s or until the load stabilizes. Fitting a curve to the relaxation behavior allows the calculation of both an instantaneous (E_i_) and infinite (E_∞_) elastic modulus. E_i_ represents the elastic modulus of a material if it could be loaded instantly, while E_∞_ estimates the elastic behavior under an infinitely slow loading regime. In this study, we use E_i_ measurements as these are likely more relevant when investigating how food behaves under chewing loads^65^. The elastic modulus of the extremely stiff endocarp of *Orbignya* sp. was measured using an arch test. In this test c-rings are constructed from endocarp material. These are then loaded to failure and using linear isotropic beam theory an elastic modulus can be calculated^65^.

A total of 112 toughness values was obtained from 21 different species and tissue types (e.g., exocarp, the exterior, or mesocarp, the pulp), ranging from a ‘fruta d'anta’ (*Emmotum* sp.) exocarp (522.47 ± 98.19 J m^−2^) to sugar cane (*Saccharum* sp.; 7838.09 ± 3816.06 J m^−2^; Table 3). Most foods overlapped in toughness measures, but there were some significant differences between foods (Supplementary Table S4). Seventy-nine measures of elastic modulus were recorded across 13 species and tissue types ranged from ‘bananinha’ (Fabaceae family; 0.01 ± 0.01 MPa) to the endocarp of ‘piaçava’ (*Obrignya* sp.; 16 GPa; Table 3). Elastic modulus values also overlapped for most foods (Supplementary Table S5).

### Analyses

While FMP values were obtained from most of the foods in the recorded videos, the FMP analyses have several limitations. The first hypothesis tested differences in FMPs, behavioral frequency, and behavioral duration across the feeding sequence—manual behaviors, ingestive behaviors, and mastication. Food material property values were associated with either ingestive behaviors or manual behaviors (Supplementary Table S2), and all fruit FMPs were averaged for ingestive and manual behaviors as it was not always possible to determine the fruit species in the videos. We were also not able to distinguish FMP values for ingested foods that were subsequently masticated compared to ingested foods that were discarded, therefore FMPs are not compared between ingestive behaviors and mastication. For the five species with tissue-specific measurements, FMPs were averaged for the ingested and manually manipulated classifications. For example, the mesocarp and kernel of tucum (*Astrocaryum campestre*) were both ingested, so the FMPs of these tissues were averaged for the analyses. The second and third hypotheses only examined FMP differences across foods. We were unable to associate individual FMP measures with oral or oral-manual behaviors and toothrow locations in order to test FMP differences within foods (between tissues).

All three hypotheses were tested using nested linear mixed-effect (LME) models fit by maximum likelihood. These models allow errors introduced by repeated measures, such as multiple recordings from one individual, to be dependent on each other. In each of the hypotheses, the response variable was FMP values or duration of the behavior. Explanatory variables for the first hypotheses were manual behaviors, ingestive behaviors, or mastication, ingestive behavioral classifications as oral or oral-manual for the second hypothesis, and toothrow position (anterior or posterior dentition) for the third hypothesis. Random factors were nested as, for example, individual, and food. All LME model results are available in Supplementary Table S6. The LME models were tested using post hoc Tukey comparisons performed in the R package ‘multicomp’ with a Bonferroni Holm correction to minimize type one error^66^. All analyses were performed in R version 3.6.2^67^, and significance was set at *p* < 0.05.

## Supplementary information

Supplementary Information.

## Data Availability

All raw data are available in the Supplementary Online Material.
