# Supplementary material for: Ingestive behaviors in bearded capuchins (Sapajus libidinosus)
Source: Sci Rep. 2020 Nov 30;10:20850. doi: 10.1038/s41598-020-77797-2 (PMC7705727; doi:10.1038/s41598-020-77797-2)
Supplement: Supplementary file 1 — Supplementary Information. [file 41598_2020_77797_MOESM1_ESM.docx]

**Ingestive behaviors in bearded capuchins (*Sapajus libidinosus*): Implications for the evolution of feeding adaptations in primates and fossil hominins**

Myra F. Laird^1*^, Barth W. Wright^2^, Annie O. Rivera^3^, Mariana Dutra Fogaça^4,5^, Adam van Casteren^6^, Dorothy M. Fragaszy^7^, Patricia Izar^8^, Elisabetta Visalberghi^9^, Robert S. Scott^10^, David S. Strait^6,11^, Callum F. Ross^12^, Kristin A. Wright^13^

^1^Department of Integrative Anatomical Sciences, University of Southern California, 1333 San Pablo Street, Los Angeles, CA 90033, United States

^2^Department of Anatomy, Kansas City University of Medicine and Biosciences, 1750 Independence Ave., Kansas City, MO 64106, United States

^3^Department of Anthropology, Northwestern University, 1810 Hinman Avenue, Evanston, IL 60208, United States

^4^Department of Biomedical Sciences, Institute of Population Genetics, University of Veterinary Medicine, Veterinärplatz 1, A-1210, Vienna, Austria

^5^Neotropical Primates Research Group – NeoPReGo, Manoel Oliveira Bueno, 469, São Paulo 03643-010, Brazil

^6^Department of Anthropology, Washington University in St. Louis, Campus Box 1114

One Brookings Drive, St. Louis, MO 63130, United States

^7^Department of Psychology, University of Georgia, 125 Baldwin Street, Athens, GA 30602, United States

^8^Department of Experimental Psychology, University of São Paulo, Av. Mello Moraes, 1721, São Paulo 05508-030, Brazil

^9^Institute of Cognitive Sciences and Technologies, National Research Council (CNR), Via U. Aldrovandi 16b, 00197 Rome, Italy

^10^Department of Anthropology, Rutgers University, 131 George Street, New Brunswick, NJ 08901, United States

^11^Palaeo-Research Institute, University of Johannesburg, Cnr Kingsway and University Road Auckland Park, PO Box 524 Auckland Park 2006, South Africa

^12^Department of Organismal Biology and Anatomy, University of Chicago, 1027 E 57th St.

Chicago, IL 60637, United States

^13^Department of Biomedical Sciences, University of Missouri Kansas City School of Medicine, 2411 Holmes Street, Kansas City, MO 64108, United States

**Supplementary Online Material (SOM)**

**SOM Table 1.** Definitions of terms defining primate feeding behaviors.

| **Feeding term** | | | **Definition** |
| --- | --- | --- | --- |
| Feeding event | | | All behaviors used to process a food item from manual food processing to final swallow. Includes manual and ingestive food processing behaviors and mastication. |
| - Manual (preingestive) | | | Behaviors occurring before the food item enters the oral cavity involving the limbs (e.g., rubbing or stripping a food item with the hands or tool use). |
| Feeding sequence | | | All behaviors used to process a food item from ingestion to final swallow involving the oral cavity. Includes ingestive food processing behaviors and mastication. |
| - Ingestive | | | Behaviors involving the introduction of foods to the oral cavity. Includes oral and oral-manual behaviors. |
| - Oral | | Ingestive behaviors involving the oral cavity but not the limbs (e.g., biting or stripping in which neck extension is used to alter reaction force on the food). May be subdivided by location on the toothrow. |  |
| - Oral-manual | | Ingestive behaviors involving a combination of the oral cavity and limbs; these may include biting and pulling. May be subdivided by location on the toothrow. |  |
| - Incisor | Ingestive behaviors (oral or oral-manual) using the incisors (e.g., biting or stripping a food item). |  |  |
| - Canine | Ingestive behaviors (oral or oral-manual) using the canines (e.g., puncturing a food item). |  |  |
| - Postcanine | Ingestive behaviors (oral or oral-manual) occurring on the premolars and molars (e.g., biting). |  |  |
| - Mastication | | | Chewing cycles, cyclic intraoral food processing occurring on the postcanine dentition during which the lower jaw moves upward and medially near minimum gape. |

**SOM Table S2**

Behavioral data and classifications. Abbreviations: USO = underground storage organs.

| **Video name** | **KW ID** | **Age/**  **sex** | **Feeding sequence** | **Food** | **Start frame** | **End frame** | **Duration (seconds)** | **H1 classifications** | **H2 classifications** | **H3 classifications** |
| --- | --- | --- | --- | --- | --- | --- | --- | --- | --- | --- |
| ‘B5IL4F~K’ | Dita | AF | Sequence 1 | Piaçava | 629 | 757 | 4.27 | Manual |  |  |
| ‘B5IL4F~K’ | Dita | AF | Sequence 1 | Piaçava | 757 | 770 | 0.43 | Ingestive | Oral-Manual | Anterior |
| ‘B5IL4F~K’ | Dita | AF | Sequence 1 | Piaçava | 770 | 888 | 3.93 | Manual |  |  |
| ‘B5IL4F~K’ | Dita | AF | Sequence 2 | Piaçava | 924 | 948 | 0.80 | Manual |  |  |
| ‘B5IL4F~K’ | Dita | AF | Sequence 2 | Piaçava | 948 | 962 | 0.47 | Ingestive | Oral-Manual | Anterior |
| ‘B5IL4F~K’ | Dita | AF | Sequence 2 | Piaçava | 962 | 1020 | 1.93 | Masticate |  |  |
| ‘B5IL4F~K’ | Dita | AF | Sequence 2 | Piaçava | 1020 | 1051 | 1.03 | Ingestive | Oral-Manual | Anterior |
| ‘B5IL4F~K’ | Dita | AF | Sequence 2 | Piaçava | 1051 | 1106 | 1.83 | Masticate |  |  |
| ‘B5IL4F~K’ | Dita | AF | Sequence 2 | Piaçava | 1106 | 1122 | 0.53 | Ingestive | Oral-Manual | Anterior |
| ‘B5IL4F~K’ | Dita | AF | Sequence 2 | Piaçava | 1122 | 1220 | 3.27 | Masticate |  |  |
| ‘B5IL4F~K’ | Dita | AF | Sequence 3 | Piaçava | 1261 | 1371 | 3.67 | Manual |  |  |
| ‘B5IL4F~K’ | Dita | AF | Sequence 3 | Piaçava | 1371 | 1381 | 0.33 | Ingestive | Oral-Manual | Anterior |
| ‘B5IL4F~K’ | Dita | AF | Sequence 3 | Piaçava | 1381 | 1413 | 1.07 | Manual |  |  |
| ‘B5IL4F~K’ | Dita | AF | Sequence 3 | Piaçava | 1413 | 1442 | 0.97 | Ingestive | Oral-Manual | Anterior |
| ‘B5IL4F~K’ | Dita | AF | Sequence 3 | Piaçava | 1517 | 1538 | 0.70 | Ingestive | Oral-Manual | Anterior |
| ‘B5IL4F~K’ | Dita | AF | Sequence 3 | Piaçava | 1700 | 1717 | 0.57 | Ingestive | Oral-Manual | Anterior |
| ‘B5IL4F~K’ | Dita | AF | Sequence 3 | Piaçava | 1717 | 1766 | 1.63 | Masticate |  |  |
| ‘B5IL4F~K’ | Dita | AF | Sequence 3 | Piaçava | 1766 | 1796 | 1.00 | Ingestive | Oral-Manual | Anterior |
| ‘B5IL4F~K’ | Dita | AF | Sequence 3 | Piaçava | 1796 | 1972 | 5.87 | Masticate |  |  |
| ‘B5IL4F~K’ | Dita | AF | Sequence 3 | Piaçava | 1972 | 2011 | 1.30 | Ingestive | Oral-Manual | Anterior |
| ‘B5IL4F~K’ | Dita | AF | Sequence 3 | Piaçava | 2117 | 2154 | 1.23 | Ingestive | Oral-Manual | Anterior |
| ‘B5IL4F~K’ | Dita | AF | Sequence 3 | Piaçava | 2175 | 2182 | 0.23 | Ingestive | Oral-Manual | Anterior |
| ‘B5IL4F~K’ | Dita | AF | Sequence 3 | Piaçava | 2182 | 2231 | 1.63 | Masticate |  |  |
| ‘B5IL4F~K’ | Dita | AF | Sequence 3 | Piaçava | 2231 | 2238 | 0.23 | Ingestive | Oral-Manual | Anterior |
| ‘big tuber’ | Chuchu | AF | Sequence 1 | USO | 304 | 321 | 0.57 | Manual |  |  |
| ‘big tuber’ | Chuchu | AF | Sequence 1 | USO | 321 | 347 | 0.87 | Ingestive | Oral-Manual | Anterior |
| ‘big tuber’ | Chuchu | AF | Sequence 1 | USO | 347 | 412 | 2.17 | Masticate |  |  |
| ‘big tuber’ | Chuchu | AF | Sequence 1 | USO | 412 | 437 | 0.83 | Ingestive | Oral-Manual | Anterior |
| ‘big tuber’ | Chuchu | AF | Sequence 1 | USO | 437 | 507 | 2.33 | Masticate |  |  |
| ‘big tuber’ | Chuchu | AF | Sequence 1 | USO | 507 | 522 | 0.50 | Ingestive | Oral-Manual | Anterior |
| ‘big tuber’ | Chuchu | AF | Sequence 1 | USO | 522 | 545 | 0.77 | Ingestive | Oral-Manual | Anterior |
| ‘big tuber’ | Chuchu | AF | Sequence 2 | USO | 590 | 612 | 0.73 | Ingestive | Oral-Manual | Anterior |
| ‘big tuber’ | Chuchu | AF | Sequence 2 | USO | 642 | 691 | 1.63 | Ingestive | Oral-Manual | Posterior |
| ‘big tuber’ | Chuchu | AF | Sequence 2 | USO | 691 | 729 | 1.27 | Masticate |  |  |
| ‘big tuber’ | Chuchu | AF | Sequence 2 | USO | 729 | 739 | 0.33 | Ingestive | Oral-Manual | Posterior |
| ‘big tuber’ | Chuchu | AF | Sequence 2 | USO | 739 | 799 | 2.00 | Masticate |  |  |
| ‘big tuber’ | Chuchu | AF | Sequence 3 | USO | 857 | 882 | 0.83 | Ingestive | Oral-Manual | Posterior |
| ‘big tuber’ | Chuchu | AF | Sequence 3 | USO | 882 | 940 | 1.93 | Masticate |  |  |
| ‘big tuber’ | Chuchu | AF | Sequence 3 | USO | 940 | 946 | 0.20 | Ingestive | Oral-Manual | Anterior |
| ‘big tuber’ | Chuchu | AF | Sequence 3 | USO | 946 | 992 | 1.53 | Masticate |  |  |
| ‘big tuber’ | Chuchu | AF | Sequence 3 | USO | 992 | 1042 | 1.67 | Ingestive | Oral-Manual | Anterior |
| ‘big tuber’ | Chuchu | AF | Sequence 3 | USO | 1042 | 1072 | 1.00 | Ingestive | Oral-Manual | Anterior |
| ‘big tuber’ | Chuchu | AF | Sequence 3 | USO | 1104 | 1124 | 0.67 | Ingestive | Oral-Manual | Anterior |
| ‘big tuber’ | Chuchu | AF | Sequence 3 | USO | 1124 | 1174 | 1.67 | Masticate |  |  |
| ‘big tuber’ | Chuchu | AF | Sequence 3 | USO | 1174 | 1189 | 0.50 | Ingestive | Oral-Manual | Anterior |
| ‘big tuber’ | Chuchu | AF | Sequence 3 | USO | 1308 | 1326 | 0.60 | Ingestive | Oral-Manual | Anterior |
| ‘big tuber’ | Chuchu | AF | Sequence 3 | USO | 1441 | 1445 | 0.13 | Ingestive | Oral-Manual | Anterior |
| ‘biig tuber’ | Presente | SAM | Sequence 1 | USO | 472 | 494 | 0.73 | Ingestive | Oral-Manual | Anterior |
| ‘biig tuber’ | Presente | SAM | Sequence 1 | USO | 17 | 31 | 0.47 | Ingestive | Oral | Anterior |
| ‘biig tuber’ | Presente | SAM | Sequence 1 | USO | 129 | 149 | 0.67 | Ingestive | Oral-Manual | Anterior |
| ‘biig tuber’ | Presente | SAM | Sequence 1 | USO | 149 | 163 | 0.47 | Masticate |  |  |
| ‘biig tuber’ | Presente | SAM | Sequence 1 | USO | 163 | 341 | 5.93 | Ingestive | Oral-Manual | Anterior |
| ‘biig tuber’ | Presente | SAM | Sequence 1 | USO | 644 | 673 | 0.97 | Ingestive | Oral-Manual | Anterior |
| ‘biig tuber’ | Presente | SAM | Sequence 1 | USO | 1349 | 1382 | 1.10 | Ingestive | Oral | Anterior |
| ‘biig tuber’ | Presente | SAM | Sequence 1 | USO | 1495 | 1632 | 4.57 | Manual |  |  |
| ‘biig tuber’ | Presente | SAM | Sequence 1 | USO | 1632 | 1666 | 1.13 | Ingestive | Oral-Manual | Anterior |
| ‘biig tuber’ | Presente | SAM | Sequence 1 | USO | 1684 | 1711 | 0.90 | Ingestive | Oral-Manual | Anterior |
| ‘biig tuber’ | Presente | SAM | Sequence 1 | USO | 1727 | 1762 | 1.17 | Ingestive | Oral-Manual | Anterior |
| ‘biig tuber’ | Presente | SAM | Sequence 1 | USO | 1774 | 1789 | 0.50 | Ingestive | Oral-Manual | Anterior |
| ‘biig tuber’ | Presente | SAM | Sequence 1 | USO | 1789 | 1807 | 0.60 | Masticate |  |  |
| ‘biig tuber’ | Presente | SAM | Sequence 1 | USO | 1807 | 1823 | 0.53 | Ingestive | Oral-Manual | Anterior |
| ‘biig tuber’ | Presente | SAM | Sequence 1 | USO | 1823 | 1910 | 2.90 | Masticate |  |  |
| ‘biig tuber’ | Presente | SAM | Sequence 1 | USO | 1910 | 1926 | 0.53 | Ingestive | Oral-Manual | Anterior |
| ‘biig tuber’ | Presente | SAM | Sequence 1 | USO | 1926 | 1952 | 0.87 | Masticate |  |  |
| ‘biig tuber’ | Presente | SAM | Sequence 1 | USO | 1952 | 1970 | 0.60 | Ingestive | Oral-Manual | Anterior |
| ‘biig tuber’ | Presente | SAM | Sequence 1 | USO | 1970 | 2109 | 4.63 | Masticate |  |  |
| ‘biig tuber’ | Presente | SAM | Sequence 1 | USO | 2109 | 2141 | 1.07 | Ingestive | Oral-Manual | Anterior |
| ‘biig tuber’ | Presente | SAM | Sequence 1 | USO | 2141 | 2154 | 0.43 | Ingestive | Oral-Manual | Posterior |
| ‘biig tuber’ | Presente | SAM | Sequence 1 | USO | 2154 | 2210 | 1.87 | Masticate |  |  |
| ‘biig tuber’ | Presente | SAM | Sequence 1 | USO | 2210 | 2223 | 0.43 | Ingestive | Oral-Manual | Posterior |
| ‘biig tuber’ | Presente | SAM | Sequence 1 | USO | 2223 | 2248 | 0.83 | Masticate |  |  |
| ‘biig tuber’ | Presente | SAM | Sequence 1 | USO | 2248 | 2276 | 0.93 | Ingestive | Oral-Manual | Anterior |
| ‘biig tuber’ | Presente | SAM | Sequence 1 | USO | 2276 | 2415 | 4.63 | Masticate |  |  |
| ‘biig tuber’ | Presente | SAM | Sequence 1 | USO | 2415 | 2445 | 1.00 | Ingestive | Oral-Manual | Anterior |
| ‘biig tuber’ | Presente | SAM | Sequence 1 | USO | 2445 | 2470 | 0.83 | Masticate |  |  |
| ‘biig tuber’ | Presente | SAM | Sequence 1 | USO | 2470 | 2485 | 0.50 | Ingestive | Oral-Manual | Anterior |
| ‘biig tuber’ | Presente | SAM | Sequence 1 | USO | 2485 | 2553 | 2.27 | Masticate |  |  |
| ‘biig tuber’ | Presente | SAM | Sequence 1 | USO | 2553 | 2568 | 0.50 | Ingestive | Oral-Manual | Posterior |
| ‘biig tuber’ | Presente | SAM | Sequence 1 | USO | 2568 | 2630 | 2.07 | Masticate |  |  |
| ‘biig tuber’ | Presente | SAM | Sequence 1 | USO | 2630 | 2669 | 1.30 | Ingestive | Oral-Manual | Posterior |
| ‘biig tuber’ | Presente | SAM | Sequence 1 | USO | 2878 | 2925 | 1.57 | Ingestive | Oral-Manual | Posterior |
| ‘biig tuber’ | Presente | SAM | Sequence 1 | USO | 2925 | 2945 | 0.67 | Masticate |  |  |
| ‘biig tuber’ | Presente | SAM | Sequence 1 | USO | 2945 | 2970 | 0.83 | Ingestive | Oral-Manual | Posterior |
| ‘biig tuber’ | Presente | SAM | Sequence 1 | USO | 2970 | 2997 | 0.90 | Masticate |  |  |
| ‘biig tuber’ | Presente | SAM | Sequence 1 | USO | 2997 | 3004 | 0.23 | Ingestive | Oral-Manual | Posterior |
| ‘biig tuber’ | Presente | SAM | Sequence 1 | USO | 3126 | 3142 | 0.53 | Ingestive | Oral | Anterior |
| ‘biig tuber’ | Presente | SAM | Sequence 1 | USO | 3363 | 3402 | 1.30 | Ingestive | Oral | Anterior |
| ‘biig tuber’ | Presente | SAM | Sequence 1 | USO | 3438 | 3490 | 1.73 | Ingestive | Oral-Manual | Anterior |
| ‘biig tuber’ | Presente | SAM | Sequence 1 | USO | 3505 | 3537 | 1.07 | Ingestive | Oral-Manual | Anterior |
| ‘biig tuber’ | Presente | SAM | Sequence 1 | USO | 3546 | 3550 | 0.13 | Ingestive | Oral-Manual | Posterior |
| ‘biig tuber’ | Presente | SAM | Sequence 1 | USO | 3550 | 3570 | 0.67 | Ingestive | Oral-Manual | Anterior |
| ‘biig tuber’ | Presente | SAM | Sequence 1 | USO | 3570 | 3604 | 1.13 | Ingestive | Oral-Manual | Posterior |
| ‘biig tuber’ | Presente | SAM | Sequence 1 | USO | 3652 | 3676 | 0.80 | Ingestive | Oral-Manual | Anterior |
| ‘biig tuber’ | Presente | SAM | Sequence 1 | USO | 3720 | 3735 | 0.50 | Ingestive | Oral-Manual | Anterior |
| ‘biig tuber’ | Presente | SAM | Sequence 1 | USO | 3758 | 3778 | 0.67 | Ingestive | Oral-Manual | Anterior |
| ‘biig tuber’ | Presente | SAM | Sequence 1 | USO | 3778 | 3801 | 0.77 | Ingestive | Oral-Manual | Anterior |
| ‘biig tuber’ | Presente | SAM | Sequence 1 | USO | 3801 | 3814 | 0.43 | Ingestive | Oral-Manual | Anterior |
| ‘biig tuber’ | Presente | SAM | Sequence 1 | USO | 3814 | 3862 | 1.60 | Ingestive | Oral-Manual | Posterior |
| ‘biig tuber’ | Presente | SAM | Sequence 1 | USO | 4383 | 4399 | 0.53 | Ingestive | Oral | Anterior |
| ‘biig tuber’ | Presente | SAM | Sequence 1 | USO | 5326 | 5328 | 0.07 | Ingestive | Oral | Anterior |
| ‘biig tuber’ | Presente | SAM | Sequence 1 | USO | 5688 | 5709 | 0.70 | Ingestive | Oral | Anterior |
| ‘biig tuber’ | Presente | SAM | Sequence 1 | USO | 5709 | 5943 | 7.80 | Manual |  |  |
| ‘biig tuber’ | Presente | SAM | Sequence 1 | USO | 5943 | 5982 | 1.30 | Ingestive | Oral-Manual | Anterior |
| ‘biig tuber’ | Presente | SAM | Sequence 1 | USO | 6036 | 6056 | 0.67 | Ingestive | Oral-Manual | Anterior |
| ‘biig tuber’ | Presente | SAM | Sequence 1 | USO | 6120 | 6149 | 0.97 | Ingestive | Oral-Manual | Anterior |
| ‘biig tuber’ | Presente | SAM | Sequence 1 | USO | 6190 | 6233 | 1.43 | Ingestive | Oral-Manual | Posterior |
| ‘biig tuber’ | Presente | SAM | Sequence 1 | USO | 6268 | 6299 | 1.03 | Ingestive | Oral-Manual | Posterior |
| ‘biig tuber’ | Presente | SAM | Sequence 1 | USO | 6336 | 6347 | 0.37 | Ingestive | Oral-Manual | Anterior |
| ‘biig tuber’ | Presente | SAM | Sequence 1 | USO | 6365 | 6375 | 0.33 | Ingestive | Oral-Manual | Anterior |
| ‘biig tuber’ | Presente | SAM | Sequence 1 | USO | 6375 | 6400 | 0.83 | Ingestive | Oral-Manual | Anterior |
| ‘biig tuber’ | Presente | SAM | Sequence 1 | USO | 6400 | 6424 | 0.80 | Ingestive | Oral-Manual | Anterior |
| ‘biig tuber’ | Presente | SAM | Sequence 1 | USO | 6446 | 6467 | 0.70 | Ingestive | Oral-Manual | Anterior |
| ‘biig tuber’ | Presente | SAM | Sequence 1 | USO | 6484 | 6520 | 1.20 | Ingestive | Oral-Manual | Anterior |
| ‘biig tuber’ | Presente | SAM | Sequence 1 | USO | 6520 | 6950 | 14.33 | Masticate |  |  |
| ‘biig tuber’ | Presente | SAM | Sequence 1 | USO | 6950 | 6963 | 0.43 | Ingestive | Oral-Manual | Anterior |
| ‘biig tuber’ | Presente | SAM | Sequence 1 | USO | 6963 | 7023 | 2.00 | Masticate |  |  |
| ‘biig tuber’ | Presente | SAM | Sequence 1 | USO | 7023 | 7039 | 0.53 | Ingestive | Oral-Manual | Anterior |
| ‘biig tuber’ | Presente | SAM | Sequence 1 | USO | 7067 | 7082 | 0.50 | Ingestive | Oral-Manual | Anterior |
| ‘biig tuber’ | Presente | SAM | Sequence 1 | USO | 7082 | 7297 | 7.17 | Ingestive | Oral-Manual | Posterior |
| ‘biig tuber’ | Presente | SAM | Sequence 1 | USO | 7316 | 7357 | 1.37 | Ingestive | Oral-Manual | Posterior |
| ‘biig tuber’ | Presente | SAM | Sequence 1 | USO | 7427 | 7436 | 0.30 | Ingestive | Oral-Manual | Anterior |
| ‘biig tuber’ | Presente | SAM | Sequence 1 | USO | 7436 | 7627 | 6.37 | Masticate |  |  |
| ‘biig tuber’ | Presente | SAM | Sequence 1 | USO | 7627 | 7635 | 0.27 | Ingestive | Oral-Manual | Anterior |
| ‘biig tuber’ | Presente | SAM | Sequence 1 | USO | 7635 | 7767 | 4.40 | Masticate |  |  |
| ‘biig tuber’ | Presente | SAM | Sequence 1 | USO | 7767 | 7784 | 0.57 | Ingestive | Oral-Manual | Anterior |
| ‘biig tuber’ | Presente | SAM | Sequence 1 | USO | 7784 | 8226 | 14.73 | Masticate |  |  |
| ‘biig tuber’ | Presente | SAM | Sequence 1 | USO | 8226 | 8270 | 1.47 | Ingestive | Oral-Manual | Anterior |
| ‘biig tuber’ | Presente | SAM | Sequence 1 | USO | 8310 | 8317 | 0.23 | Ingestive | Oral-Manual | Anterior |
| ‘biig tuber’ | Presente | SAM | Sequence 1 | USO | 8317 | 8523 | 6.87 | Masticate |  |  |
| ‘biig tuber’ | Presente | SAM | Sequence 1 | USO | 8523 | 8543 | 0.67 | Ingestive | Oral-Manual | Anterior |
| ‘biig tuber’ | Presente | SAM | Sequence 1 | USO | 8553 | 8559 | 0.20 | Ingestive | Oral-Manual | Posterior |
| ‘biig tuber’ | Presente | SAM | Sequence 1 | USO | 8576 | 8657 | 2.70 | Ingestive | Oral-Manual | Posterior |
| ‘biig tuber’ | Presente | SAM | Sequence 1 | USO | 8670 | 8872 | 6.73 | Ingestive | Oral-Manual | Posterior |
| ‘biig tuber’ | Presente | SAM | Sequence 1 | USO | 8910 | 8920 | 0.33 | Ingestive | Oral-Manual | Anterior |
| ‘biig tuber’ | Presente | SAM | Sequence 1 | USO | 8920 | 8960 | 1.33 | Masticate |  |  |
| ‘biig tuber’ | Presente | SAM | Sequence 1 | USO | 8960 | 8987 | 0.90 | Ingestive | Oral-Manual | Anterior |
| ‘biig tuber’ | Presente | SAM | Sequence 1 | USO | 8987 | 9007 | 0.67 | Ingestive | Oral-Manual | Posterior |
| ‘biig tuber’ | Presente | SAM | Sequence 1 | USO | 9007 | 9066 | 1.97 | Masticate |  |  |
| ‘biig tuber’ | Presente | SAM | Sequence 1 | USO | 9066 | 9081 | 0.50 | Ingestive | Oral-Manual | Anterior |
| ‘biig tuber’ | Presente | SAM | Sequence 1 | USO | 9081 | 9572 | 16.37 | Masticate |  |  |
| ‘biig tuber’ | Presente | SAM | Sequence 1 | USO | 9572 | 9586 | 0.47 | Ingestive | Oral-Manual | Anterior |
| ‘biig tuber’ | Presente | SAM | Sequence 1 | USO | 9586 | 9851 | 8.83 | Masticate |  |  |
| ‘biig tuber’ | Presente | SAM | Sequence 1 | USO | 9851 | 9897 | 1.53 | Ingestive | Oral-Manual | Anterior |
| ‘biig tuber’ | Presente | SAM | Sequence 1 | USO | 9923 | 9941 | 0.60 | Ingestive | Oral-Manual | Anterior |
| ‘biig tuber’ | Presente | SAM | Sequence 1 | USO | 9941 | 10013 | 2.40 | Masticate |  |  |
| ‘biig tuber’ | Presente | SAM | Sequence 1 | USO | 10013 | 10022 | 0.30 | Ingestive | Oral-Manual | Anterior |
| ‘biig tuber’ | Presente | SAM | Sequence 1 | USO | 10045 | 10050 | 0.17 | Masticate |  |  |
| ‘biig tuber’ | Presente | SAM | Sequence 1 | USO | 10050 | 10097 | 1.57 | Ingestive | Oral-Manual | Posterior |
| ‘biig tuber’ | Presente | SAM | Sequence 1 | USO | 10097 | 10114 | 0.57 | Masticate |  |  |
| ‘biig tuber’ | Presente | SAM | Sequence 1 | USO | 10114 | 10183 | 2.30 | Ingestive | Oral-Manual | Posterior |
| ‘biig tuber’ | Presente | SAM | Sequence 1 | USO | 10183 | 10214 | 1.03 | Masticate |  |  |
| ‘biig tuber’ | Presente | SAM | Sequence 1 | USO | 10214 | 10225 | 0.37 | Ingestive | Oral-Manual | Anterior |
| ‘biig tuber’ | Presente | SAM | Sequence 1 | USO | 10225 | 10265 | 1.33 | Masticate |  |  |
| ‘biig tuber’ | Presente | SAM | Sequence 1 | USO | 10265 | 10297 | 1.07 | Ingestive | Oral-Manual | Anterior |
| ‘biig tuber’ | Presente | SAM | Sequence 1 | USO | 10297 | 11025 | 24.27 | Masticate |  |  |
| ‘biig tuber’ | Presente | SAM | Sequence 1 | USO | 11126 | 11165 | 1.30 | Ingestive | Oral-Manual | Anterior |
| ‘biig tuber’ | Presente | SAM | Sequence 1 | USO | 11165 | 11381 | 7.20 | Masticate |  |  |
| ‘biig tuber’ | Presente | SAM | Sequence 1 | USO | 11381 | 11403 | 0.73 | Ingestive | Oral-Manual | Anterior |
| ‘biig tuber’ | Presente | SAM | Sequence 1 | USO | 11403 | 11566 | 5.43 | Masticate |  |  |
| ‘biig tuber’ | Presente | SAM | Sequence 1 | USO | 11566 | 11592 | 0.87 | Ingestive | Oral-Manual | Anterior |
| ‘biig tuber’ | Presente | SAM | Sequence 1 | USO | 11592 | 11701 | 3.63 | Masticate |  |  |
| ‘biig tuber’ | Presente | SAM | Sequence 1 | USO | 11701 | 11725 | 0.80 | Ingestive | Oral-Manual | Anterior |
| ‘biig tuber’ | Presente | SAM | Sequence 1 | USO | 11755 | 11778 | 0.77 | Ingestive | Oral-Manual | Anterior |
| ‘biig tuber’ | Presente | SAM | Sequence 1 | USO | 11084 | 11811 | 24.23 | Ingestive | Oral-Manual | Anterior |
| ‘biig tuber’ | Presente | SAM | Sequence 1 | USO | 11811 | 12000 | 6.30 | Masticate |  |  |
| ‘biig tuber’ | Presente | SAM | Sequence 1 | USO | 12000 | 12023 | 0.77 | Ingestive | Oral-Manual | Anterior |
| ‘biig tuber’ | Presente | SAM | Sequence 1 | USO | 12064 | 12091 | 0.90 | Ingestive | Oral-Manual | Anterior |
| ‘biig tuber’ | Presente | SAM | Sequence 1 | USO | 12091 | 12412 | 10.70 | Masticate |  |  |
| ‘biig tuber’ | Presente | SAM | Sequence 1 | USO | 12412 | 12429 | 0.57 | Ingestive | Oral-Manual | Anterior |
| ‘biig tuber’ | Presente | SAM | Sequence 1 | USO | 12429 | 12546 | 3.90 | Masticate |  |  |
| ‘biig tuber’ | Presente | SAM | Sequence 1 | USO | 12546 | 12559 | 0.43 | Ingestive | Oral-Manual | Anterior |
| ‘biig tuber’ | Presente | SAM | Sequence 1 | USO | 12559 | 12965 | 13.53 | Masticate |  |  |
| ‘biig tuber’ | Presente | SAM | Sequence 1 | USO | 12965 | 12991 | 0.87 | Ingestive | Oral-Manual | Anterior |
| ‘biig tuber’ | Presente | SAM | Sequence 1 | USO | 12991 | 13143 | 5.07 | Masticate |  |  |
| ‘biig tuber’ | Presente | SAM | Sequence 1 | USO | 13143 | 13149 | 0.20 | Ingestive | Oral-Manual | Anterior |
| ‘biig tuber’ | Presente | SAM | Sequence 1 | USO | 13149 | 13201 | 1.73 | Masticate |  |  |
| ‘biig tuber’ | Presente | SAM | Sequence 1 | USO | 13201 | 13225 | 0.80 | Ingestive | Oral-Manual | Anterior |
| ‘biig tuber’ | Presente | SAM | Sequence 1 | USO | 13225 | 13302 | 2.57 | Masticate |  |  |
| ‘biig tuber’ | Presente | SAM | Sequence 1 | USO | 13302 | 13339 | 1.23 | Ingestive | Oral-Manual | Anterior |
| ‘biig tuber’ | Presente | SAM | Sequence 1 | USO | 13339 | 13408 | 2.30 | Masticate |  |  |
| ‘biig tuber’ | Presente | SAM | Sequence 1 | USO | 13408 | 13447 | 1.30 | Ingestive | Oral-Manual | Anterior |
| ‘biig tuber’ | Presente | SAM | Sequence 1 | USO | 13447 | 13664 | 7.23 | Masticate |  |  |
| ‘biig tuber’ | Presente | SAM | Sequence 1 | USO | 13664 | 13684 | 0.67 | Ingestive | Oral-Manual | Anterior |
| ‘biig tuber’ | Presente | SAM | Sequence 1 | USO | 13684 | 13869 | 6.17 | Masticate |  |  |
| ‘biig tuber’ | Presente | SAM | Sequence 1 | USO | 13869 | 13884 | 0.50 | Ingestive | Oral-Manual | Anterior |
| ‘biig tuber’ | Presente | SAM | Sequence 1 | USO | 13884 | 14172 | 9.60 | Masticate |  |  |
| ‘biig tuber’ | Presente | SAM | Sequence 1 | USO | 14172 | 14202 | 1.00 | Ingestive | Oral-Manual | Anterior |
| ‘biig tuber’ | Presente | SAM | Sequence 1 | USO | 14202 | 14243 | 1.37 | Masticate |  |  |
| ‘biig tuber’ | Presente | SAM | Sequence 1 | USO | 14243 | 14262 | 0.63 | Ingestive | Oral-Manual | Anterior |
| ‘biig tuber’ | Presente | SAM | Sequence 1 | USO | 14262 | 14294 | 1.07 | Masticate |  |  |
| ‘biig tuber’ | Presente | SAM | Sequence 1 | USO | 14294 | 14311 | 0.57 | Ingestive | Oral-Manual | Anterior |
| ‘biig tuber’ | Presente | SAM | Sequence 1 | USO | 14311 | 14507 | 6.53 | Masticate |  |  |
| ‘biig tuber’ | Presente | SAM | Sequence 1 | USO | 14507 | 14515 | 0.27 | Ingestive | Oral-Manual | Anterior |
| ‘biig tuber’ | Presente | SAM | Sequence 1 | USO | 14536 | 14563 | 0.90 | Ingestive | Oral-Manual | Anterior |
| ‘biig tuber’ | Presente | SAM | Sequence 1 | USO | 14563 | 15167 | 20.13 | Masticate |  |  |
| ‘biig tuber’ | Presente | SAM | Sequence 1 | USO | 15167 | 15195 | 0.93 | Ingestive | Oral-Manual | Anterior |
| ‘biig tuber’ | Presente | SAM | Sequence 1 | USO | 15195 | 15714 | 17.30 | Masticate |  |  |
| ‘biig tuber’ | Presente | SAM | Sequence 1 | USO | 15714 | 15796 | 2.73 | Ingestive | Oral-Manual | Anterior |
| ‘biig tuber’ | Presente | SAM | Sequence 1 | USO | 15796 | 15982 | 6.20 | Masticate |  |  |
| ‘biig tuber’ | Presente | SAM | Sequence 1 | USO | 15982 | 16199 | 7.23 | Ingestive | Oral-Manual | Posterior |
| ‘biig tuber’ | Presente | SAM | Sequence 1 | USO | 16199 | 16233 | 1.13 | Masticate |  |  |
| ‘biig tuber’ | Presente | SAM | Sequence 1 | USO | 16233 | 16245 | 0.40 | Ingestive | Oral-Manual | Posterior |
| ‘biig tuber’ | Presente | SAM | Sequence 1 | USO | 16245 | 16318 | 2.43 | Ingestive | Oral-Manual | Posterior |
| ‘biig tuber’ | Presente | SAM | Sequence 1 | USO | 16318 | 16354 | 1.20 | Masticate |  |  |
| ‘biig tuber’ | Presente | SAM | Sequence 1 | USO | 16354 | 16383 | 0.97 | Ingestive | Oral-Manual | Anterior |
| ‘biig tuber’ | Presente | SAM | Sequence 1 | USO | 16383 | 16857 | 15.80 | Masticate |  |  |
| ‘biig tuber’ | Presente | SAM | Sequence 1 | USO | 16857 | 16899 | 1.40 | Ingestive | Oral-Manual | Anterior |
| ‘biig tuber’ | Presente | SAM | Sequence 1 | USO | 16899 | 16922 | 0.77 | Masticate |  |  |
| ‘biig tuber’ | Presente | SAM | Sequence 1 | USO | 16922 | 16955 | 1.10 | Ingestive | Oral-Manual | Anterior |
| ‘biig tuber’ | Presente | SAM | Sequence 1 | USO | 16955 | 16971 | 0.53 | Masticate |  |  |
| ‘biig tuber’ | Presente | SAM | Sequence 1 | USO | 16971 | 16978 | 0.23 | Ingestive | Oral-Manual | Anterior |
| ‘biig tuber’ | Presente | SAM | Sequence 1 | USO | 16978 | 17130 | 5.07 | Masticate |  |  |
| ‘biig tuber’ | Presente | SAM | Sequence 1 | USO | 17130 | 17147 | 0.57 | Ingestive | Oral-Manual | Anterior |
| ‘biig tuber’ | Presente | SAM | Sequence 1 | USO | 17147 | 17288 | 4.70 | Masticate |  |  |
| ‘biig tuber’ | Presente | SAM | Sequence 1 | USO | 17288 | 17303 | 0.50 | Ingestive | Oral-Manual | Posterior |
| ‘biig tuber’ | Presente | SAM | Sequence 1 | USO | 17303 | 17481 | 5.93 | Masticate |  |  |
| ‘biig tuber’ | Presente | SAM | Sequence 1 | USO | 17481 | 17503 | 0.73 | Ingestive | Oral-Manual | Posterior |
| ‘biig tuber’ | Presente | SAM | Sequence 1 | USO | 17503 | 17536 | 1.10 | Masticate |  |  |
| ‘biig tuber’ | Presente | SAM | Sequence 1 | USO | 17536 | 17564 | 0.93 | Ingestive | Oral-Manual | Anterior |
| ‘biig tuber’ | Presente | SAM | Sequence 1 | USO | 17564 | 17640 | 2.53 | Masticate |  |  |
| ‘biig tuber’ | Presente | SAM | Sequence 1 | USO | 17640 | 17673 | 1.10 | Ingestive | Oral-Manual | Anterior |
| ‘biig tuber’ | Presente | SAM | Sequence 1 | USO | 17673 | 17790 | 3.90 | Masticate |  |  |
| ‘biig tuber’ | Presente | SAM | Sequence 1 | USO | 17790 | 17822 | 1.07 | Ingestive | Oral-Manual | Anterior |
| ‘biig tuber’ | Presente | SAM | Sequence 1 | USO | 17822 | 17857 | 1.17 | Ingestive | Oral-Manual | Posterior |
| ‘biig tuber’ | Presente | SAM | Sequence 1 | USO | 17857 | 17918 | 2.03 | Masticate |  |  |
| ‘biig tuber’ | Presente | SAM | Sequence 1 | USO | 17918 | 17949 | 1.03 | Ingestive | Oral-Manual | Posterior |
| ‘biig tuber’ | Presente | SAM | Sequence 1 | USO | 17949 | 17962 | 0.43 | Masticate |  |  |
| ‘biig tuber’ | Presente | SAM | Sequence 1 | USO | 17962 | 17979 | 0.57 | Ingestive | Oral-Manual | Anterior |
| ‘biig tuber’ | Presente | SAM | Sequence 1 | USO | 17979 | 18022 | 1.43 | Masticate |  |  |
| ‘biig tuber’ | Presente | SAM | Sequence 1 | USO | 18022 | 18136 | 3.80 | Ingestive | Oral-Manual | Posterior |
| ‘biig tuber’ | Presente | SAM | Sequence 1 | USO | 18136 | 18259 | 4.10 | Masticate |  |  |
| ‘biig tuber’ | Presente | SAM | Sequence 1 | USO | 18259 | 18315 | 1.87 | Ingestive | Oral-Manual | Posterior |
| ‘biig tuber’ | Presente | SAM | Sequence 1 | USO | 18315 | 18343 | 0.93 | Masticate |  |  |
| ‘biig tuber’ | Presente | SAM | Sequence 1 | USO | 18343 | 18348 | 0.17 | Ingestive | Oral-Manual | Anterior |
| ‘biig tuber’ | Presente | SAM | Sequence 1 | USO | 18428 | 18481 | 1.77 | Ingestive | Oral-Manual | Anterior |
| ‘biig tuber’ | Presente | SAM | Sequence 1 | USO | 18782 | 18854 | 2.40 | Ingestive | Oral-Manual | Anterior |
| ‘biig tuber’ | Presente | SAM | Sequence 1 | USO | 18871 | 18895 | 0.80 | Ingestive | Oral-Manual | Anterior |
| ‘biig tuber’ | Presente | SAM | Sequence 1 | USO | 19049 | 19072 | 0.77 | Ingestive | Oral-Manual | Posterior |
| ‘biig tuber’ | Presente | SAM | Sequence 1 | USO | 19072 | 19145 | 2.43 | Masticate |  |  |
| ‘biig tuber’ | Presente | SAM | Sequence 1 | USO | 19145 | 19205 | 2.00 | Ingestive | Oral-Manual | Anterior |
| ‘biig tuber’ | Presente | SAM | Sequence 1 | USO | 19616 | 19622 | 0.20 | Ingestive | Oral-Manual | Anterior |
| ‘biig tuber’ | Presente | SAM | Sequence 1 | USO | 19636 | 19668 | 1.07 | Ingestive | Oral-Manual | Posterior |
| ‘biig tuber’ | Presente | SAM | Sequence 1 | USO | 19668 | 19826 | 5.27 | Masticate |  |  |
| ‘biig tuber’ | Presente | SAM | Sequence 1 | USO | 19826 | 19828 | 0.07 | Ingestive | Oral-Manual | Anterior |
| ‘brom flowers ok_1’ | Divina | SAF | Sequence 1 | Bromeliad | 3 | 166 | 5.43 | Masticate |  |  |
| ‘brom flowers ok_1’ | Divina | SAF | Sequence 1 | Bromeliad | 166 | 341 | 5.83 | Ingestive | Oral-Manual | Anterior |
| ‘brom flowers ok_1’ | Divina | SAF | Sequence 1 | Bromeliad | 341 | 367 | 0.87 | Masticate |  |  |
| ‘brom flowers ok_1’ | Divina | SAF | Sequence 1 | Bromeliad | 367 | 374 | 0.23 | Ingestive | Oral-Manual | Anterior |
| ‘brom flowers ok_1’ | Divina | SAF | Sequence 1 | Bromeliad | 374 | 415 | 1.37 | Masticate |  |  |
| ‘brom flowers ok_1’ | Divina | SAF | Sequence 1 | Bromeliad | 415 | 427 | 0.40 | Ingestive | Oral-Manual | Anterior |
| ‘brom flowers ok_1’ | Divina | SAF | Sequence 1 | Bromeliad | 427 | 459 | 1.07 | Masticate |  |  |
| ‘brom flowers ok_1’ | Divina | SAF | Sequence 1 | Bromeliad | 459 | 501 | 1.40 | Ingestive | Oral-Manual | Anterior |
| ‘brom flowers ok_1’ | Divina | SAF | Sequence 1 | Bromeliad | 501 | 530 | 0.97 | Masticate |  |  |
| ‘brom flowers ok_1’ | Divina | SAF | Sequence 1 | Bromeliad | 530 | 536 | 0.20 | Ingestive | Oral-Manual | Anterior |
| ‘brom flowers ok_1’ | Divina | SAF | Sequence 1 | Bromeliad | 536 | 553 | 0.57 | Masticate |  |  |
| ‘brom flowers ok_2’ | Divina | SAF | Sequence 1 | Bromeliad | 6 | 33 | 0.90 | Ingestive | Oral-Manual | Posterior |
| ‘brom flowers ok_2’ | Divina | SAF | Sequence 1 | Bromeliad | 147 | 174 | 0.90 | Ingestive | Oral-Manual | Posterior |
| ‘brom flowers ok_2’ | Divina | SAF | Sequence 1 | Bromeliad | 345 | 389 | 1.47 | Ingestive | Oral-Manual | Posterior |
| ‘brom flowers ok_2’ | Divina | SAF | Sequence 1 | Bromeliad | 389 | 450 | 2.03 | Masticate |  |  |
| ‘brom flowers ok_3’ | Divina | SAF | Sequence 1 | Bromeliad | 8 | 84 | 2.53 | Ingestive | Oral-Manual | Posterior |
| ‘brom flowers ok_3’ | Divina | SAF | Sequence 1 | Bromeliad | 146 | 181 | 1.17 | Ingestive | Oral-Manual | Anterior |
| ‘brom flowers ok_3’ | Divina | SAF | Sequence 1 | Bromeliad | 181 | 297 | 3.87 | Masticate |  |  |
| ‘brom flowers ok_3’ | Divina | SAF | Sequence 1 | Bromeliad | 297 | 340 | 1.43 | Ingestive | Oral-Manual | Anterior |
| ‘brom flowers ok_3’ | Divina | SAF | Sequence 1 | Bromeliad | 484 | 573 | 2.97 | Ingestive | Oral-Manual | Anterior |
| ‘brom flowers ok_3’ | Divina | SAF | Sequence 1 | Bromeliad | 573 | 673 | 3.33 | Masticate |  |  |
| ‘brom flowers ok_3’ | Divina | SAF | Sequence 1 | Bromeliad | 673 | 752 | 2.63 | Ingestive | Oral-Manual | Anterior |
| ‘brom flowers ok_3’ | Divina | SAF | Sequence 1 | fruit | 827 | 851 | 0.80 | Ingestive | Oral-Manual | Anterior |
| ‘brom flowers ok_3’ | Divina | SAF | Sequence 1 | fruit | 883 | 946 | 2.10 | Ingestive | Oral-Manual | Anterior |
| ‘brom flowers ok_3’ | Divina | SAF | Sequence 1 | fruit | 1029 | 1057 | 0.93 | Ingestive | Oral-Manual | Posterior |
| ‘brom flowers ok_3’ | Divina | SAF | Sequence 1 | fruit | 1258 | 1283 | 0.83 | Ingestive | Oral-Manual | Anterior |
| ‘brom flowers ok_3’ | Divina | SAF | Sequence 1 | fruit | 1491 | 1556 | 2.17 | Ingestive | Oral-Manual | Anterior |
| ‘brom flowers ok_3’ | Divina | SAF | Sequence 1 | fruit | 1678 | 1684 | 0.20 | Ingestive | Oral-Manual | Anterior |
| ‘brom flowers ok_3’ | Divina | SAF | Sequence 1 | fruit | 1684 | 1783 | 3.30 | Masticate |  |  |
| ‘brom flowers ok_3’ | Divina | SAF | Sequence 1 | fruit | 1783 | 1841 | 1.93 | Ingestive | Oral-Manual | Anterior |
| ‘brom flowers ok_3’ | Divina | SAF | Sequence 1 | fruit | 1841 | 1970 | 4.30 | Masticate |  |  |
| ‘brom flowers ok_3’ | Divina | SAF | Sequence 1 | fruit | 1970 | 1975 | 0.17 | Ingestive | Oral-Manual | Anterior |
| ‘brom flowers ok_3’ | Divina | SAF | Sequence 1 | fruit | 1975 | 2188 | 7.10 | Masticate |  |  |
| ‘brom flowers ok_3’ | Divina | SAF | Sequence 1 | fruit | 2188 | 2210 | 0.73 | Ingestive | Oral-Manual | Anterior |
| ‘brom flowers ok_3’ | Divina | SAF | Sequence 1 | fruit | 2241 | 2248 | 0.23 | Ingestive | Oral-Manual | Anterior |
| ‘brom flowers ok_3’ | Divina | SAF | Sequence 1 | fruit | 2293 | 2304 | 0.37 | Ingestive | Oral-Manual | Anterior |
| ‘brom flowers ok_3’ | Divina | SAF | Sequence 1 | fruit | 2304 | 2353 | 1.63 | Masticate |  |  |
| ‘brom flowers ok_3’ | Divina | SAF | Sequence 1 | fruit | 2353 | 2430 | 2.57 | Ingestive | Oral-Manual | Anterior |
| ‘brom flowers ok_3’ | Divina | SAF | Sequence 1 | fruit | 2430 | 2604 | 5.80 | Masticate |  |  |
| ‘brom flowers ok_3’ | Divina | SAF | Sequence 1 | fruit | 2604 | 2613 | 0.30 | Ingestive | Oral-Manual | Anterior |
| ‘brom flowers ok_3’ | Divina | SAF | Sequence 1 | fruit | 2613 | 2740 | 4.23 | Masticate |  |  |
| ‘brom flowers ok_3’ | Divina | SAF | Sequence 1 | fruit | 2740 | 2825 | 2.83 | Ingestive | Oral-Manual | Anterior |
| ‘brom flowers ok_3’ | Divina | SAF | Sequence 1 | fruit | 2825 | 3068 | 8.10 | Masticate |  |  |
| ‘brom flowers ok_3’ | Divina | SAF | Sequence 1 | fruit | 3068 | 3185 | 3.90 | Ingestive | Oral-Manual | Anterior |
| ‘bromedliad_extra good_1’ | Doree | AF | Sequence 1 | USO | 29 | 32 | 0.10 | Ingestive | Oral-Manual | Anterior |
| ‘bromedliad_extra good_1’ | Doree | AF | Sequence 1 | USO | 129 | 191 | 2.07 | Ingestive | Oral | Anterior |
| ‘bromedliad_extra good_1’ | Doree | AF | Sequence 1 | USO | 402 | 438 | 1.20 | Ingestive | Oral-Manual | Posterior |
| ‘bromedliad_extra good_1’ | Doree | AF | Sequence 1 | USO | 438 | 496 | 1.93 | Masticate |  |  |
| ‘bromedliad_extra good_2’ | Doree | AF | Sequence 1 | Bromeliad | 12 | 45 | 1.10 | Ingestive | Oral-Manual | Posterior |
| ‘bromedliad_extra good_2’ | Doree | AF | Sequence 1 | Bromeliad | 45 | 84 | 1.30 | Masticate |  |  |
| ‘bromedliad_extra good_2’ | Doree | AF | Sequence 1 | Bromeliad | 84 | 130 | 1.53 | Ingestive | Oral-Manual | Posterior |
| ‘bromedliad_extra good_2’ | Doree | AF | Sequence 1 | Bromeliad | 130 | 159 | 0.97 | Masticate |  |  |
| ‘bromedliad_extra good_2’ | Doree | AF | Sequence 1 | Bromeliad | 159 | 192 | 1.10 | Ingestive | Oral-Manual | Posterior |
| ‘bromedliad_extra good_2’ | Doree | AF | Sequence 1 | Bromeliad | 226 | 262 | 1.20 | Ingestive | Oral-Manual | Posterior |
| ‘bromedliad_extra good_2’ | Doree | AF | Sequence 1 | Bromeliad | 343 | 388 | 1.50 | Ingestive | Oral-Manual | Posterior |
| ‘bromedliad_extra good_2’ | Doree | AF | Sequence 1 | Bromeliad | 414 | 447 | 1.10 | Ingestive | Oral-Manual | Posterior |
| ‘bromedliad_extra good_2’ | Doree | AF | Sequence 1 | Bromeliad | 497 | 541 | 1.47 | Ingestive | Oral-Manual | Posterior |
| ‘bromedliad_extra good_3’ | Doree | AF | Sequence 1 | Bromeliad | 1 | 42 | 1.37 | Ingestive | Oral-Manual | Posterior |
| ‘bromedliad_extra good_3’ | Doree | AF | Sequence 1 | Bromeliad | 42 | 85 | 1.43 | Masticate |  |  |
| ‘bromedliad_extra good_3’ | Doree | AF | Sequence 1 | Bromeliad | 85 | 104 | 0.63 | Ingestive | Oral-Manual | Posterior |
| ‘bromedliad_extra good_3’ | Doree | AF | Sequence 1 | Bromeliad | 284 | 375 | 3.03 | Ingestive | Oral-Manual | Posterior |
| ‘bromedliad_extra good_3’ | Doree | AF | Sequence 1 | Bromeliad | 375 | 414 | 1.30 | Masticate |  |  |
| ‘bromedliad_extra good_3’ | Doree | AF | Sequence 1 | Bromeliad | 414 | 457 | 1.43 | Ingestive | Oral-Manual | Posterior |
| ‘bromedliad_extra good_3’ | Doree | AF | Sequence 1 | Bromeliad | 457 | 491 | 1.13 | Masticate |  |  |
| ‘bromedliad_extra good_3’ | Doree | AF | Sequence 1 | Bromeliad | 491 | 524 | 1.10 | Ingestive | Oral-Manual | Posterior |
| ‘bromedliad_extra good_3’ | Doree | AF | Sequence 1 | Bromeliad | 524 | 541 | 0.57 | Masticate |  |  |
| ‘bromedliad_extra good_3’ | Doree | AF | Sequence 1 | Bromeliad | 541 | 603 | 2.07 | Manual |  |  |
| ‘bromedliad_extra good_3’ | Doree | AF | Sequence 1 | Bromeliad | 603 | 650 | 1.57 | Ingestive | Oral-Manual | Posterior |
| ‘bromedliad_extra good_3’ | Doree | AF | Sequence 1 | Bromeliad | 719 | 753 | 1.13 | Ingestive | Oral-Manual | Posterior |
| ‘bromedliad_extra good_3’ | Doree | AF | Sequence 1 | Bromeliad | 767 | 816 | 1.63 | Ingestive | Oral-Manual | Posterior |
| ‘bromedliad_extra good_3’ | Doree | AF | Sequence 1 | Bromeliad | 901 | 905 | 0.13 | Ingestive | Oral-Manual | Posterior |
| ‘bromedliad_extra good_3’ | Doree | AF | Sequence 1 | Bromeliad | 955 | 985 | 1.00 | Ingestive | Oral-Manual | Posterior |
| ‘bromedliad_extra good_3’ | Doree | AF | Sequence 1 | Bromeliad | 1015 | 1053 | 1.27 | Ingestive | Oral-Manual | Posterior |
| ‘Coco_jatoba pod_1_1’ | Coco | AM | Sequence 1 | Pods | 79 | 93 | 0.47 | Ingestive | Oral-Manual | Anterior |
| ‘Coco_jatoba pod_1_1’ | Coco | AM | Sequence 1 | Pods | 116 | 149 | 1.10 | Ingestive | Oral-Manual | Anterior |
| ‘Coco_jatoba pod_1_1’ | Coco | AM | Sequence 1 | Pods | 220 | 236 | 0.53 | Ingestive | Oral-Manual | Anterior |
| ‘Coco_jatoba pod_1_1’ | Coco | AM | Sequence 1 | Pods | 276 | 329 | 1.77 | Ingestive | Oral-Manual | Anterior |
| ‘Coco_jatoba pod_1_1’ | Coco | AM | Sequence 1 | Pods | 365 | 390 | 0.83 | Ingestive | Oral-Manual | Anterior |
| ‘Coco_jatoba pod_1_1’ | Coco | AM | Sequence 1 | Pods | 409 | 420 | 0.37 | Ingestive | Oral-Manual | Anterior |
| ‘Coco_jatoba pod_1_1’ | Coco | AM | Sequence 1 | Pods | 646 | 653 | 0.23 | Ingestive | Oral-Manual | Anterior |
| ‘Coco_jatoba pod_1_1’ | Coco | AM | Sequence 1 | Pods | 682 | 717 | 1.17 | Ingestive | Oral-Manual | Anterior |
| ‘Coco_jatoba pod_1_1’ | Coco | AM | Sequence 1 | Pods | 717 | 753 | 1.20 | Ingestive | Oral-Manual | Anterior |
| ‘Coco_jatoba pod_1_1’ | Coco | AM | Sequence 1 | Pods | 753 | 777 | 0.80 | Ingestive | Oral-Manual | Anterior |
| ‘Coco_jatoba pod_1_1’ | Coco | AM | Sequence 1 | fruit | 979 | 995 | 0.53 | Ingestive | Oral-Manual | Posterior |
| ‘Coco_jatoba pod_1_1’ | Coco | AM | Sequence 1 | Pods | 1032 | 1040 | 0.27 | Ingestive | Oral-Manual | Anterior |
| ‘Coco_jatoba pod_1_1’ | Coco | AM | Sequence 1 | fruit | 1069 | 1092 | 0.77 | Ingestive | Oral-Manual | Posterior |
| ‘Coco_jatoba pod_1_1’ | Coco | AM | Sequence 1 | Pods | 1258 | 1276 | 0.60 | Ingestive | Oral-Manual | Anterior |
| ‘Coco_jatoba pod_1_1’ | Coco | AM | Sequence 1 | Pods | 1306 | 1330 | 0.80 | Ingestive | Oral-Manual | Anterior |
| ‘Coco_jatoba pod_1_1’ | Coco | AM | Sequence 1 | Pods | 1431 | 1497 | 2.20 | Ingestive | Oral-Manual | Anterior |
| ‘Coco_jatoba pod_1_1’ | Coco | AM | Sequence 1 | fruit | 1497 | 1543 | 1.53 | Masticate |  |  |
| ‘Coco_jatoba pod_1_1’ | Coco | AM | Sequence 1 | Pods | 1543 | 1561 | 0.60 | Ingestive | Oral-Manual | Anterior |
| ‘Coco_jatoba pod_1_1’ | Coco | AM | Sequence 1 | Pods | 1601 | 1639 | 1.27 | Ingestive | Oral-Manual | Anterior |
| ‘Coco_jatoba pod_1_1’ | Coco | AM | Sequence 1 | Pods | 1676 | 1690 | 0.47 | Ingestive | Oral-Manual | Anterior |
| ‘Coco_jatoba pod_1_1’ | Coco | AM | Sequence 1 | Pods | 1690 | 1718 | 0.93 | Ingestive | Oral-Manual | Anterior |
| ‘Coco_jatoba pod_1_1’ | Coco | AM | Sequence 1 | Pods | 1737 | 1789 | 1.73 | Ingestive | Oral-Manual | Anterior |
| ‘Coco_jatoba pod_1_1’ | Coco | AM | Sequence 1 | Pods | 1845 | 1881 | 1.20 | Ingestive | Oral-Manual | Anterior |
| ‘Coco_jatoba pod_1_1’ | Coco | AM | Sequence 1 | Pods | 1913 | 1922 | 0.30 | Ingestive | Oral-Manual | Anterior |
| ‘Coco_jatoba pod_1_2’ | Coco | AM | Sequence 1 | Pods | 21 | 45 | 0.80 | Ingestive | Oral-Manual | Anterior |
| ‘Coco_jatoba pod_1_2’ | Coco | AM | Sequence 1 | Pods | 66 | 96 | 1.00 | Ingestive | Oral-Manual | Anterior |
| ‘Coco_jatoba pod_1_2’ | Coco | AM | Sequence 1 | fruit | 96 | 136 | 1.33 | Manual |  |  |
| ‘Coco_jatoba pod_1_2’ | Coco | AM | Sequence 1 | Pods | 136 | 173 | 1.23 | Ingestive | Oral-Manual | Anterior |
| ‘Coco_jatoba pod_1_2’ | Coco | AM | Sequence 1 | fruit | 173 | 210 | 1.23 | Manual |  |  |
| ‘Coco_jatoba pod_1_2’ | Coco | AM | Sequence 1 | Pods | 210 | 245 | 1.17 | Ingestive | Oral-Manual | Anterior |
| ‘Coco_jatoba pod_1_2’ | Coco | AM | Sequence 1 | Pods | 257 | 382 | 4.17 | Ingestive | Oral-Manual | Anterior |
| ‘Coco_jatoba pod_1_2’ | Coco | AM | Sequence 1 | fruit | 382 | 415 | 1.10 | Masticate |  |  |
| ‘Coco_jatoba pod_1_2’ | Coco | AM | Sequence 1 | Pods | 415 | 448 | 1.10 | Ingestive | Oral-Manual | Anterior |
| ‘Coco_jatoba pod_1_2’ | Coco | AM | Sequence 1 | fruit | 448 | 492 | 1.47 | Manual |  |  |
| ‘Coco_jatoba pod_1_2’ | Coco | AM | Sequence 1 | Pods | 492 | 567 | 2.50 | Ingestive | Oral-Manual | Anterior |
| ‘Coco_jatoba pod_1_2’ | Coco | AM | Sequence 1 | Pods | 590 | 620 | 1.00 | Ingestive | Oral-Manual | Anterior |
| ‘Coco_jatoba pod_1_2’ | Coco | AM | Sequence 1 | Pods | 631 | 664 | 1.10 | Ingestive | Oral-Manual | Anterior |
| ‘Coco_jatoba pod_1_2’ | Coco | AM | Sequence 1 | fruit | 664 | 710 | 1.53 | Masticate |  |  |
| ‘Coco_jatoba pod_1_2’ | Coco | AM | Sequence 1 | Pods | 752 | 783 | 1.03 | Ingestive | Oral-Manual | Anterior |
| ‘Coco_jatoba pod_1_2’ | Coco | AM | Sequence 1 | Pods | 808 | 830 | 0.73 | Ingestive | Oral-Manual | Anterior |
| ‘Coco_jatoba pod_1_2’ | Coco | AM | Sequence 1 | Pods | 850 | 864 | 0.47 | Ingestive | Oral-Manual | Anterior |
| ‘Coco_jatoba pod_1_2’ | Coco | AM | Sequence 1 | Pods | 906 | 932 | 0.87 | Ingestive | Oral-Manual | Anterior |
| ‘complete tuber seq_1’ | Patricia | SAF | Sequence 1 | USO | 141 | 207 | 2.20 | Ingestive | Oral-Manual | Anterior |
| ‘complete tuber seq_1’ | Patricia | SAF | Sequence 1 | USO | 289 | 360 | 2.37 | Ingestive | Oral-Manual | Anterior |
| ‘complete tuber seq_1’ | Patricia | SAF | Sequence 1 | USO | 395 | 429 | 1.13 | Ingestive | Oral-Manual | Anterior |
| ‘complete tuber seq_1’ | Patricia | SAF | Sequence 1 | USO | 456 | 481 | 0.83 | Ingestive | Oral-Manual | Anterior |
| ‘complete tuber seq_1’ | Patricia | SAF | Sequence 1 | USO | 509 | 533 | 0.80 | Ingestive | Oral-Manual | Anterior |
| ‘complete tuber seq_1’ | Patricia | SAF | Sequence 1 | USO | 565 | 598 | 1.10 | Ingestive | Oral-Manual | Anterior |
| ‘complete tuber seq_1’ | Patricia | SAF | Sequence 1 | USO | 629 | 684 | 1.83 | Ingestive | Oral-Manual | Anterior |
| ‘complete tuber seq_1’ | Patricia | SAF | Sequence 1 | USO | 721 | 844 | 4.10 | Masticate |  |  |
| ‘complete tuber seq_1’ | Patricia | SAF | Sequence 1 | USO | 844 | 887 | 1.43 | Ingestive | Oral-Manual | Posterior |
| ‘complete tuber seq_1’ | Patricia | SAF | Sequence 1 | USO | 887 | 933 | 1.53 | Masticate |  |  |
| ‘complete tuber seq_1’ | Patricia | SAF | Sequence 1 | USO | 933 | 976 | 1.43 | Ingestive | Oral-Manual | Posterior |
| ‘complete tuber seq_1’ | Patricia | SAF | Sequence 1 | USO | 976 | 1274 | 9.93 | Masticate |  |  |
| ‘complete tuber seq_1’ | Patricia | SAF | Sequence 1 | USO | 1274 | 1317 | 1.43 | Ingestive | Oral-Manual | Posterior |
| ‘complete tuber seq_1’ | Patricia | SAF | Sequence 1 | USO | 1317 | 1371 | 1.80 | Masticate |  |  |
| ‘complete tuber seq_1’ | Patricia | SAF | Sequence 1 | USO | 1371 | 1421 | 1.67 | Ingestive | Oral-Manual | Posterior |
| ‘complete tuber seq_1’ | Patricia | SAF | Sequence 1 | USO | 1421 | 1517 | 3.20 | Masticate |  |  |
| ‘complete tuber seq_1’ | Patricia | SAF | Sequence 1 | USO | 1517 | 1604 | 2.90 | Ingestive | Oral-Manual | Posterior |
| ‘complete tuber seq_2’ | Patricia | SAF | Sequence 1 | USO | 31 | 79 | 1.60 | Ingestive | Oral-Manual | Posterior |
| ‘complete tuber seq_2’ | Patricia | SAF | Sequence 1 | USO | 114 | 202 | 2.93 | Ingestive | Oral-Manual | Posterior |
| ‘complete tuber seq_2’ | Patricia | SAF | Sequence 1 | USO | 202 | 229 | 0.90 | Masticate |  |  |
| ‘complete tuber seq_2’ | Patricia | SAF | Sequence 1 | USO | 229 | 346 | 3.90 | Ingestive | Oral-Manual | Posterior |
| ‘complete tuber seq_2’ | Patricia | SAF | Sequence 1 | USO | 346 | 392 | 1.53 | Masticate |  |  |
| ‘complete tuber seq_2’ | Patricia | SAF | Sequence 1 | USO | 392 | 436 | 1.47 | Ingestive | Oral-Manual | Anterior |
| ‘complete tuber seq_2’ | Patricia | SAF | Sequence 1 | USO | 436 | 514 | 2.60 | Ingestive | Oral-Manual | Posterior |
| ‘complete tuber seq_2’ | Patricia | SAF | Sequence 1 | USO | 514 | 536 | 0.73 | Ingestive | Oral-Manual | Posterior |
| ‘complete tuber seq_2’ | Patricia | SAF | Sequence 1 | USO | 536 | 579 | 1.43 | Masticate |  |  |
| ‘complete tuber seq_2’ | Patricia | SAF | Sequence 1 | USO | 579 | 598 | 0.63 | Ingestive | Oral-Manual | Anterior |
| ‘complete tuber seq_2’ | Patricia | SAF | Sequence 1 | USO | 598 | 621 | 0.77 | Ingestive | Oral-Manual | Posterior |
| ‘complete tuber seq_2’ | Patricia | SAF | Sequence 1 | USO | 639 | 662 | 0.77 | Ingestive | Oral-Manual | Posterior |
| ‘complete tuber seq_2’ | Patricia | SAF | Sequence 1 | USO | 662 | 726 | 2.13 | Masticate |  |  |
| ‘complete tuber seq_2’ | Patricia | SAF | Sequence 1 | USO | 726 | 805 | 2.63 | Ingestive | Oral-Manual | Posterior |
| ‘complete tuber seq_2’ | Patricia | SAF | Sequence 1 | USO | 805 | 855 | 1.67 | Masticate |  |  |
| ‘complete tuber seq_2’ | Patricia | SAF | Sequence 1 | USO | 855 | 883 | 0.93 | Ingestive | Oral-Manual | Anterior |
| ‘complete tuber seq_2’ | Patricia | SAF | Sequence 1 | USO | 883 | 916 | 1.10 | Masticate |  |  |
| ‘complete tuber seq_2’ | Patricia | SAF | Sequence 1 | USO | 916 | 941 | 0.83 | Ingestive | Oral-Manual | Anterior |
| ‘complete tuber seq_2’ | Patricia | SAF | Sequence 1 | USO | 941 | 998 | 1.90 | Ingestive | Oral-Manual | Posterior |
| ‘complete tuber seq_2’ | Patricia | SAF | Sequence 1 | USO | 998 | 1042 | 1.47 | Masticate |  |  |
| ‘complete tuber seq_2’ | Patricia | SAF | Sequence 1 | USO | 1042 | 1111 | 2.30 | Ingestive | Oral-Manual | Posterior |
| ‘complete tuber seq_2’ | Patricia | SAF | Sequence 1 | USO | 1111 | 1152 | 1.37 | Masticate |  |  |
| ‘eating summit’ | Pamonha | AF | Sequence 1 | Berries | 22 | 100 | 2.60 | Ingestive | Oral-Manual | Posterior |
| ‘eating summit’ | Pamonha | AF | Sequence 1 | Berries | 100 | 137 | 1.23 | Masticate |  |  |
| ‘eating summit’ | Pamonha | AF | Sequence 1 | Berries | 137 | 183 | 1.53 | Ingestive | Oral-Manual | Anterior |
| ‘eating summit’ | Pamonha | AF | Sequence 1 | Berries | 183 | 228 | 1.50 | Masticate |  |  |
| ‘eating summit’ | Pamonha | AF | Sequence 1 | Berries | 228 | 260 | 1.07 | Ingestive | Oral-Manual | Anterior |
| ‘eating summit’ | Pamonha | AF | Sequence 1 | Berries | 260 | 304 | 1.47 | Masticate |  |  |
| ‘eating summit’ | Pamonha | AF | Sequence 1 | Berries | 304 | 319 | 0.50 | Ingestive | Oral-Manual | Anterior |
| ‘eating summit’ | Pamonha | AF | Sequence 1 | Berries | 319 | 357 | 1.27 | Masticate |  |  |
| ‘eating summit’ | Pamonha | AF | Sequence 1 | Berries | 357 | 386 | 0.97 | Ingestive | Oral-Manual | Posterior |
| ‘eating summit’ | Pamonha | AF | Sequence 1 | Berries | 386 | 444 | 1.93 | Masticate |  |  |
| ‘eating summit’ | Pamonha | AF | Sequence 1 | Berries | 444 | 472 | 0.93 | Ingestive | Oral-Manual | Anterior |
| ‘eating summit’ | Pamonha | AF | Sequence 2 | Berries | 542 | 552 | 0.33 | Ingestive | Oral-Manual | Anterior |
| ‘eating summit’ | Pamonha | AF | Sequence 2 | Berries | 552 | 573 | 0.70 | Ingestive | Oral-Manual | Anterior |
| ‘eating summit’ | Pamonha | AF | Sequence 2 | Berries | 748 | 829 | 2.70 | Ingestive | Oral-Manual | Posterior |
| ‘eating summit’ | Pamonha | AF | Sequence 2 | Berries | 848 | 864 | 0.53 | Ingestive | Oral-Manual | Posterior |
| ‘eating summit’ | Pamonha | AF | Sequence 2 | Berries | 864 | 913 | 1.63 | Masticate |  |  |
| ‘eating summit’ | Pamonha | AF | Sequence 2 | Berries | 924 | 937 | 0.43 | Ingestive | Oral-Manual | Posterior |
| ‘eating summit’ | Pamonha | AF | Sequence 2 | Berries | 937 | 978 | 1.37 | Masticate |  |  |
| ‘eating summit’ | Pamonha | AF | Sequence 2 | Berries | 978 | 1057 | 2.63 | Ingestive | Oral-Manual | Anterior |
| ‘eating summit’ | Pamonha | AF | Sequence 2 | Berries | 1057 | 1092 | 1.17 | Masticate |  |  |
| ‘eating summit’ | Pamonha | AF | Sequence 2 | Berries | 1092 | 1125 | 1.10 | Ingestive | Oral-Manual | Anterior |
| ‘eating summit’ | Pamonha | AF | Sequence 2 | Berries | 1125 | 1153 | 0.93 | Masticate |  |  |
| ‘eating summit’ | Pamonha | AF | Sequence 2 | Berries | 1153 | 1160 | 0.23 | Ingestive | Oral-Manual | Anterior |
| ‘eating summit’ | Pamonha | AF | Sequence 2 | Berries | 1179 | 1194 | 0.50 | Ingestive | Oral-Manual | Anterior |
| ‘eating summit’ | Pamonha | AF | Sequence 2 | Berries | 1236 | 1239 | 0.10 |  |  |  |
| ‘eating summit’ | Pamonha | AF | Sequence 3 | Berries | 1351 | 1353 | 0.07 | Ingestive | Oral-Manual | Anterior |
| ‘eating summit’ | Pamonha | AF | Sequence 4 | Berries | 1472 | 1527 | 1.83 | Ingestive | Oral-Manual | Posterior |
| ‘family got the tuber’ | Patricia | SAF | Sequence 1 | USO | 323 | 367 | 1.47 | Ingestive | Oral-Manual | Anterior |
| ‘family got the tuber’ | Patricia | SAF | Sequence 1 | USO | 422 | 491 | 2.30 | Ingestive | Oral-Manual | Anterior |
| ‘family got the tuber’ | Patricia | SAF | Sequence 1 | USO | 491 | 514 | 0.77 | Ingestive | Oral-Manual | Anterior |
| ‘family got the tuber’ | Patricia | SAF | Sequence 1 | USO | 514 | 657 | 4.77 | Ingestive | Oral-Manual | Anterior |
| ‘family got the tuber’ | Patricia | SAF | Sequence 1 | USO | 705 | 724 | 0.63 | Ingestive | Oral-Manual | Anterior |
| ‘family got the tuber’ | Patricia | SAF | Sequence 1 | USO | 772 | 793 | 0.70 | Ingestive | Oral-Manual | Anterior |
| ‘family got the tuber’ | Patricia | SAF | Sequence 1 | USO | 845 | 1129 | 9.47 | Ingestive | Oral-Manual | Posterior |
| ‘family got the tuber’ | Patricia | SAF | Sequence 1 | USO | 1162 | 1246 | 2.80 | Ingestive | Oral-Manual | Posterior |
| ‘family got the tuber’ | Patricia | SAF | Sequence 1 | USO | 1269 | 1310 | 1.37 | Ingestive | Oral-Manual | Posterior |
| ‘good m de boi’ | Presente | SAM | Sequence 1 | fruit | 85 | 102 | 0.57 | Ingestive | Oral | Anterior |
| ‘good m de boi’ | Presente | SAM | Sequence 1 | fruit | 130 | 158 | 0.93 | Ingestive | Oral-Manual | Anterior |
| ‘good m de boi’ | Presente | SAM | Sequence 1 | fruit | 228 | 232 | 0.13 | Ingestive | Oral-Manual | Anterior |
| ‘good m de boi’ | Presente | SAM | Sequence 1 | fruit | 323 | 367 | 1.47 | Ingestive | Oral-Manual | Anterior |
| ‘good m de boi’ | Presente | SAM | Sequence 1 | fruit | 394 | 432 | 1.27 | Ingestive | Oral-Manual | Anterior |
| ‘good m de boi’ | Presente | SAM | Sequence 1 | fruit | 507 | 557 | 1.67 | Ingestive | Oral-Manual | Anterior |
| ‘good m de boi’ | Presente | SAM | Sequence 1 | fruit | 557 | 598 | 1.37 | Manual |  |  |
| ‘good m de boi’ | Presente | SAM | Sequence 1 | fruit | 598 | 667 | 2.30 | Ingestive | Oral-Manual | Anterior |
| ‘good m de boi’ | Presente | SAM | Sequence 1 | fruit | 707 | 725 | 0.60 | Ingestive | Oral-Manual | Anterior |
| ‘good m de boi’ | Presente | SAM | Sequence 1 | fruit | 767 | 805 | 1.27 | Ingestive | Oral-Manual | Anterior |
| ‘good m de boi’ | Presente | SAM | Sequence 1 | fruit | 805 | 881 | 2.53 | Manual |  |  |
| ‘good m de boi’ | Presente | SAM | Sequence 1 | fruit | 881 | 930 | 1.63 | Ingestive | Oral-Manual | Anterior |
| ‘good m de boi’ | Presente | SAM | Sequence 1 | fruit | 955 | 967 | 0.40 | Ingestive | Oral-Manual | Anterior |
| ‘good m de boi’ | Presente | SAM | Sequence 1 | fruit | 967 | 1092 | 4.17 | Manual |  |  |
| ‘good m de boi’ | Presente | SAM | Sequence 1 | fruit | 1092 | 1121 | 0.97 | Ingestive | Oral-Manual | Anterior |
| ‘good m de boi’ | Presente | SAM | Sequence 1 | fruit | 1145 | 1178 | 1.10 | Ingestive | Oral-Manual | Anterior |
| ‘good m de boi’ | Presente | SAM | Sequence 1 | fruit | 1178 | 1310 | 4.40 | Masticate |  |  |
| ‘good m de boi’ | Presente | SAM | Sequence 1 | fruit | 1310 | 1334 | 0.80 | Ingestive | Oral-Manual | Anterior |
| ‘good m de boi’ | Presente | SAM | Sequence 1 | fruit | 1334 | 1375 | 1.37 | Masticate |  |  |
| ‘good m de boi’ | Presente | SAM | Sequence 1 | fruit | 1375 | 1385 | 0.33 | Ingestive | Oral-Manual | Anterior |
| ‘good m de boi’ | Presente | SAM | Sequence 1 | fruit | 1416 | 1445 | 0.97 | Ingestive | Oral-Manual | Anterior |
| ‘good m de boi’ | Presente | SAM | Sequence 1 | fruit | 1466 | 1481 | 0.50 | Ingestive | Oral-Manual | Anterior |
| ‘good m de boi’ | Presente | SAM | Sequence 1 | fruit | 1481 | 1531 | 1.67 | Masticate |  |  |
| ‘good m de boi’ | Presente | SAM | Sequence 1 | fruit | 1531 | 1578 | 1.57 | Ingestive | Oral-Manual | Anterior |
| ‘good m de boi’ | Presente | SAM | Sequence 1 | fruit | 1598 | 1605 | 0.23 | Ingestive | Oral-Manual | Anterior |
| ‘good m de boi’ | Presente | SAM | Sequence 1 | fruit | 1641 | 1643 | 0.07 | Ingestive | Oral-Manual | Anterior |
| ‘good m de boi’ | Presente | SAM | Sequence 1 | fruit | 1700 | 1709 | 0.30 | Ingestive | Oral-Manual | Anterior |
| ‘good m de boi’ | Presente | SAM | Sequence 1 | fruit | 1762 | 1779 | 0.57 | Ingestive | Oral-Manual | Anterior |
| ‘good m de boi’ | Presente | SAM | Sequence 1 | fruit | 1810 | 1868 | 1.93 | Ingestive | Oral-Manual | Anterior |
| ‘good m de boi’ | Presente | SAM | Sequence 1 | fruit | 1895 | 1927 | 1.07 | Ingestive | Oral-Manual | Anterior |
| ‘good m de boi’ | Presente | SAM | Sequence 1 | fruit | 1927 | 1978 | 1.70 | Manual |  |  |
| ‘good m de boi’ | Presente | SAM | Sequence 1 | fruit | 1978 | 1980 | 0.07 | Ingestive | Oral-Manual | Anterior |
| ‘good m de boi’ | Presente | SAM | Sequence 1 | fruit | 1980 | 2039 | 1.97 | Masticate |  |  |
| ‘good m de boi’ | Presente | SAM | Sequence 1 | fruit | 2039 | 2079 | 1.33 | Ingestive | Oral-Manual | Anterior |
| ‘‘i’m yellow podding’ 2’ | Donzela | SAF | Sequence 1 | Pods | 95 | 111 | 0.53 | Ingestive | Oral | Anterior |
| ‘‘i’m yellow podding’ 2’ | Donzela | SAF | Sequence 1 | Pods | 546 | 553 | 0.23 | Ingestive | Oral | Anterior |
| ‘‘i’m yellow podding’ 2’ | Donzela | SAF | Sequence 1 | Pods | 641 | 665 | 0.80 | Ingestive | Oral-Manual | Anterior |
| ‘‘i’m yellow podding’ 2’ | Donzela | SAF | Sequence 1 | Pods | 1157 | 1188 | 1.03 | Ingestive | Oral-Manual | Anterior |
| ‘‘i’m yellow podding’ 2’ | Donzela | SAF | Sequence 1 | Pods | 1250 | 1299 | 1.63 | Ingestive | Oral-Manual | Anterior |
| ‘‘i’m yellow podding’ 2’ | Donzela | SAF | Sequence 1 | Pods | 1299 | 1753 | 15.13 | Masticate |  |  |
| ‘i’m yellow podding’ | Donzela | SAF | Sequence 1 | Pods | 1 | 84 | 2.77 | Manual |  |  |
| ‘i’m yellow podding’ | Donzela | SAF | Sequence 1 | Pods | 84 | 89 | 0.17 | Ingestive | Oral-Manual | Anterior |
| ‘i’m yellow podding’ | Donzela | SAF | Sequence 1 | Pods | 89 | 385 | 9.87 | Masticate |  |  |
| ‘i’m yellow podding’ | Donzela | SAF | Sequence 1 | Pods | 385 | 388 | 0.10 | Ingestive | Oral-Manual | Anterior |
| ‘i’m yellow podding’ | Donzela | SAF | Sequence 1 | Pods | 388 | 691 | 10.10 | Masticate |  |  |
| ‘i’m yellow podding’ | Donzela | SAF | Sequence 1 | Pods | 691 | 695 | 0.13 | Ingestive | Oral-Manual | Anterior |
| ‘jatoba complete tucum seq 4’ | Jatoba | AM | Sequence 1 | tucum | 230 | 322 | 3.07 | Manual |  |  |
| ‘jatoba complete tucum seq 4’ | Jatoba | AM | Sequence 1 | tucum | 502 | 549 | 1.57 | Manual |  |  |
| ‘jatoba complete tucum seq 4’ | Jatoba | AM | Sequence 1 | tucum | 817 | 827 | 0.33 | Ingestive | Oral-Manual | Anterior |
| ‘jatoba complete tucum seq 4’ | Jatoba | AM | Sequence 1 | tucum | 858 | 1000 | 4.73 | Ingestive | Oral-Manual | Anterior |
| ‘jatoba complete tucum seq 4’ | Jatoba | AM | Sequence 1 | tucum | 1000 | 1322 | 10.73 | Masticate |  |  |
| ‘jatoba complete tucum seq 4’ | Jatoba | AM | Sequence 1 | tucum | 1366 | 1368 | 0.07 | Ingestive | Oral-Manual | Anterior |
| ‘jatoba complete tucum seq 4’ | Jatoba | AM | Sequence 1 | tucum | 1389 | 1428 | 1.30 | Ingestive | Oral-Manual | Anterior |
| ‘jatoba complete tucum seq 4’ | Jatoba | AM | Sequence 1 | tucum | 1764 | 1963 | 6.63 | Ingestive | Oral-Manual | Posterior |
| ‘jatoba complete tucum seq 4’ | Jatoba | AM | Sequence 1 | tucum | 1963 | 2307 | 11.47 | Masticate |  |  |
| ‘jatoba complete tucum seq 4’ | Jatoba | AM | Sequence 1 | tucum | 2307 | 2394 | 2.90 | Ingestive | Oral-Manual | Anterior |
| ‘jatoba complete tucum seq 4’ | Jatoba | AM | Sequence 1 | tucum | 2410 | 2472 | 2.07 | Ingestive | Oral-Manual | Anterior |
| ‘jatoba complete tucum seq 4’ | Jatoba | AM | Sequence 1 | tucum | 2472 | 2498 | 0.87 | Masticate |  |  |
| ‘jatoba complete tucum seq 4’ | Jatoba | AM | Sequence 1 | tucum | 2498 | 2530 | 1.07 | Ingestive | Oral-Manual | Anterior |
| ‘jatoba complete tucum seq 4’ | Jatoba | AM | Sequence 1 | tucum | 2530 | 2631 | 3.37 | Masticate |  |  |
| ‘jatoba complete tucum seq 4’ | Jatoba | AM | Sequence 1 | tucum | 2631 | 2678 | 1.57 | Ingestive | Oral-Manual | Anterior |
| ‘jatoba complete tucum seq 4’ | Jatoba | AM | Sequence 1 | tucum | 2678 | 2706 | 0.93 | Masticate |  |  |
| ‘jatoba complete tucum seq 4’ | Jatoba | AM | Sequence 1 | tucum | 2706 | 2710 | 0.13 | Ingestive | Oral-Manual | Anterior |
| ‘jatoba complete tucum seq 4’ | Jatoba | AM | Sequence 1 | tucum | 2710 | 2769 | 1.97 | Masticate |  |  |
| ‘jatoba complete tucum seq 4’ | Jatoba | AM | Sequence 1 | tucum | 2769 | 2806 | 1.23 | Ingestive | Oral-Manual | Anterior |
| ‘jatoba complete tucum seq 4’ | Jatoba | AM | Sequence 1 | tucum | 2806 | 3216 | 13.67 | Masticate |  |  |
| ‘jatoba complete tucum seq 5’ | Jatoba | AM | Sequence 1 | tucum | 261 | 325 | 2.13 | Manual |  |  |
| ‘jatoba complete tucum seq 5’ | Jatoba | AM | Sequence 1 | tucum | 516 | 613 | 3.23 | Manual |  |  |
| ‘jatoba complete tucum seq 5’ | Jatoba | AM | Sequence 1 | tucum | 613 | 648 | 1.17 | Ingestive | Oral-Manual | Anterior |
| ‘jatoba complete tucum seq 5’ | Jatoba | AM | Sequence 1 | tucum | 648 | 730 | 2.73 | Masticate |  |  |
| ‘jatoba complete tucum seq 5’ | Jatoba | AM | Sequence 1 | tucum | 730 | 790 | 2.00 | Ingestive | Oral-Manual | Anterior |
| ‘jatoba complete tucum seq 5’ | Jatoba | AM | Sequence 1 | tucum | 790 | 1067 | 9.23 | Masticate |  |  |
| ‘jatoba complete tucum seq 5’ | Jatoba | AM | Sequence 1 | tucum | 1067 | 1075 | 0.27 | Ingestive | Oral-Manual | Anterior |
| ‘jatoba complete tucum seq 5’ | Jatoba | AM | Sequence 1 | tucum | 1094 | 1160 | 2.20 | Ingestive | Oral-Manual | Anterior |
| ‘jatoba complete tucum seq 5’ | Jatoba | AM | Sequence 1 | tucum | 1160 | 1194 | 1.13 | Masticate |  |  |
| ‘jatoba complete tucum seq 5’ | Jatoba | AM | Sequence 1 | tucum | 1194 | 1225 | 1.03 | Ingestive | Oral-Manual | Anterior |
| ‘jatoba complete tucum seq 5’ | Jatoba | AM | Sequence 1 | tucum | 1225 | 1502 | 9.23 | Masticate |  |  |
| ‘jatoba complete tucum seq 5’ | Jatoba | AM | Sequence 1 | tucum | 1502 | 1568 | 2.20 | Ingestive | Oral-Manual | Anterior |
| ‘jatoba complete tucum seq 5’ | Jatoba | AM | Sequence 1 | tucum | 1586 | 1590 | 0.13 | Ingestive | Oral-Manual | Anterior |
| ‘jatoba complete tucum seq 5’ | Jatoba | AM | Sequence 1 | tucum | 1590 | 1628 | 1.27 | Masticate |  |  |
| ‘jatoba complete tucum seq 5’ | Jatoba | AM | Sequence 1 | tucum | 1628 | 1787 | 5.30 | Ingestive | Oral-Manual | Anterior |
| ‘jatoba complete tucum seq 5’ | Jatoba | AM | Sequence 1 | tucum | 1787 | 1852 | 2.17 | Masticate |  |  |
| ‘jatoba complete tucum seq 5’ | Jatoba | AM | Sequence 1 | tucum | 1852 | 1892 | 1.33 | Ingestive | Oral-Manual | Anterior |
| ‘jatoba complete tucum seq 5’ | Jatoba | AM | Sequence 1 | tucum | 1892 | 1916 | 0.80 | Masticate |  |  |
| ‘jatoba complete tucum seq 5’ | Jatoba | AM | Sequence 1 | tucum | 1916 | 1943 | 0.90 | Ingestive | Oral-Manual | Anterior |
| ‘jatoba complete tucum seq 5’ | Jatoba | AM | Sequence 1 | tucum | 1943 | 1985 | 1.40 | Masticate |  |  |
| ‘jatoba complete tucum seq 5’ | Jatoba | AM | Sequence 1 | tucum | 1985 | 2032 | 1.57 | Ingestive | Oral-Manual | Posterior |
| ‘jatoba complete tucum seq 5’ | Jatoba | AM | Sequence 1 | tucum | 2113 | 2235 | 4.07 | Ingestive | Oral-Manual | Anterior |
| ‘jatoba complete tucum seq 5’ | Jatoba | AM | Sequence 1 | tucum | 2235 | 2357 | 4.07 | Masticate |  |  |
| ‘jatoba complete tucum seq 5’ | Jatoba | AM | Sequence 1 | tucum | 2357 | 2371 | 0.47 | Ingestive | Oral-Manual | Anterior |
| ‘jatoba complete tucum seq 5’ | Jatoba | AM | Sequence 1 | tucum | 2395 | 2436 | 1.37 | Ingestive | Oral-Manual | Anterior |
| ‘jatoba complete tucum seq 5’ | Jatoba | AM | Sequence 1 | tucum | 2436 | 2648 | 7.07 | Masticate |  |  |
| ‘jatoba complete tucum seq 5’ | Jatoba | AM | Sequence 1 | tucum | 2648 | 2678 | 1.00 | Ingestive | Oral-Manual | Anterior |
| ‘jatoba complete tucum seq 5’ | Jatoba | AM | Sequence 1 | tucum | 2678 | 2980 | 10.07 | Masticate |  |  |
| ‘jatoba cracks and rejects’ | Jatoba | AM | Sequence 1 | Piaçava | 367 | 613 | 8.20 | Manual |  |  |
| ‘jatoba cracks and rejects’ | Jatoba | AM | Sequence 1 | Piaçava | 715 | 789 | 2.47 | Manual |  |  |
| ‘jatoba nut scratching’ | Jatoba | AM | Sequence 1 | Piaçava | 164 | 438 | 9.13 | Manual |  |  |
| ‘jatoba nut scratching’ | Jatoba | AM | Sequence 1 | Piaçava | 203 | 430 | 7.57 | Manual |  |  |
| ‘jatoba on anvil’ | Jatoba | AM | Sequence 1 | Piaçava | 385 | 935 | 18.33 | Manual |  |  |
| ‘jatoba Piaçava seq 1_1’ | Jatoba | AM | Sequence 1 | Piaçava | 45 | 88 | 1.43 | Ingestive | Oral-Manual | Anterior |
| ‘jatoba Piaçava seq 1_1’ | Jatoba | AM | Sequence 1 | Piaçava | 122 | 160 | 1.27 | Ingestive | Oral-Manual | Anterior |
| ‘jatoba Piaçava seq 1_1’ | Jatoba | AM | Sequence 1 | Piaçava | 402 | 465 | 2.10 | Manual |  |  |
| ‘jatoba Piaçava seq 1_2’ | Jatoba | AM | Sequence 1 | Piaçava | 1 | 34 | 1.10 | Ingestive | Oral-Manual | Anterior |
| ‘jatoba Piaçava seq 1_2’ | Jatoba | AM | Sequence 1 | Piaçava | 34 | 346 | 10.40 | Manual |  |  |
| ‘jatoba Piaçava seq 1_2’ | Jatoba | AM | Sequence 1 | Piaçava | 346 | 386 | 1.33 | Ingestive | Oral-Manual | Anterior |
| ‘jatoba Piaçava seq 1_2’ | Jatoba | AM | Sequence 1 | Piaçava | 408 | 414 | 0.20 | Ingestive | Oral-Manual | Anterior |
| ‘jatoba Piaçava seq 1_2’ | Jatoba | AM | Sequence 1 | Piaçava | 414 | 583 | 5.63 | Masticate |  |  |
| ‘jatoba Piaçava seq 1_2’ | Jatoba | AM | Sequence 1 | Piaçava | 583 | 596 | 0.43 | Ingestive | Oral-Manual | Anterior |
| ‘jatoba Piaçava seq 1_2’ | Jatoba | AM | Sequence 1 | Piaçava | 596 | 727 | 4.37 | Manual |  |  |
| ‘jatoba Piaçava seq 1_2’ | Jatoba | AM | Sequence 1 | Piaçava | 727 | 750 | 0.77 | Ingestive | Oral-Manual | Anterior |
| ‘jatoba Piaçava seq 1_2’ | Jatoba | AM | Sequence 1 | Piaçava | 750 | 803 | 1.77 | Ingestive | Oral-Manual | Posterior |
| ‘jatoba Piaçava seq 1_2’ | Jatoba | AM | Sequence 1 | Piaçava | 803 | 913 | 3.67 | Masticate |  |  |
| ‘jatoba Piaçava seq 1_2’ | Jatoba | AM | Sequence 1 | Piaçava | 913 | 915 | 0.07 | Ingestive | Oral-Manual | Anterior |
| ‘jatoba Piaçava seq 1_2’ | Jatoba | AM | Sequence 1 | Piaçava | 915 | 1177 | 8.73 | Masticate |  |  |
| ‘jatoba Piaçava seq 1_2’ | Jatoba | AM | Sequence 1 | Piaçava | 1177 | 1211 | 1.13 | Ingestive | Oral-Manual | Anterior |
| ‘jatoba Piaçava seq 1_2’ | Jatoba | AM | Sequence 1 | Piaçava | 1211 | 1300 | 2.97 | Masticate |  |  |
| ‘jatoba Piaçava seq 1_2’ | Jatoba | AM | Sequence 1 | Piaçava | 1300 | 1316 | 0.53 | Ingestive | Oral-Manual | Anterior |
| ‘jatoba Piaçava seq 1_2’ | Jatoba | AM | Sequence 1 | Piaçava | 1338 | 1359 | 0.70 | Ingestive | Oral-Manual | Anterior |
| ‘jatoba Piaçava seq 1_2’ | Jatoba | AM | Sequence 1 | Piaçava | 1380 | 1388 | 0.27 | Ingestive | Oral-Manual | Anterior |
| ‘jatoba Piaçava seq 1_2’ | Jatoba | AM | Sequence 1 | Piaçava | 1405 | 1418 | 0.43 | Ingestive | Oral-Manual | Anterior |
| ‘jatoba Piaçava seq 1_2’ | Jatoba | AM | Sequence 1 | Piaçava | 1487 | 1524 | 1.23 | Ingestive | Oral-Manual | Posterior |
| ‘jatoba Piaçava seq 1_2’ | Jatoba | AM | Sequence 1 | Piaçava | 1524 | 1857 | 11.10 | Masticate |  |  |
| ‘jatoba Piaçava seq 1_2’ | Jatoba | AM | Sequence 1 | Piaçava | 1857 | 1889 | 1.07 | Ingestive | Oral-Manual | Posterior |
| ‘jatoba Piaçava seq 1_2’ | Jatoba | AM | Sequence 1 | Piaçava | 1889 | 2275 | 12.87 | Masticate |  |  |
| ‘jatoba Piaçava seq 1_2’ | Jatoba | AM | Sequence 1 | Piaçava | 2275 | 2385 | 3.67 | Ingestive | Oral-Manual | Anterior |
| ‘jatoba Piaçava seq 1_2’ | Jatoba | AM | Sequence 1 | Piaçava | 2390 | 2435 | 1.50 | Ingestive | Oral-Manual | Anterior |
| ‘jatoba Piaçava seq 1_2’ | Jatoba | AM | Sequence 1 | Piaçava | 2435 | 2493 | 1.93 | Masticate |  |  |
| ‘jatoba Piaçava seq 1_2’ | Jatoba | AM | Sequence 1 | Piaçava | 2493 | 2519 | 0.87 | Ingestive | Oral-Manual | Posterior |
| ‘jatoba Piaçava seq 1_2’ | Jatoba | AM | Sequence 1 | Piaçava | 2519 | 2911 | 13.07 | Masticate |  |  |
| ‘jatoba Piaçava seq 1_2’ | Jatoba | AM | Sequence 1 | Piaçava | 2911 | 2915 | 0.13 | Ingestive | Oral-Manual | Anterior |
| ‘jatoba Piaçava seq 1_2’ | Jatoba | AM | Sequence 1 | Piaçava | 2915 | 3313 | 13.27 | Masticate |  |  |
| ‘jatoba Piaçava seq 1_2’ | Jatoba | AM | Sequence 1 | Piaçava | 3424 | 3445 | 0.70 | Ingestive | Oral-Manual | Anterior |
| ‘jatoba Piaçava seq 1_2’ | Jatoba | AM | Sequence 1 | Piaçava | 3476 | 3477 | 0.03 | Ingestive | Oral-Manual | Anterior |
| ‘jatoba Piaçava seq 1_2’ | Jatoba | AM | Sequence 1 | Piaçava | 3517 | 3531 | 0.47 | Ingestive | Oral-Manual | Anterior |
| ‘jatoba Piaçava seq 1_2’ | Jatoba | AM | Sequence 1 | Piaçava | 3531 | 3552 | 0.70 | Ingestive | Oral-Manual | Anterior |
| ‘jatoba Piaçava seq 1_2’ | Jatoba | AM | Sequence 1 | Piaçava | 3552 | 3571 | 0.63 | Masticate |  |  |
| ‘jatoba Piaçava seq 1_2’ | Jatoba | AM | Sequence 1 | Piaçava | 3571 | 3582 | 0.37 | Ingestive | Oral-Manual | Anterior |
| ‘jatoba Piaçava seq 1_2’ | Jatoba | AM | Sequence 1 | Piaçava | 3878 | 3939 | 2.03 | Ingestive | Oral-Manual | Anterior |
| ‘jatoba Piaçava seq 1_2’ | Jatoba | AM | Sequence 1 | Piaçava | 3972 | 4054 | 2.73 | Ingestive | Oral-Manual | Anterior |
| ‘jatoba Piaçava seq 1_2’ | Jatoba | AM | Sequence 1 | Piaçava | 4099 | 4133 | 1.13 | Ingestive | Oral-Manual | Anterior |
| ‘jatoba Piaçava seq 1_2’ | Jatoba | AM | Sequence 1 | Piaçava | 4133 | 4224 | 3.03 | Masticate |  |  |
| ‘jatoba Piaçava seq 1_2’ | Jatoba | AM | Sequence 1 | Piaçava | 4224 | 4290 | 2.20 | Ingestive | Oral-Manual | Anterior |
| ‘jatoba Piaçava seq 1_2’ | Jatoba | AM | Sequence 1 | Piaçava | 4427 | 4493 | 2.20 | Ingestive | Oral-Manual | Anterior |
| ‘jatoba Piaçava seq 1_2’ | Jatoba | AM | Sequence 1 | Piaçava | 4571 | 4634 | 2.10 | Ingestive | Oral-Manual | Anterior |
| ‘jatoba Piaçava seq 1_2’ | Jatoba | AM | Sequence 1 | Piaçava | 4896 | 5085 | 6.30 | Ingestive | Oral-Manual | Anterior |
| ‘jatoba Piaçava seq 1_2’ | Jatoba | AM | Sequence 1 | Piaçava | 5112 | 5120 | 0.27 | Ingestive | Oral-Manual | Anterior |
| ‘jatoba Piaçava seq 1_2’ | Jatoba | AM | Sequence 1 | Piaçava | 5150 | 5217 | 2.23 | Ingestive | Oral-Manual | Anterior |
| ‘juvie fruit nice_1’ | Donzela | SAF | Sequence 1 | fruit | 25 | 68 | 1.43 | Ingestive | Oral-Manual | Anterior |
| ‘juvie fruit nice_1’ | Donzela | SAF | Sequence 1 | fruit | 68 | 118 | 1.67 | Masticate |  |  |
| ‘juvie fruit nice_1’ | Donzela | SAF | Sequence 1 | fruit | 203 | 225 | 0.73 | Ingestive | Oral-Manual | Anterior |
| ‘juvie fruit nice_1’ | Donzela | SAF | Sequence 1 | fruit | 254 | 274 | 0.67 | Ingestive | Oral-Manual | Anterior |
| ‘juvie fruit nice_1’ | Donzela | SAF | Sequence 1 | fruit | 274 | 317 | 1.43 | Masticate |  |  |
| ‘juvie fruit nice_1’ | Donzela | SAF | Sequence 1 | fruit | 317 | 339 | 0.73 | Ingestive | Oral-Manual | Anterior |
| ‘juvie fruit nice_1’ | Donzela | SAF | Sequence 1 | fruit | 339 | 363 | 0.80 | Ingestive | Oral-Manual | Anterior |
| ‘juvie fruit nice_1’ | Donzela | SAF | Sequence 1 | fruit | 399 | 441 | 1.40 | Ingestive | Oral-Manual | Anterior |
| ‘juvie fruit nice_1’ | Donzela | SAF | Sequence 1 | fruit | 498 | 550 | 1.73 | Ingestive | Oral-Manual | Anterior |
| ‘juvie fruit nice_1’ | Donzela | SAF | Sequence 1 | fruit | 550 | 652 | 3.40 | Masticate |  |  |
| ‘juvie fruit nice_1’ | Donzela | SAF | Sequence 1 | fruit | 670 | 732 | 2.07 | Ingestive | Oral-Manual | Anterior |
| ‘juvie fruit nice_1’ | Donzela | SAF | Sequence 1 | fruit | 732 | 778 | 1.53 | Masticate |  |  |
| ‘juvie fruit nice_1’ | Donzela | SAF | Sequence 1 | fruit | 778 | 817 | 1.30 | Ingestive | Oral-Manual | Anterior |
| ‘juvie fruit nice_2’ | Donzela | SAF | Sequence 1 | fruit | 1 | 47 | 1.53 | Ingestive | Oral-Manual | Anterior |
| ‘juvie fruit nice_2’ | Donzela | SAF | Sequence 1 | fruit | 79 | 110 | 1.03 | Ingestive | Oral-Manual | Anterior |
| ‘juvie fruit nice_2’ | Donzela | SAF | Sequence 1 | fruit | 134 | 168 | 1.13 | Ingestive | Oral-Manual | Anterior |
| ‘juvie fruit nice_2’ | Donzela | SAF | Sequence 1 | fruit | 249 | 328 | 2.63 | Ingestive | Oral-Manual | Anterior |
| ‘juvie fruit nice_2’ | Donzela | SAF | Sequence 1 | fruit | 435 | 443 | 0.27 | Ingestive | Oral-Manual | Anterior |
| ‘juvie fruit nice_2’ | Donzela | SAF | Sequence 1 | fruit | 510 | 553 | 1.43 | Ingestive | Oral-Manual | Anterior |
| ‘juvie fruit nice_2’ | Donzela | SAF | Sequence 1 | fruit | 748 | 765 | 0.57 | Ingestive | Oral-Manual | Anterior |
| ‘juvie fruit nice_2’ | Donzela | SAF | Sequence 1 | fruit | 908 | 923 | 0.50 | Ingestive | Oral-Manual | Anterior |
| ‘juvie fruit nice_2’ | Donzela | SAF | Sequence 1 | fruit | 1018 | 1033 | 0.50 | Ingestive | Oral-Manual | Anterior |
| ‘m de boi more’ | Piassava | AF | Sequence 1 | fruit | 15 | 17 | 0.07 | Ingestive | Oral-Manual | Anterior |
| ‘m de boi more’ | Piassava | AF | Sequence 1 | fruit | 97 | 101 | 0.13 | Ingestive | Oral-Manual | Anterior |
| ‘m de boi more’ | Piassava | AF | Sequence 1 | fruit | 101 | 150 | 1.63 | Masticate |  |  |
| ‘m de boi more’ | Piassava | AF | Sequence 1 | fruit | 150 | 173 | 0.77 | Ingestive | Oral-Manual | Anterior |
| ‘m de boi more’ | Piassava | AF | Sequence 1 | fruit | 200 | 237 | 1.23 | Ingestive | Oral-Manual | Anterior |
| ‘m de boi more’ | Piassava | AF | Sequence 1 | fruit | 690 | 750 | 2.00 | Ingestive | Oral-Manual | Anterior |
| ‘m de boi more’ | Piassava | AF | Sequence 1 | fruit | 810 | 840 | 1.00 | Ingestive | Oral-Manual | Anterior |
| ‘m de boi more’ | Piassava | AF | Sequence 1 | fruit | 1020 | 1029 | 0.30 | Ingestive | Oral-Manual | Anterior |
| ‘m de boi nahce_1’ | Pacoca | AF | Sequence 1 | fruit | 17 | 31 | 0.47 | Ingestive | Oral-Manual | Anterior |
| ‘m de boi nahce_1’ | Pacoca | AF | Sequence 1 | fruit | 31 | 60 | 0.97 | Masticate |  |  |
| ‘m de boi nahce_1’ | Pacoca | AF | Sequence 1 | fruit | 60 | 79 | 0.63 | Ingestive | Oral-Manual | Anterior |
| ‘m de boi nahce_1’ | Pacoca | AF | Sequence 1 | fruit | 79 | 119 | 1.33 | Masticate |  |  |
| ‘m de boi nahce_1’ | Pacoca | AF | Sequence 1 | fruit | 119 | 134 | 0.50 | Ingestive | Oral-Manual | Anterior |
| ‘m de boi nahce_1’ | Pacoca | AF | Sequence 1 | fruit | 156 | 170 | 0.47 | Ingestive | Oral-Manual | Anterior |
| ‘m de boi nahce_2’ | Pacoca | AF | Sequence 1 | fruit | 1 | 10 | 0.30 | Ingestive | Oral-Manual | Anterior |
| ‘m de boi nahce_2’ | Pacoca | AF | Sequence 1 | fruit | 27 | 35 | 0.27 | Ingestive | Oral-Manual | Anterior |
| ‘m de boi nahce_3’ | Pacoca | AF | Sequence 1 | fruit | 1 | 20 | 0.63 | Ingestive | Oral-Manual | Anterior |
| ‘m de boi nahce_3’ | Pacoca | AF | Sequence 1 | fruit | 49 | 72 | 0.77 | Ingestive | Oral-Manual | Anterior |
| ‘m de boi nahce_3’ | Pacoca | AF | Sequence 1 | fruit | 89 | 99 | 0.33 | Ingestive | Oral-Manual | Anterior |
| ‘m de boi nahce_3’ | Pacoca | AF | Sequence 1 | fruit | 123 | 150 | 0.90 | Ingestive | Oral-Manual | Anterior |
| ‘m de boi nahce_4’ | Pacoca | AF | Sequence 1 | fruit | 1 | 83 | 2.73 | Ingestive | Oral-Manual | Anterior |
| ‘m de boi nahce_4’ | Pacoca | AF | Sequence 1 | fruit | 83 | 129 | 1.53 | Masticate |  |  |
| ‘m de boi nahce_4’ | Pacoca | AF | Sequence 1 | fruit | 129 | 151 | 0.73 | Ingestive | Oral-Manual | Anterior |
| ‘m de boi nahce_4’ | Pacoca | AF | Sequence 1 | fruit | 200 | 235 | 1.17 | Ingestive | Oral-Manual | Posterior |
| ‘m de boi nice seqs’ | Coco | AM | Sequence 1 | fruit | 57 | 155 | 3.27 | Ingestive | Oral-Manual | Anterior |
| ‘m de boi nice seqs’ | Coco | AM | Sequence 1 | fruit | 181 | 255 | 2.47 | Ingestive | Oral-Manual | Anterior |
| ‘m de boi nice seqs’ | Coco | AM | Sequence 1 | fruit | 255 | 258 | 0.10 | Ingestive | Oral-Manual | Anterior |
| ‘m de boi nice seqs’ | Coco | AM | Sequence 1 | fruit | 258 | 300 | 1.40 | Manual |  |  |
| ‘m de boi nice seqs’ | Coco | AM | Sequence 1 | fruit | 300 | 362 | 2.07 | Ingestive | Oral-Manual | Anterior |
| ‘m de boi nice seqs’ | Coco | AM | Sequence 1 | fruit | 362 | 401 | 1.30 | Manual |  |  |
| ‘m de boi nice seqs’ | Coco | AM | Sequence 1 | fruit | 401 | 452 | 1.70 | Ingestive | Oral-Manual | Anterior |
| ‘m de boi nice seqs’ | Coco | AM | Sequence 1 | fruit | 487 | 506 | 0.63 | Ingestive | Oral-Manual | Anterior |
| ‘m de boi nice seqs’ | Coco | AM | Sequence 1 | fruit | 506 | 577 | 2.37 | Manual |  |  |
| ‘m de boi nice seqs’ | Coco | AM | Sequence 1 | fruit | 577 | 583 | 0.20 | Ingestive | Oral-Manual | Anterior |
| ‘m de boi nice seqs’ | Coco | AM | Sequence 1 | fruit | 583 | 651 | 2.27 | Manual |  |  |
| ‘m de boi nice seqs’ | Coco | AM | Sequence 1 | fruit | 651 | 769 | 3.93 | Ingestive | Oral-Manual | Anterior |
| ‘m de boi nice seqs’ | Coco | AM | Sequence 1 | fruit | 824 | 880 | 1.87 | Ingestive | Oral-Manual | Anterior |
| ‘m de boi nice seqs’ | Coco | AM | Sequence 1 | fruit | 903 | 951 | 1.60 | Ingestive | Oral-Manual | Anterior |
| ‘m de boi nice seqs’ | Coco | AM | Sequence 1 | fruit | 994 | 1028 | 1.13 | Ingestive | Oral-Manual | Anterior |
| ‘m de boi nice seqs’ | Coco | AM | Sequence 1 | fruit | 1028 | 1064 | 1.20 | Masticate |  |  |
| ‘m de boi nice seqs’ | Coco | AM | Sequence 1 | fruit | 1064 | 1089 | 0.83 | Ingestive | Oral-Manual | Anterior |
| ‘m de boi nice seqs’ | Coco | AM | Sequence 1 | fruit | 1089 | 1162 | 2.43 | Masticate |  |  |
| ‘m de boi nice seqs’ | Coco | AM | Sequence 1 | fruit | 1162 | 1186 | 0.80 | Ingestive | Oral-Manual | Anterior |
| ‘m de boi nice seqs’ | Coco | AM | Sequence 1 | fruit | 1186 | 1249 | 2.10 | Masticate |  |  |
| ‘m de boi nice seqs’ | Coco | AM | Sequence 1 | fruit | 1249 | 1277 | 0.93 | Ingestive | Oral-Manual | Anterior |
| ‘m de boi nice’ | Coco | AM | Sequence 1 | fruit | 1 | 8 | 0.23 | Ingestive | Oral-Manual | Anterior |
| ‘m de boi nice’ | Coco | AM | Sequence 1 | fruit | 8 | 26 | 0.60 | Masticate |  |  |
| ‘m de boi nice’ | Coco | AM | Sequence 1 | fruit | 26 | 64 | 1.27 | Ingestive | Oral-Manual | Anterior |
| ‘m de boi nice’ | Coco | AM | Sequence 1 | fruit | 64 | 115 | 1.70 | Masticate |  |  |
| ‘m de boi nice’ | Coco | AM | Sequence 1 | fruit | 115 | 180 | 2.17 | Ingestive | Oral-Manual | Anterior |
| ‘m de boi nice’ | Coco | AM | Sequence 1 | fruit | 180 | 247 | 2.23 | Masticate |  |  |
| ‘m de boi nice’ | Coco | AM | Sequence 1 | fruit | 247 | 313 | 2.20 | Ingestive | Oral-Manual | Anterior |
| ‘m de boi nice’ | Coco | AM | Sequence 1 | fruit | 313 | 333 | 0.67 | Masticate |  |  |
| ‘m de boi nice’ | Coco | AM | Sequence 1 | fruit | 333 | 358 | 0.83 | Ingestive | Oral-Manual | Anterior |
| ‘m de boi nice’ | Coco | AM | Sequence 1 | fruit | 358 | 386 | 0.93 | Masticate |  |  |
| ‘m de boi nice’ | Coco | AM | Sequence 1 | fruit | 386 | 394 | 0.27 |  |  |  |
| ‘m de boi nice’ | Coco | AM | Sequence 1 | fruit | 421 | 458 | 1.23 | Ingestive | Oral-Manual | Anterior |
| ‘m de boi nice’ | Coco | AM | Sequence 1 | fruit | 458 | 523 | 2.17 | Masticate |  |  |
| ‘m de boi nice’ | Coco | AM | Sequence 1 | fruit | 523 | 586 | 2.10 | Ingestive | Oral-Manual | Anterior |
| ‘m de boi nice’ | Coco | AM | Sequence 1 | fruit | 586 | 615 | 0.97 | Masticate |  |  |
| ‘m de boi nice’ | Coco | AM | Sequence 1 | fruit | 615 | 629 | 0.47 | Ingestive | Oral-Manual | Anterior |
| ‘m de boi nice’ | Coco | AM | Sequence 1 | fruit | 629 | 692 | 2.10 | Masticate |  |  |
| ‘m de boi nice’ | Coco | AM | Sequence 1 | fruit | 692 | 702 | 0.33 | Ingestive | Oral-Manual | Anterior |
| ‘m de boi nice’ | Coco | AM | Sequence 1 | fruit | 702 | 747 | 1.50 | Masticate |  |  |
| ‘m de boi nice’ | Coco | AM | Sequence 1 | fruit | 747 | 779 | 1.07 | Ingestive | Oral-Manual | Anterior |
| ‘m de boi nice’ | Coco | AM | Sequence 1 | fruit | 779 | 808 | 0.97 | Masticate |  |  |
| ‘m de boi nice’ | Coco | AM | Sequence 1 | fruit | 808 | 847 | 1.30 | Ingestive | Oral-Manual | Anterior |
| ‘m de boi nice’ | Coco | AM | Sequence 1 | fruit | 861 | 906 | 1.50 | Ingestive | Oral-Manual | Anterior |
| ‘m de boi nice’ | Coco | AM | Sequence 1 | fruit | 1203 | 1237 | 1.13 | Ingestive | Oral-Manual | Anterior |
| ‘m de boi nice’ | Coco | AM | Sequence 1 | fruit | 1237 | 1263 | 0.87 | Masticate |  |  |
| ‘m de boi nice’ | Coco | AM | Sequence 1 | fruit | 1263 | 1278 | 0.50 | Ingestive | Oral-Manual | Anterior |
| ‘m de boi nice’ | Coco | AM | Sequence 1 | fruit | 1332 | 1334 | 0.07 | Ingestive | Oral-Manual | Anterior |
| ‘m de boi nice’ | Coco | AM | Sequence 1 | fruit | 1334 | 1351 | 0.57 | Masticate |  |  |
| ‘m de boi nice’ | Coco | AM | Sequence 1 | fruit | 1351 | 1394 | 1.43 | Ingestive | Oral-Manual | Anterior |
| ‘m de boi seq’ | Presente | SAM | Sequence 1 | fruit | 3 | 88 | 2.83 | Ingestive | Oral-Manual | Anterior |
| ‘m de boi seq’ | Presente | SAM | Sequence 1 | fruit | 88 | 222 | 4.47 | Masticate |  |  |
| ‘m de boi seq’ | Presente | SAM | Sequence 1 | fruit | 222 | 227 | 0.17 | Ingestive | Oral-Manual | Anterior |
| ‘m de boi seq’ | Presente | SAM | Sequence 1 | fruit | 227 | 256 | 0.97 | Masticate |  |  |
| ‘m de boi seq’ | Presente | SAM | Sequence 1 | fruit | 256 | 331 | 2.50 | Ingestive | Oral-Manual | Anterior |
| ‘m de boi seq’ | Presente | SAM | Sequence 1 | fruit | 331 | 361 | 1.00 | Masticate |  |  |
| ‘m de boi seq’ | Presente | SAM | Sequence 1 | fruit | 361 | 399 | 1.27 | Ingestive | Oral-Manual | Anterior |
| ‘m de boi seq’ | Presente | SAM | Sequence 1 | fruit | 399 | 455 | 1.87 | Masticate |  |  |
| ‘m de boi seq’ | Presente | SAM | Sequence 1 | fruit | 455 | 485 | 1.00 | Ingestive | Oral-Manual | Anterior |
| ‘m de boi seq’ | Presente | SAM | Sequence 1 | fruit | 485 | 501 | 0.53 |  |  |  |
| ‘m de boi seq’ | Presente | SAM | Sequence 1 | fruit | 514 | 534 | 0.67 | Ingestive | Oral-Manual | Anterior |
| ‘m de boi seq’ | Presente | SAM | Sequence 1 | fruit | 534 | 623 | 2.97 | Masticate |  |  |
| ‘m de boi seq’ | Presente | SAM | Sequence 1 | fruit | 623 | 638 | 0.50 | Ingestive | Oral-Manual | Anterior |
| ‘m de boi seq’ | Presente | SAM | Sequence 1 | fruit | 638 | 682 | 1.47 | Masticate |  |  |
| ‘m de boi seq’ | Presente | SAM | Sequence 1 | fruit | 710 | 733 | 0.77 | Ingestive | Oral-Manual | Anterior |
| ‘m de boi seq’ | Presente | SAM | Sequence 1 | fruit | 733 | 847 | 3.80 | Masticate |  |  |
| ‘m de boi seq’ | Presente | SAM | Sequence 1 | fruit | 847 | 865 | 0.60 | Ingestive | Oral-Manual | Anterior |
| ‘m de boi seq’ | Presente | SAM | Sequence 1 | fruit | 865 | 894 | 0.97 | Masticate |  |  |
| ‘m de boi seq’ | Presente | SAM | Sequence 2 | fruit | 974 | 1037 | 2.10 | Ingestive | Oral-Manual | Anterior |
| ‘m de boi seq’ | Presente | SAM | Sequence 2 | fruit | 1065 | 1098 | 1.10 | Ingestive | Oral-Manual | Anterior |
| ‘m de boi seq’ | Presente | SAM | Sequence 2 | fruit | 1131 | 1163 | 1.07 | Ingestive | Oral-Manual | Anterior |
| ‘m de boi seq’ | Presente | SAM | Sequence 2 | fruit | 1183 | 1220 | 1.23 | Ingestive | Oral-Manual | Anterior |
| ‘m de boi seq’ | Presente | SAM | Sequence 2 | fruit | 1220 | 1249 | 0.97 | Manual |  |  |
| ‘m de boi seq’ | Presente | SAM | Sequence 2 | fruit | 1249 | 1275 | 0.87 | Ingestive | Oral-Manual | Anterior |
| ‘m de boi seq’ | Presente | SAM | Sequence 2 | fruit | 1341 | 1378 | 1.23 | Ingestive | Oral-Manual | Anterior |
| ‘m de boi seq’ | Presente | SAM | Sequence 2 | fruit | 1407 | 1440 | 1.10 | Ingestive | Oral-Manual | Anterior |
| ‘m de boi seq’ | Presente | SAM | Sequence 2 | fruit | 1440 | 1500 | 2.00 | Masticate |  |  |
| ‘m de boi seq’ | Presente | SAM | Sequence 2 | fruit | 1500 | 1539 | 1.30 | Ingestive | Oral-Manual | Anterior |
| ‘m de boi seq’ | Presente | SAM | Sequence 2 | fruit | 1539 | 1556 | 0.57 | Masticate |  |  |
| ‘m de boi seq’ | Presente | SAM | Sequence 2 | fruit | 1556 | 1573 | 0.57 | Ingestive | Oral-Manual | Anterior |
| ‘m de boi seq’ | Presente | SAM | Sequence 2 | fruit | 1573 | 1606 | 1.10 | Masticate |  |  |
| ‘m de boi seq’ | Presente | SAM | Sequence 2 | fruit | 1606 | 1635 | 0.97 | Ingestive | Oral-Manual | Anterior |
| ‘m de boi seq’ | Presente | SAM | Sequence 2 | fruit | 1635 | 1726 | 3.03 | Masticate |  |  |
| ‘m de boi sum momo’ | Pamonha | AF | Sequence 1 | fruit | 1 | 23 | 0.73 | Ingestive | Oral-Manual | Anterior |
| ‘m de boi sum momo’ | Pamonha | AF | Sequence 1 | fruit | 23 | 61 | 1.27 | Masticate |  |  |
| ‘m de boi sum momo’ | Pamonha | AF | Sequence 1 | fruit | 61 | 76 | 0.50 | Ingestive | Oral-Manual | Anterior |
| ‘m de boi sum momo’ | Pamonha | AF | Sequence 1 | fruit | 109 | 114 | 0.17 | Ingestive | Oral-Manual | Anterior |
| ‘m de boi sum momo’ | Pamonha | AF | Sequence 1 | fruit | 114 | 159 | 1.50 | Masticate |  |  |
| ‘m de boi sum momo’ | Pamonha | AF | Sequence 1 | fruit | 159 | 175 | 0.53 | Ingestive | Oral-Manual | Anterior |
| ‘m de boi sum momo’ | Pamonha | AF | Sequence 1 | fruit | 175 | 243 | 2.27 | Masticate |  |  |
| ‘m de boi sum momo’ | Pamonha | AF | Sequence 1 | fruit | 243 | 265 | 0.73 | Ingestive | Oral-Manual | Posterior |
| ‘m de boi sum momo’ | Pamonha | AF | Sequence 1 | fruit | 265 | 308 | 1.43 | Masticate |  |  |
| ‘m de boi sum momo’ | Pamonha | AF | Sequence 1 | fruit | 308 | 329 | 0.70 | Ingestive | Oral-Manual | Anterior |
| ‘m de boi sum momo’ | Pamonha | AF | Sequence 1 | fruit | 350 | 355 | 0.17 | Ingestive | Oral-Manual | Anterior |
| ‘m de boi’ | Piassava | AF | Sequence 1 | fruit | 35 | 75 | 1.33 | Ingestive | Oral | Anterior |
| ‘m de boi’ | Piassava | AF | Sequence 1 | fruit | 198 | 237 | 1.30 | Ingestive | Oral-Manual | Anterior |
| ‘m de boi’ | Piassava | AF | Sequence 1 | fruit | 237 | 275 | 1.27 | Manual |  |  |
| ‘m de boi’ | Piassava | AF | Sequence 1 | fruit | 275 | 296 | 0.70 | Ingestive | Oral-Manual | Anterior |
| ‘marm de boi_1’ | Donzela | SAF | Sequence 1 | fruit | 10 | 30 | 0.67 | Ingestive | Oral-Manual | Anterior |
| ‘marm de boi_1’ | Donzela | SAF | Sequence 1 | fruit | 30 | 107 | 2.57 | Masticate |  |  |
| ‘marm de boi_1’ | Donzela | SAF | Sequence 1 | fruit | 107 | 131 | 0.80 | Ingestive | Oral-Manual | Anterior |
| ‘marm de boi_1’ | Donzela | SAF | Sequence 1 | fruit | 131 | 153 | 0.73 | Masticate |  |  |
| ‘marm de boi_1’ | Donzela | SAF | Sequence 1 | fruit | 153 | 160 | 0.23 | Ingestive | Oral-Manual | Anterior |
| ‘marm de boi_1’ | Donzela | SAF | Sequence 1 | fruit | 177 | 187 | 0.33 | Ingestive | Oral-Manual | Anterior |
| ‘marm de boi_1’ | Donzela | SAF | Sequence 1 | fruit | 187 | 214 | 0.90 | Masticate |  |  |
| ‘marm de boi_1’ | Donzela | SAF | Sequence 1 | fruit | 214 | 265 | 1.70 | Ingestive | Oral-Manual | Anterior |
| ‘marm de boi_1’ | Donzela | SAF | Sequence 1 | fruit | 265 | 338 | 2.43 | Masticate |  |  |
| ‘marm de boi_2’ | Donzela | SAF | Sequence 1 | fruit | 51 | 96 | 1.50 | Ingestive | Oral-Manual | Anterior |
| ‘marm de boi_2’ | Donzela | SAF | Sequence 1 | fruit | 96 | 138 | 1.40 | Manual |  |  |
| ‘marm de boi_2’ | Donzela | SAF | Sequence 1 | fruit | 138 | 177 | 1.30 | Ingestive | Oral-Manual | Anterior |
| ‘marm de boi_2’ | Donzela | SAF | Sequence 1 | fruit | 203 | 228 | 0.83 | Ingestive | Oral-Manual | Anterior |
| ‘marm de boi_2’ | Donzela | SAF | Sequence 1 | fruit | 256 | 273 | 0.57 | Ingestive | Oral-Manual | Anterior |
| ‘marm de boi_2’ | Donzela | SAF | Sequence 1 | fruit | 366 | 391 | 0.83 | Ingestive | Oral-Manual | Anterior |
| ‘marm de boi_2’ | Donzela | SAF | Sequence 1 | fruit | 455 | 466 | 0.37 | Ingestive | Oral-Manual | Anterior |
| ‘monk marmalatta’ | Pacoca | AF | Sequence 1 | fruit | 379 | 424 | 1.50 | Ingestive | Oral-Manual | Anterior |
| ‘monk marmalatta’ | Pacoca | AF | Sequence 1 | fruit | 460 | 464 | 0.13 | Ingestive | Oral-Manual | Anterior |
| ‘monk marmalatta’ | Pacoca | AF | Sequence 1 | fruit | 517 | 564 | 1.57 | Ingestive | Oral-Manual | Posterior |
| ‘monk marmalatta’ | Pacoca | AF | Sequence 1 | fruit | 591 | 623 | 1.07 | Ingestive | Oral-Manual | Posterior |
| ‘monk marmalatta’ | Pacoca | AF | Sequence 1 | fruit | 623 | 725 | 3.40 | Ingestive | Oral-Manual | Anterior |
| ‘monk marmalatta’ | Pacoca | AF | Sequence 1 | fruit | 739 | 796 | 1.90 | Ingestive | Oral-Manual | Anterior |
| ‘monk marmalatta’ | Pacoca | AF | Sequence 1 | fruit | 840 | 889 | 1.63 | Ingestive | Oral-Manual | Anterior |
| ‘monk marmalatta’ | Pacoca | AF | Sequence 1 | fruit | 943 | 967 | 0.80 | Ingestive | Oral-Manual | Anterior |
| ‘monk marmalatta’ | Pacoca | AF | Sequence 1 | fruit | 1004 | 1047 | 1.43 | Ingestive | Oral-Manual | Anterior |
| ‘monk marmalatta’ | Pacoca | AF | Sequence 1 | fruit | 1109 | 1134 | 0.83 | Ingestive | Oral-Manual | Anterior |
| ‘monk marmalatta’ | Pacoca | AF | Sequence 1 | fruit | 1228 | 1252 | 0.80 | Ingestive | Oral-Manual | Anterior |
| ‘monk marmalatta’ | Pacoca | AF | Sequence 1 | fruit | 1275 | 1294 | 0.63 | Ingestive | Oral-Manual | Posterior |
| ‘monk marmalatta’ | Pacoca | AF | Sequence 1 | fruit | 1294 | 1375 | 2.70 | Masticate |  |  |
| ‘monk marmalatta’ | Pacoca | AF | Sequence 1 | fruit | 1375 | 1391 | 0.53 | Ingestive | Oral-Manual | Anterior |
| ‘monk marmalatta’ | Pacoca | AF | Sequence 1 | fruit | 1391 | 1478 | 2.90 | Masticate |  |  |
| ‘monkey twigging’ | Pamonha | AF | Sequence 1 | insects | 1 | 11 | 0.33 | Ingestive | Oral | Anterior |
| ‘monkey twigging’ | Pamonha | AF | Sequence 1 | insects | 78 | 87 | 0.30 | Ingestive | Oral | Anterior |
| ‘monkey twigging’ | Pamonha | AF | Sequence 1 | insects | 109 | 110 | 0.03 | Ingestive | Oral | Anterior |
| ‘monkey twigging’ | Pamonha | AF | Sequence 1 | insects | 136 | 163 | 0.90 | Ingestive | Oral | Anterior |
| ‘monkey twigging’ | Pamonha | AF | Sequence 1 | insects | 196 | 223 | 0.90 | Ingestive | Oral | Anterior |
| ‘monkey twigging’ | Pamonha | AF | Sequence 1 | insects | 242 | 265 | 0.77 |  |  |  |
| ‘monkey twigging’ | Pamonha | AF | Sequence 1 | insects | 347 | 379 | 1.07 | Ingestive | Oral | Anterior |
| ‘monkey twigging’ | Pamonha | AF | Sequence 1 | insects | 405 | 411 | 0.20 |  |  |  |
| ‘monkey twigging’ | Pamonha | AF | Sequence 1 | insects | 433 | 437 | 0.13 |  |  |  |
| ‘monkey twigging’ | Pamonha | AF | Sequence 1 | insects | 444 | 447 | 0.10 |  |  |  |
| ‘monkey twigging’ | Pamonha | AF | Sequence 1 | insects | 473 | 519 | 1.53 | Ingestive | Oral | Anterior |
| ‘monkey twigging’ | Pamonha | AF | Sequence 1 | insects | 532 | 546 | 0.47 | Ingestive | Oral | Anterior |
| ‘monkey twigging’ | Pamonha | AF | Sequence 1 | insects | 546 | 606 | 2.00 | Masticate |  |  |
| ‘monkey twigging’ | Pamonha | AF | Sequence 1 | insects | 606 | 618 | 0.40 | Ingestive | Oral-Manual | Anterior |
| ‘monkey twigging’ | Pamonha | AF | Sequence 1 | insects | 618 | 653 | 1.17 | Masticate |  |  |
| ‘monkey twigging’ | Pamonha | AF | Sequence 1 | insects | 653 | 661 | 0.27 | Ingestive | Oral-Manual | Anterior |
| ‘monkey twigging’ | Pamonha | AF | Sequence 1 | insects | 661 | 724 | 2.10 | Masticate |  |  |
| ‘monkey twigging’ | Pamonha | AF | Sequence 1 | insects | 724 | 761 | 1.23 | Ingestive | Oral | Anterior |
| ‘monkey twigging’ | Pamonha | AF | Sequence 1 | insects | 801 | 839 | 1.27 | Ingestive | Oral | Anterior |
| ‘monkey twigging’ | Pamonha | AF | Sequence 1 | insects | 865 | 885 | 0.67 | Ingestive | Oral | Anterior |
| ‘monkey twigging’ | Pamonha | AF | Sequence 1 | insects | 918 | 939 | 0.70 |  |  |  |
| ‘monkey twigging’ | Pamonha | AF | Sequence 1 | insects | 962 | 972 | 0.33 |  |  |  |
| ‘monkey twigging’ | Pamonha | AF | Sequence 1 | insects | 1005 | 1014 | 0.30 |  |  |  |
| ‘monkey twigging’ | Pamonha | AF | Sequence 1 | insects | 1073 | 1079 | 0.20 |  |  |  |
| ‘monkey twigging’ | Pamonha | AF | Sequence 1 | insects | 1099 | 1126 | 0.90 | Ingestive | Oral | Anterior |
| ‘monkey twigging’ | Pamonha | AF | Sequence 1 | insects | 1160 | 1163 | 0.10 |  |  |  |
| ‘monkey twigging’ | Pamonha | AF | Sequence 1 | insects | 1179 | 1198 | 0.63 | Ingestive | Oral | Anterior |
| ‘monkey twigging’ | Pamonha | AF | Sequence 1 | insects | 1214 | 1217 | 0.10 |  |  |  |
| ‘monkey twigging’ | Pamonha | AF | Sequence 1 | insects | 1230 | 1286 | 1.87 | Ingestive | Oral | Anterior |
| ‘monkey twigging’ | Pamonha | AF | Sequence 1 | insects | 1301 | 1321 | 0.67 | Ingestive | Oral | Anterior |
| ‘monkey twigging’ | Pamonha | AF | Sequence 1 | insects | 1340 | 1361 | 0.70 |  |  |  |
| ‘monkey twigging’ | Pamonha | AF | Sequence 1 | insects | 1380 | 1386 | 0.20 |  |  |  |
| ‘nice juvie feeding’ | Pacoca | AF | Sequence 1 | fruit | 38 | 42 | 0.13 | Ingestive | Oral-Manual | Anterior |
| ‘nice juvie feeding’ | Pacoca | AF | Sequence 1 | fruit | 67 | 104 | 1.23 | Ingestive | Oral-Manual | Anterior |
| ‘nice juvie feeding’ | Pacoca | AF | Sequence 1 | fruit | 117 | 186 | 2.30 | Ingestive | Oral-Manual | Anterior |
| ‘nice juvie feeding’ | Pacoca | AF | Sequence 1 | fruit | 186 | 246 | 2.00 | Masticate |  |  |
| ‘nice juvie feeding’ | Pacoca | AF | Sequence 1 | fruit | 246 | 251 | 0.17 | Ingestive | Oral-Manual | Anterior |
| ‘nice juvie feeding’ | Pacoca | AF | Sequence 1 | fruit | 251 | 298 | 1.57 | Masticate |  |  |
| ‘nice juvie feeding’ | Pacoca | AF | Sequence 1 | fruit | 298 | 331 | 1.10 | Ingestive | Oral-Manual | Anterior |
| ‘nice juvie feeding’ | Pacoca | AF | Sequence 1 | fruit | 331 | 355 | 0.80 | Masticate |  |  |
| ‘nice juvie feeding’ | Pacoca | AF | Sequence 1 | fruit | 355 | 388 | 1.10 | Ingestive | Oral-Manual | Anterior |
| ‘nice juvie feeding’ | Pacoca | AF | Sequence 1 | fruit | 388 | 413 | 0.83 | Masticate |  |  |
| ‘nice juvie feeding’ | Pacoca | AF | Sequence 1 | fruit | 413 | 470 | 1.90 | Ingestive | Oral-Manual | Anterior |
| ‘nice juvie feeding’ | Pacoca | AF | Sequence 1 | fruit | 470 | 512 | 1.40 | Masticate |  |  |
| ‘nice juvie feeding’ | Pacoca | AF | Sequence 1 | fruit | 512 | 515 | 0.10 | Ingestive | Oral-Manual | Anterior |
| ‘nice juvie feeding’ | Pacoca | AF | Sequence 1 | fruit | 515 | 563 | 1.60 | Masticate |  |  |
| ‘nice juvie feeding’ | Pacoca | AF | Sequence 1 | fruit | 563 | 589 | 0.87 | Manual |  |  |
| ‘nice juvie feeding’ | Pacoca | AF | Sequence 1 | fruit | 589 | 630 | 1.37 | Masticate |  |  |
| ‘nice juvie feeding’ | Pacoca | AF | Sequence 1 | fruit | 630 | 707 | 2.57 | Ingestive | Oral-Manual | Anterior |
| ‘nice juvie feeding’ | Pacoca | AF | Sequence 1 | fruit | 707 | 743 | 1.20 | Masticate |  |  |
| ‘nice juvie feeding’ | Pacoca | AF | Sequence 1 | fruit | 743 | 774 | 1.03 | Ingestive | Oral-Manual | Anterior |
| ‘nice juvie feeding’ | Pacoca | AF | Sequence 1 | fruit | 774 | 809 | 1.17 | Masticate |  |  |
| ‘nice juvie feeding’ | Pacoca | AF | Sequence 1 | fruit | 809 | 817 | 0.27 | Ingestive | Oral-Manual | Anterior |
| ‘nice juvie feeding’ | Pacoca | AF | Sequence 1 | fruit | 817 | 863 | 1.53 | Masticate |  |  |
| ‘nice juvie feeding’ | Pacoca | AF | Sequence 1 | fruit | 863 | 903 | 1.33 | Ingestive | Oral | Anterior |
| ‘nice juvie feeding’ | Pacoca | AF | Sequence 1 | fruit | 958 | 983 | 0.83 | Ingestive | Oral | Anterior |
| ‘nice juvie feeding’ | Pacoca | AF | Sequence 1 | fruit | 1108 | 1135 | 0.90 | Ingestive | Oral-Manual | Anterior |
| ‘nice juvie feeding’ | Pacoca | AF | Sequence 1 | fruit | 1135 | 1187 | 1.73 | Masticate |  |  |
| ‘nice juvie feeding’ | Pacoca | AF | Sequence 1 | fruit | 1187 | 1211 | 0.80 | Ingestive | Oral-Manual | Anterior |
| ‘nice juvie feeding’ | Pacoca | AF | Sequence 1 | fruit | 1211 | 1239 | 0.93 | Masticate |  |  |
| ‘nice juvie feeding’ | Pacoca | AF | Sequence 1 | fruit | 1239 | 1256 | 0.57 | Ingestive | Oral-Manual | Anterior |
| ‘nice juvie feeding’ | Pacoca | AF | Sequence 1 | fruit | 1256 | 1324 | 2.27 | Masticate |  |  |
| ‘nice juvie feeding’ | Pacoca | AF | Sequence 1 | fruit | 1324 | 1346 | 0.73 | Ingestive | Oral-Manual | Anterior |
| ‘nice juvie marmalatta_1’ | Pacoca | AF | Sequence 1 | fruit | 1 | 24 | 0.77 | Masticate |  |  |
| ‘nice juvie marmalatta_1’ | Pacoca | AF | Sequence 1 | fruit | 24 | 25 | 0.03 | Ingestive | Oral-Manual | Anterior |
| ‘nice juvie marmalatta_1’ | Pacoca | AF | Sequence 1 | fruit | 25 | 99 | 2.47 | Masticate |  |  |
| ‘nice juvie marmalatta_1’ | Pacoca | AF | Sequence 1 | fruit | 119 | 179 | 2.00 | Ingestive | Oral-Manual | Anterior |
| ‘nice juvie marmalatta_1’ | Pacoca | AF | Sequence 1 | fruit | 218 | 245 | 0.90 | Ingestive | Oral-Manual | Anterior |
| ‘nice juvie marmalatta_1’ | Pacoca | AF | Sequence 1 | fruit | 245 | 328 | 2.77 | Masticate |  |  |
| ‘nice juvie marmalatta_1’ | Pacoca | AF | Sequence 1 | fruit | 377 | 410 | 1.10 | Ingestive | Oral-Manual | Anterior |
| ‘nice juvie marmalatta_1’ | Pacoca | AF | Sequence 1 | fruit | 462 | 484 | 0.73 | Ingestive | Oral-Manual | Anterior |
| ‘nice juvie marmalatta_2’ | Pacoca | AF | Sequence 1 | fruit | 22 | 26 | 0.13 | Ingestive | Oral-Manual | Anterior |
| ‘nice juvie marmalatta_2’ | Pacoca | AF | Sequence 1 | fruit | 51 | 73 | 0.73 | Ingestive | Oral-Manual | Anterior |
| ‘nice juvie marmalatta_2’ | Pacoca | AF | Sequence 1 | fruit | 129 | 149 | 0.67 | Ingestive | Oral-Manual | Anterior |
| ‘nice juvie marmalatta_2’ | Pacoca | AF | Sequence 1 | fruit | 185 | 230 | 1.50 | Ingestive | Oral-Manual | Anterior |
| ‘nice monk green fruit_1’ | Patricia | SAF | Sequence 1 | fruit | 24 | 47 | 0.77 | Ingestive | Oral-Manual | Anterior |
| ‘nice monk green fruit_1’ | Patricia | SAF | Sequence 1 | fruit | 72 | 108 | 1.20 | Ingestive | Oral-Manual | Anterior |
| ‘nice monk green fruit_1’ | Patricia | SAF | Sequence 1 | fruit | 173 | 189 | 0.53 | Ingestive | Oral-Manual | Anterior |
| ‘nice monk green fruit_2’ | Patricia | SAF | Sequence 1 | fruit | 24 | 64 | 1.33 | Ingestive | Oral-Manual | Anterior |
| ‘nice monk green fruit_2’ | Patricia | SAF | Sequence 1 | fruit | 88 | 127 | 1.30 | Ingestive | Oral-Manual | Anterior |
| ‘nice monk green fruit_2’ | Patricia | SAF | Sequence 1 | fruit | 166 | 188 | 0.73 | Ingestive | Oral-Manual | Anterior |
| ‘nice monk green fruit_2’ | Patricia | SAF | Sequence 1 | fruit | 241 | 242 | 0.03 |  |  |  |
| ‘nice monk green fruit_2’ | Patricia | SAF | Sequence 1 | fruit | 281 | 333 | 1.73 | Ingestive | Oral-Manual | Anterior |
| ‘nice monk green fruit_2’ | Patricia | SAF | Sequence 1 | fruit | 333 | 607 | 9.13 | Masticate |  |  |
| ‘nice monk green fruit_3’ | Patricia | SAF | Sequence 1 | fruit | 17 | 41 | 0.80 | Ingestive | Oral-Manual | Anterior |
| ‘nice monk green fruit_3’ | Patricia | SAF | Sequence 1 | fruit | 103 | 128 | 0.83 | Ingestive | Oral-Manual | Anterior |
| ‘nice monk green fruit_3’ | Patricia | SAF | Sequence 1 | fruit | 157 | 176 | 0.63 | Ingestive | Oral-Manual | Posterior |
| ‘nice monk green fruit_3’ | Patricia | SAF | Sequence 1 | fruit | 205 | 224 | 0.63 | Ingestive | Oral-Manual | Anterior |
| ‘nice monk green fruit_3’ | Patricia | SAF | Sequence 1 | fruit | 224 | 311 | 2.90 | Masticate |  |  |
| ‘nice monk green fruit_3’ | Patricia | SAF | Sequence 1 | fruit | 311 | 358 | 1.57 | Ingestive | Oral-Manual | Anterior |
| ‘nice monk green fruit_3’ | Patricia | SAF | Sequence 1 | fruit | 400 | 424 | 0.80 | Ingestive | Oral-Manual | Anterior |
| ‘nice monk green fruit_3’ | Patricia | SAF | Sequence 1 | fruit | 488 | 542 | 1.80 | Ingestive | Oral-Manual | Anterior |
| ‘nice monk green fruit_3’ | Patricia | SAF | Sequence 1 | fruit | 644 | 704 | 2.00 | Ingestive | Oral-Manual | Posterior |
| ‘nice monk green fruit_3’ | Patricia | SAF | Sequence 1 | fruit | 737 | 757 | 0.67 | Ingestive | Oral-Manual | Anterior |
| ‘nice monk green fruit_3’ | Patricia | SAF | Sequence 1 | fruit | 805 | 815 | 0.33 | Ingestive | Oral-Manual | Anterior |
| ‘nice monk green fruit_3’ | Patricia | SAF | Sequence 1 | fruit | 848 | 889 | 1.37 | Ingestive | Oral-Manual | Anterior |
| ‘nice monk green fruit_3’ | Patricia | SAF | Sequence 1 | fruit | 1030 | 1059 | 0.97 | Ingestive | Oral-Manual | Anterior |
| ‘nice monk green fruit_3’ | Patricia | SAF | Sequence 1 | fruit | 1105 | 1155 | 1.67 | Ingestive | Oral-Manual | Anterior |
| ‘nice monk green fruit_3’ | Patricia | SAF | Sequence 1 | fruit | 1233 | 1308 | 2.50 | Ingestive | Oral-Manual | Posterior |
| ‘nice monk green fruit_3’ | Patricia | SAF | Sequence 1 | fruit | 1343 | 1357 | 0.47 | Ingestive | Oral-Manual | Anterior |
| ‘nice monk green fruit_3’ | Patricia | SAF | Sequence 1 | fruit | 1454 | 1479 | 0.83 | Ingestive | Oral-Manual | Anterior |
| ‘nice monk green fruit_3’ | Patricia | SAF | Sequence 1 | fruit | 1537 | 1549 | 0.40 | Ingestive | Oral-Manual | Anterior |
| ‘nice monk green fruit_3’ | Patricia | SAF | Sequence 1 | fruit | 1597 | 1604 | 0.23 | Ingestive | Oral-Manual | Anterior |
| ‘nice monk green fruit_3’ | Patricia | SAF | Sequence 1 | fruit | 1643 | 1655 | 0.40 | Ingestive | Oral-Manual | Anterior |
| ‘nice monk green fruit_3’ | Patricia | SAF | Sequence 1 | fruit | 1753 | 1760 | 0.23 | Ingestive | Oral-Manual | Anterior |
| ‘nice monk green fruit_3’ | Patricia | SAF | Sequence 1 | fruit | 1760 | 1872 | 3.73 | Masticate |  |  |
| ‘nice pc biting_1’ | Presente | SAM | Sequence 1 | insects | 1 | 58 | 1.90 | Ingestive | Oral-Manual | Anterior |
| ‘nice pc biting_1’ | Presente | SAM | Sequence 1 | insects | 169 | 356 | 6.23 | Ingestive | Oral-Manual | Posterior |
| ‘nice pc biting_1’ | Presente | SAM | Sequence 1 | insects | 408 | 436 | 0.93 | Ingestive | Oral-Manual | Anterior |
| ‘nice pc biting_1’ | Presente | SAM | Sequence 1 | insects | 436 | 622 | 6.20 | Ingestive | Oral-Manual | Posterior |
| ‘nice pc biting_1’ | Presente | SAM | Sequence 1 | insects | 622 | 646 | 0.80 | Ingestive | Oral-Manual | Anterior |
| ‘nice pc biting_1’ | Presente | SAM | Sequence 1 | insects | 667 | 684 | 0.57 | Ingestive | Oral-Manual | Anterior |
| ‘nice pc biting_1’ | Presente | SAM | Sequence 1 | insects | 724 | 785 | 2.03 | Ingestive | Oral-Manual | Anterior |
| ‘nice pc biting_1’ | Presente | SAM | Sequence 1 | insects | 785 | 834 | 1.63 | Masticate |  |  |
| ‘nice pc biting_1’ | Presente | SAM | Sequence 1 | insects | 834 | 844 | 0.33 | Ingestive | Oral-Manual | Anterior |
| ‘nice pc biting_1’ | Presente | SAM | Sequence 1 | insects | 858 | 918 | 2.00 | Ingestive | Oral-Manual | Posterior |
| ‘nice pc biting_2’ | Presente | SAM | Sequence 1 | insects | 85 | 111 | 0.87 | Ingestive | Oral-Manual | Anterior |
| ‘nice pc biting_2’ | Presente | SAM | Sequence 1 | insects | 111 | 205 | 3.13 | Ingestive | Oral-Manual | Posterior |
| ‘nice pc biting_3’ | Presente | SAM | Sequence 1 | insects | 1 | 21 | 0.67 | Ingestive | Oral-Manual | Anterior |
| ‘nice pc biting_3’ | Presente | SAM | Sequence 1 | insects | 21 | 128 | 3.57 | Masticate |  |  |
| ‘nice pc biting_3’ | Presente | SAM | Sequence 1 | insects | 128 | 145 | 0.57 | Ingestive | Oral-Manual | Anterior |
| ‘nice pc biting_3’ | Presente | SAM | Sequence 1 | insects | 145 | 226 | 2.70 | Masticate |  |  |
| ‘nice pc biting_3’ | Presente | SAM | Sequence 1 | insects | 226 | 243 | 0.57 | Ingestive | Oral-Manual | Anterior |
| ‘nice pc biting_3’ | Presente | SAM | Sequence 1 | insects | 243 | 261 | 0.60 | Masticate |  |  |
| ‘nice pc biting_3’ | Presente | SAM | Sequence 1 | insects | 261 | 269 | 0.27 | Ingestive | Oral-Manual | Anterior |
| ‘nice pc biting_3’ | Presente | SAM | Sequence 1 | insects | 294 | 361 | 2.23 | Ingestive | Oral-Manual | Anterior |
| ‘nice pc biting_3’ | Presente | SAM | Sequence 1 | insects | 361 | 397 | 1.20 | Masticate |  |  |
| ‘nice pc biting_3’ | Presente | SAM | Sequence 1 | insects | 397 | 405 | 0.27 | Ingestive | Oral-Manual | Anterior |
| ‘nice pc biting_3’ | Presente | SAM | Sequence 1 | insects | 420 | 426 | 0.20 | Ingestive | Oral-Manual | Anterior |
| ‘nice pc biting_3’ | Presente | SAM | Sequence 1 | insects | 426 | 455 | 0.97 | Masticate |  |  |
| ‘nice pc biting_3’ | Presente | SAM | Sequence 1 | insects | 455 | 499 | 1.47 | Ingestive | Oral-Manual | Anterior |
| ‘nice pc biting_3’ | Presente | SAM | Sequence 1 | insects | 499 | 525 | 0.87 | Masticate |  |  |
| ‘nice pc biting_3’ | Presente | SAM | Sequence 1 | insects | 525 | 541 | 0.53 | Ingestive | Oral-Manual | Posterior |
| ‘nice pc biting_3’ | Presente | SAM | Sequence 1 | insects | 541 | 589 | 1.60 | Masticate |  |  |
| ‘nice pc biting_4’ | Presente | SAM | Sequence 1 | insects | 1 | 63 | 2.07 | Ingestive | Oral-Manual | Posterior |
| ‘nice pc biting_4’ | Presente | SAM | Sequence 1 | insects | 113 | 232 | 3.97 | Ingestive | Oral-Manual | Posterior |
| ‘nice pc biting_4’ | Presente | SAM | Sequence 1 | insects | 273 | 307 | 1.13 | Ingestive | Oral-Manual | Anterior |
| ‘nice pc biting_4’ | Presente | SAM | Sequence 1 | insects | 307 | 361 | 1.80 | Ingestive | Oral-Manual | Anterior |
| ‘nice pc biting_4’ | Presente | SAM | Sequence 1 | insects | 361 | 448 | 2.90 | Ingestive | Oral-Manual | Posterior |
| ‘nice pc biting_4’ | Presente | SAM | Sequence 1 | insects | 487 | 523 | 1.20 | Ingestive | Oral-Manual | Anterior |
| ‘nice pc biting_4’ | Presente | SAM | Sequence 1 | insects | 551 | 561 | 0.33 | Ingestive | Oral-Manual | Anterior |
| ‘nice pc biting_4’ | Presente | SAM | Sequence 1 | insects | 582 | 595 | 0.43 | Ingestive | Oral-Manual | Anterior |
| ‘nice pc biting_4’ | Presente | SAM | Sequence 1 | insects | 595 | 654 | 1.97 | Ingestive | Oral-Manual | Posterior |
| ‘nice pc biting_4’ | Presente | SAM | Sequence 1 | insects | 654 | 701 | 1.57 | Masticate |  |  |
| ‘nice pc biting_4’ | Presente | SAM | Sequence 1 | insects | 701 | 708 | 0.23 | Ingestive | Oral-Manual | Anterior |
| ‘nice pc biting_4’ | Presente | SAM | Sequence 1 | insects | 749 | 750 | 0.03 | Ingestive | Oral-Manual | Anterior |
| ‘nice pc biting_4’ | Presente | SAM | Sequence 1 | insects | 816 | 848 | 1.07 | Ingestive | Oral-Manual | Anterior |
| ‘nice pc biting_4’ | Presente | SAM | Sequence 1 | insects | 848 | 926 | 2.60 | Masticate |  |  |
| ‘nice pc biting_4’ | Presente | SAM | Sequence 1 | insects | 926 | 1015 | 2.97 | Ingestive | Oral-Manual | Anterior |
| ‘nice pc biting_4’ | Presente | SAM | Sequence 1 | insects | 1015 | 1089 | 2.47 | Masticate |  |  |
| ‘nice tuberosity_1’ | Coco | AM | Sequence 1 | USO | 205 | 263 | 1.93 | Ingestive | Oral-Manual | Anterior |
| ‘nice tuberosity_1’ | Coco | AM | Sequence 1 | USO | 263 | 363 | 3.33 | Masticate |  |  |
| ‘nice tuberosity_1’ | Coco | AM | Sequence 1 | USO | 363 | 389 | 0.87 | Ingestive | Oral-Manual | Posterior |
| ‘nice tuberosity_1’ | Coco | AM | Sequence 1 | USO | 389 | 527 | 4.60 | Masticate |  |  |
| ‘nice tuberosity_1’ | Coco | AM | Sequence 1 | USO | 527 | 590 | 2.10 | Ingestive | Oral-Manual | Anterior |
| ‘nice tuberosity_1’ | Coco | AM | Sequence 1 | USO | 590 | 630 | 1.33 | Masticate |  |  |
| ‘nice tuberosity_1’ | Coco | AM | Sequence 1 | USO | 630 | 713 | 2.77 | Ingestive | Oral-Manual | Anterior |
| ‘nice tuberosity_1’ | Coco | AM | Sequence 1 | USO | 713 | 718 | 0.17 | Masticate |  |  |
| ‘nice tuberosity_1’ | Coco | AM | Sequence 1 | USO | 718 | 840 | 4.07 | Ingestive | Oral-Manual | Anterior |
| ‘nice tuberosity_1’ | Coco | AM | Sequence 1 | USO | 840 | 1029 | 6.30 | Masticate |  |  |
| ‘nice tuberosity_2’ | Coco | AM | Sequence 1 | USO | 1 | 34 | 1.10 | Ingestive | Oral-Manual | Anterior |
| ‘nice tuberosity_2’ | Coco | AM | Sequence 1 | USO | 34 | 82 | 1.60 | Masticate |  |  |
| ‘nice tuberosity_2’ | Coco | AM | Sequence 1 | USO | 82 | 133 | 1.70 | Ingestive | Oral-Manual | Anterior |
| ‘nice tuberosity_2’ | Coco | AM | Sequence 1 | USO | 133 | 170 | 1.23 | Masticate |  |  |
| ‘nice tuberosity_2’ | Coco | AM | Sequence 1 | USO | 170 | 220 | 1.67 | Ingestive | Oral-Manual | Anterior |
| ‘nice tuberosity_2’ | Coco | AM | Sequence 1 | USO | 464 | 471 | 0.23 | Ingestive | Oral-Manual | Anterior |
| ‘nice tuberosity_2’ | Coco | AM | Sequence 1 | USO | 550 | 601 | 1.70 | Ingestive | Oral-Manual | Anterior |
| ‘nice tuberosity_2’ | Coco | AM | Sequence 1 | USO | 637 | 679 | 1.40 | Ingestive | Oral-Manual | Anterior |
| ‘nice tuberosity_2’ | Coco | AM | Sequence 1 | USO | 745 | 785 | 1.33 | Ingestive | Oral-Manual | Anterior |
| ‘nice tuberosity_2’ | Coco | AM | Sequence 1 | USO | 840 | 863 | 0.77 | Ingestive | Oral-Manual | Anterior |
| ‘nice tuberosity_2’ | Coco | AM | Sequence 1 | USO | 948 | 981 | 1.10 | Ingestive | Oral-Manual | Anterior |
| ‘nice tuberosity_2’ | Coco | AM | Sequence 1 | USO | 1007 | 1114 | 3.57 | Ingestive | Oral-Manual | Anterior |
| ‘nice tuberosity_2’ | Coco | AM | Sequence 1 | USO | 1114 | 1150 | 1.20 | Ingestive | Oral-Manual | Posterior |
| ‘nice tuberosity_2’ | Coco | AM | Sequence 1 | USO | 1182 | 1213 | 1.03 | Ingestive | Oral-Manual | Posterior |
| ‘nice tuberosity_3’ | Coco | AM | Sequence 1 | USO | 1 | 64 | 2.10 | Ingestive | Oral-Manual | Anterior |
| ‘nice tuberosity_3’ | Coco | AM | Sequence 1 | USO | 164 | 213 | 1.63 | Ingestive | Oral-Manual | Anterior |
| ‘nice tuberosity_3’ | Coco | AM | Sequence 1 | USO | 360 | 393 | 1.10 | Ingestive | Oral-Manual | Anterior |
| ‘nice tuberosity_3’ | Coco | AM | Sequence 1 | USO | 567 | 614 | 1.57 | Ingestive | Oral-Manual | Anterior |
| ‘nice tuberosity_3’ | Coco | AM | Sequence 1 | USO | 673 | 679 | 0.20 | Ingestive | Oral-Manual | Anterior |
| ‘nice tuberosity_3’ | Coco | AM | Sequence 1 | USO | 679 | 712 | 1.10 | Ingestive | Oral-Manual | Posterior |
| ‘nice tuberosity_4’ | Coco | AM | Sequence 1 | USO | 67 | 115 | 1.60 | Ingestive | Oral-Manual | Posterior |
| ‘nice tuberosity_4’ | Coco | AM | Sequence 1 | USO | 135 | 217 | 2.73 | Ingestive | Oral-Manual | Anterior |
| ‘nice tuberosity_4’ | Coco | AM | Sequence 1 | USO | 217 | 258 | 1.37 | Masticate |  |  |
| ‘nice tuberosity_4’ | Coco | AM | Sequence 1 | USO | 258 | 285 | 0.90 | Ingestive | Oral-Manual | Anterior |
| ‘nice tuberosity_4’ | Coco | AM | Sequence 1 | USO | 308 | 336 | 0.93 | Ingestive | Oral-Manual | Posterior |
| ‘nice tuberosity_4’ | Coco | AM | Sequence 1 | USO | 431 | 478 | 1.57 | Ingestive | Oral-Manual | Anterior |
| ‘nice tuberosity_4’ | Coco | AM | Sequence 1 | USO | 639 | 687 | 1.60 | Ingestive | Oral-Manual | Anterior |
| ‘nice tuberosity_5’ | Coco | AM | Sequence 1 | USO | 16 | 69 | 1.77 | Ingestive | Oral-Manual | Anterior |
| ‘nice tuberosity_5’ | Coco | AM | Sequence 1 | USO | 153 | 217 | 2.13 | Ingestive | Oral-Manual | Anterior |
| ‘nice tuberosity_5’ | Coco | AM | Sequence 1 | USO | 275 | 323 | 1.60 | Ingestive | Oral-Manual | Anterior |
| ‘nice tuberosity_5’ | Coco | AM | Sequence 1 | USO | 444 | 492 | 1.60 | Ingestive | Oral-Manual | Anterior |
| ‘nice tuberosity_5’ | Coco | AM | Sequence 1 | USO | 492 | 582 | 3.00 | Ingestive | Oral-Manual | Posterior |
| ‘nice tuberosity_5’ | Coco | AM | Sequence 1 | USO | 656 | 662 | 0.20 | Ingestive | Oral-Manual | Anterior |
| ‘palm 1_1’ | Tais | SAF | Sequence 1 | Cane | 22 | 64 | 1.40 | Ingestive | Oral | Anterior |
| ‘palm 1_1’ | Tais | SAF | Sequence 1 | Cane | 183 | 220 | 1.23 | Ingestive | Oral | Anterior |
| ‘palm 1_1’ | Tais | SAF | Sequence 1 | Cane | 298 | 308 | 0.33 | Ingestive | Oral | Anterior |
| ‘palm 1_1’ | Tais | SAF | Sequence 1 | Cane | 325 | 401 | 2.53 | Ingestive | Oral | Anterior |
| ‘palm 1_1’ | Tais | SAF | Sequence 1 | Cane | 443 | 470 | 0.90 | Ingestive | Oral | Anterior |
| ‘palm 1_2’ | Tais | SAF | Sequence 1 | Cane | 15 | 83 | 2.27 | Ingestive | Oral | Anterior |
| ‘palm 1_3’ | Tais | SAF | Sequence 1 | Cane | 1 | 58 | 1.90 | Ingestive | Oral | Anterior |
| ‘palm 1_3’ | Tais | SAF | Sequence 1 | Cane | 231 | 249 | 0.60 | Ingestive | Oral | Anterior |
| ‘palm 1_3’ | Tais | SAF | Sequence 1 | Cane | 363 | 422 | 1.97 | Ingestive | Oral | Anterior |
| ‘palm 1_3’ | Tais | SAF | Sequence 1 | Cane | 442 | 475 | 1.10 | Ingestive | Oral | Anterior |
| ‘palm 1_3’ | Tais | SAF | Sequence 1 | Cane | 506 | 547 | 1.37 | Ingestive | Oral | Anterior |
| ‘palm 1_3’ | Tais | SAF | Sequence 1 | Cane | 808 | 842 | 1.13 | Ingestive | Oral | Anterior |
| ‘palm 1_3’ | Tais | SAF | Sequence 1 | Cane | 899 | 946 | 1.57 | Ingestive | Oral | Anterior |
| ‘palm biting for bugs_1’ | Dita | AF | Sequence 1 | insects | 253 | 267 | 0.47 | Ingestive | Oral-Manual | Anterior |
| ‘palm biting for bugs_1’ | Dita | AF | Sequence 1 | insects | 267 | 309 | 1.40 | Masticate |  |  |
| ‘palm biting for bugs_1’ | Dita | AF | Sequence 1 | insects | 309 | 318 | 0.30 | Ingestive | Oral-Manual | Anterior |
| ‘palm biting for bugs_1’ | Dita | AF | Sequence 1 | insects | 318 | 367 | 1.63 | Masticate |  |  |
| ‘palm biting for bugs_1’ | Dita | AF | Sequence 1 | insects | 402 | 411 | 0.30 | Ingestive | Oral-Manual | Anterior |
| ‘palm biting for bugs_1’ | Dita | AF | Sequence 1 | insects | 411 | 436 | 0.83 | Masticate |  |  |
| ‘palm biting for bugs_1’ | Dita | AF | Sequence 1 | insects | 436 | 437 | 0.03 | Ingestive | Oral-Manual | Anterior |
| ‘palm biting for bugs_1’ | Dita | AF | Sequence 1 | insects | 437 | 494 | 1.90 | Masticate |  |  |
| ‘palm biting for bugs_1’ | Dita | AF | Sequence 1 | insects | 494 | 530 | 1.20 | Ingestive | Oral-Manual | Anterior |
| ‘palm biting for bugs_1’ | Dita | AF | Sequence 1 | insects | 549 | 553 | 0.13 | Ingestive | Oral-Manual | Anterior |
| ‘palm biting for bugs_1’ | Dita | AF | Sequence 1 | insects | 553 | 685 | 4.40 | Masticate |  |  |
| ‘palm biting for bugs_2’ | Dita | AF | Sequence 1 | insects | 1 | 9 | 0.27 | Ingestive | Oral | Anterior |
| ‘palm biting for bugs_2’ | Dita | AF | Sequence 1 | insects | 76 | 112 | 1.20 | Ingestive | Oral | Anterior |
| ‘palm biting for bugs_2’ | Dita | AF | Sequence 1 | insects | 141 | 160 | 0.63 | Ingestive | Oral | Anterior |
| ‘palm biting for bugs_2’ | Dita | AF | Sequence 1 | insects | 203 | 222 | 0.63 | Ingestive | Oral | Anterior |
| ‘palm biting for bugs_2’ | Dita | AF | Sequence 1 | insects | 309 | 333 | 0.80 | Ingestive | Oral | Anterior |
| ‘palm biting for bugs_2’ | Dita | AF | Sequence 1 | insects | 354 | 398 | 1.47 | Ingestive | Oral | Anterior |
| ‘palm biting for bugs_2’ | Dita | AF | Sequence 1 | insects | 424 | 448 | 0.80 | Ingestive | Oral | Anterior |
| ‘palm biting for bugs_2’ | Dita | AF | Sequence 1 | insects | 468 | 490 | 0.73 | Ingestive | Oral | Anterior |
| ‘palm biting for bugs_2’ | Dita | AF | Sequence 1 | insects | 510 | 543 | 1.10 | Ingestive | Oral | Anterior |
| ‘palm biting for bugs_2’ | Dita | AF | Sequence 1 | insects | 566 | 611 | 1.50 | Ingestive | Oral | Anterior |
| ‘palm biting for bugs_2’ | Dita | AF | Sequence 1 | insects | 730 | 768 | 1.27 | Ingestive | Oral | Anterior |
| ‘palm biting for bugs_2’ | Dita | AF | Sequence 1 | insects | 835 | 880 | 1.50 | Ingestive | Oral | Anterior |
| ‘palm biting for bugs_2’ | Dita | AF | Sequence 1 | insects | 948 | 968 | 0.67 | Ingestive | Oral | Anterior |
| ‘piasava nutting’ | Piassava | AF | Sequence 1 | Piaçava | 14 | 41 | 0.90 | Manual |  |  |
| ‘piasava nutting’ | Piassava | AF | Sequence 1 | Piaçava | 41 | 243 | 6.73 | Manual |  |  |
| ‘piasava nutting’ | Piassava | AF | Sequence 1 | Piaçava | 498 | 541 | 1.43 | Manual |  |  |
| ‘piasava nutting’ | Piassava | AF | Sequence 1 | Piaçava | 773 | 808 | 1.17 | Manual |  |  |
| ‘piasava nutting’ | Piassava | AF | Sequence 1 | Piaçava | 873 | 927 | 1.80 | Manual |  |  |
| ‘random monkeys’ | Chuchu | AF | Sequence 1 | Piaçava | 25 | 150 | 4.17 | Manual |  |  |
| ‘random monkeys’ | Chuchu | AF | Sequence 1 | Piaçava | 362 | 440 | 2.60 | Manual |  |  |
| ‘random monkeys’ | Chuchu | AF | Sequence 1 | Piaçava | 556 | 596 | 1.33 | Manual |  |  |
| ‘random monkeys’ | Chuchu | AF | Sequence 1 | Piaçava | 826 | 847 | 0.70 | Ingestive | Oral-Manual | Anterior |
| ‘random monkeys’ | Chuchu | AF | Sequence 1 | Piaçava | 879 | 921 | 1.40 | Manual |  |  |
| ‘random monkeys’ | Chuchu | AF | Sequence 1 | Piaçava | 1141 | 1152 | 0.37 | Ingestive | Oral-Manual | Anterior |
| ‘random monkeys’ | Chuchu | AF | Sequence 1 | Piaçava | 1204 | 1264 | 2.00 | Ingestive | Oral-Manual | Anterior |
| ‘random monkeys’ | Chuchu | AF | Sequence 1 | Piaçava | 1413 | 1423 | 0.33 | Ingestive | Oral-Manual | Anterior |
| ‘random monkeys’ | Chuchu | AF | Sequence 1 | Piaçava | 1423 | 1459 | 1.20 | Ingestive | Oral-Manual | Posterior |
| ‘random monkeys’ | Chuchu | AF | Sequence 1 | Piaçava | 1459 | 1611 | 5.07 | Masticate |  |  |
| ‘random monkeys’ | Chuchu | AF | Sequence 1 | Piaçava | 1611 | 1614 | 0.10 | Ingestive | Oral-Manual | Anterior |
| ‘SC good 3_1’ | Catu | AM | Sequence 1 | Cane | 1 | 3 | 0.07 | Ingestive | Oral-Manual | Posterior |
| ‘SC good 3_1’ | Catu | AM | Sequence 1 | Cane | 3 | 42 | 1.30 | Masticate |  |  |
| ‘SC good 3_1’ | Catu | AM | Sequence 1 | Cane | 42 | 53 | 0.37 | Ingestive | Oral-Manual | Anterior |
| ‘SC good 3_1’ | Catu | AM | Sequence 1 | Cane | 53 | 130 | 2.57 | Masticate |  |  |
| ‘SC good 3_1’ | Catu | AM | Sequence 1 | Cane | 218 | 238 | 0.67 | Ingestive | Oral-Manual | Anterior |
| ‘SC good 3_1’ | Catu | AM | Sequence 1 | Cane | 238 | 295 | 1.90 | Ingestive | Oral-Manual | Posterior |
| ‘SC good 3_1’ | Catu | AM | Sequence 1 | Cane | 325 | 365 | 1.33 | Ingestive | Oral-Manual | Anterior |
| ‘SC good 3_1’ | Catu | AM | Sequence 1 | Cane | 365 | 391 | 0.87 | Ingestive | Oral-Manual | Posterior |
| ‘SC good 3_1’ | Catu | AM | Sequence 1 | Cane | 479 | 499 | 0.67 | Ingestive | Oral-Manual | Anterior |
| ‘SC good 3_1’ | Catu | AM | Sequence 1 | Cane | 513 | 592 | 2.63 | Ingestive | Oral-Manual | Anterior |
| ‘SC good 3_1’ | Catu | AM | Sequence 1 | Cane | 627 | 707 | 2.67 | Ingestive | Oral-Manual | Anterior |
| ‘SC good 3_1’ | Catu | AM | Sequence 1 | Cane | 775 | 788 | 0.43 | Ingestive | Oral-Manual | Anterior |
| ‘SC good 3_1’ | Catu | AM | Sequence 1 | Cane | 788 | 856 | 2.27 | Masticate |  |  |
| ‘SC good 3_1’ | Catu | AM | Sequence 1 | Cane | 856 | 921 | 2.17 | Ingestive | Oral-Manual | Anterior |
| ‘SC good 3_1’ | Catu | AM | Sequence 1 | Cane | 946 | 983 | 1.23 | Ingestive | Oral-Manual | Anterior |
| ‘SC good 3_2’ | Catu | AM | Sequence 1 | Cane | 45 | 54 | 0.30 | Ingestive | Oral-Manual | Posterior |
| ‘SC good 3_2’ | Catu | AM | Sequence 1 | Cane | 54 | 85 | 1.03 | Ingestive | Oral-Manual | Anterior |
| ‘SC good 3_2’ | Catu | AM | Sequence 1 | Cane | 85 | 158 | 2.43 | Ingestive | Oral-Manual | Posterior |
| ‘SC good 3_2’ | Catu | AM | Sequence 1 | Cane | 223 | 229 | 0.20 | Ingestive | Oral-Manual | Anterior |
| ‘SC good 3_2’ | Catu | AM | Sequence 1 | Cane | 249 | 333 | 2.80 | Ingestive | Oral-Manual | Posterior |
| ‘SC good 3_2’ | Catu | AM | Sequence 1 | Cane | 333 | 433 | 3.33 | Masticate |  |  |
| ‘SC good 3_2’ | Catu | AM | Sequence 1 | Cane | 465 | 542 | 2.57 | Ingestive | Oral-Manual | Posterior |
| ‘SC good 3_2’ | Catu | AM | Sequence 1 | Cane | 608 | 664 | 1.87 | Ingestive | Oral-Manual | Posterior |
| ‘SC good 3_2’ | Catu | AM | Sequence 1 | Cane | 757 | 825 | 2.27 | Ingestive | Oral-Manual | Posterior |
| ‘SC good 3_2’ | Catu | AM | Sequence 1 | Cane | 867 | 959 | 3.07 | Ingestive | Oral-Manual | Posterior |
| ‘SC good 3_2’ | Catu | AM | Sequence 1 | Cane | 1004 | 1069 | 2.17 | Ingestive | Oral-Manual | Posterior |
| ‘SC good 3_3’ | Catu | AM | Sequence 1 | Cane | 1 | 22 | 0.70 | Ingestive | Oral-Manual | Anterior |
| ‘SC good 3_3’ | Catu | AM | Sequence 1 | Cane | 22 | 95 | 2.43 | Masticate |  |  |
| ‘SC good 3_3’ | Catu | AM | Sequence 1 | Cane | 95 | 224 | 4.30 | Ingestive | Oral-Manual | Posterior |
| ‘SC good 3_3’ | Catu | AM | Sequence 1 | Cane | 276 | 292 | 0.53 | Ingestive | Oral-Manual | Anterior |
| ‘SC good 3_3’ | Catu | AM | Sequence 1 | Cane | 292 | 367 | 2.50 | Ingestive | Oral-Manual | Posterior |
| ‘SC good 3_3’ | Catu | AM | Sequence 1 | Cane | 422 | 424 | 0.07 | Ingestive | Oral-Manual | Anterior |
| ‘SC good 4_1’ | Teninha | AF | Sequence 1 | Cane | 23 | 52 | 0.97 | Ingestive | Oral | Anterior |
| ‘SC good 4_1’ | Teninha | AF | Sequence 1 | Cane | 189 | 235 | 1.53 | Ingestive | Oral | Anterior |
| ‘SC good 4_1’ | Teninha | AF | Sequence 1 | Cane | 275 | 310 | 1.17 | Ingestive | Oral-Manual | Anterior |
| ‘SC good 4_1’ | Teninha | AF | Sequence 1 | Cane | 375 | 380 | 0.17 | Ingestive | Oral | Anterior |
| ‘SC good 4_1’ | Teninha | AF | Sequence 1 | Cane | 438 | 455 | 0.57 | Ingestive | Oral | Anterior |
| ‘SC good 4_2’ | Teninha | AF | Sequence 1 | Cane | 1 | 114 | 3.77 | Ingestive | Oral-Manual | Posterior |
| ‘SC good 4_2’ | Teninha | AF | Sequence 1 | Cane | 185 | 299 | 3.80 | Ingestive | Oral-Manual | Posterior |
| ‘SC good 4_2’ | Teninha | AF | Sequence 1 | Cane | 329 | 382 | 1.77 | Ingestive | Oral-Manual | Posterior |
| ‘SC good 4_2’ | Teninha | AF | Sequence 1 | Cane | 414 | 451 | 1.23 | Ingestive | Oral-Manual | Posterior |
| ‘SC good 4_2’ | Teninha | AF | Sequence 1 | Cane | 480 | 541 | 2.03 | Ingestive | Oral-Manual | Posterior |
| ‘SC good 4_2’ | Teninha | AF | Sequence 1 | Cane | 569 | 620 | 1.70 | Ingestive | Oral-Manual | Posterior |
| ‘SC good 4_2’ | Teninha | AF | Sequence 1 | Cane | 748 | 801 | 1.77 | Ingestive | Oral-Manual | Anterior |
| ‘SC good 4_2’ | Teninha | AF | Sequence 1 | Cane | 822 | 918 | 3.20 | Ingestive | Oral-Manual | Anterior |
| ‘SC good 4_2’ | Teninha | AF | Sequence 1 | Cane | 945 | 948 | 0.10 | Ingestive | Oral-Manual | Posterior |
| ‘SC good 4_2’ | Teninha | AF | Sequence 1 | Cane | 970 | 1009 | 1.30 | Ingestive | Oral-Manual | Posterior |
| ‘SC good 4_2’ | Teninha | AF | Sequence 1 | Cane | 1034 | 1055 | 0.70 | Ingestive | Oral-Manual | Anterior |
| ‘SC good 4_2’ | Teninha | AF | Sequence 1 | Cane | 1072 | 1108 | 1.20 | Ingestive | Oral-Manual | Posterior |
| ‘SC good 4_2’ | Teninha | AF | Sequence 1 | Cane | 1120 | 1143 | 0.77 | Ingestive | Oral-Manual | Posterior |
| ‘SC good 4_2’ | Teninha | AF | Sequence 1 | Cane | 1204 | 1226 | 0.73 | Ingestive | Oral-Manual | Anterior |
| ‘SC good 4_2’ | Teninha | AF | Sequence 1 | Cane | 1255 | 1271 | 0.53 | Ingestive | Oral-Manual | Posterior |
| ‘SC good 4_2’ | Teninha | AF | Sequence 1 | Cane | 1347 | 1397 | 1.67 | Ingestive | Oral-Manual | Posterior |
| ‘SC good 4_2’ | Teninha | AF | Sequence 1 | Cane | 1431 | 1477 | 1.53 | Ingestive | Oral-Manual | Posterior |
| ‘SC good 4_2’ | Teninha | AF | Sequence 1 | Cane | 1497 | 1500 | 0.10 | Ingestive | Oral-Manual | Posterior |
| ‘SC good 4_2’ | Teninha | AF | Sequence 1 | Cane | 1532 | 1597 | 2.17 | Ingestive | Oral-Manual | Anterior |
| ‘SC good 4_2’ | Teninha | AF | Sequence 1 | Cane | 1626 | 1742 | 3.87 | Ingestive | Oral-Manual | Anterior |
| ‘SC good 4_2’ | Teninha | AF | Sequence 1 | Cane | 1780 | 1854 | 2.47 | Ingestive | Oral-Manual | Anterior |
| ‘SC good 4_2’ | Teninha | AF | Sequence 1 | Cane | 1921 | 1979 | 1.93 | Ingestive | Oral-Manual | Anterior |
| ‘SC good 4_2’ | Teninha | AF | Sequence 1 | Cane | 1990 | 2018 | 0.93 | Ingestive | Oral-Manual | Anterior |
| ‘SC good 4_2’ | Teninha | AF | Sequence 1 | Cane | 2037 | 2114 | 2.57 | Ingestive | Oral-Manual | Anterior |
| ‘SC good 4_2’ | Teninha | AF | Sequence 1 | Cane | 2153 | 2205 | 1.73 | Ingestive | Oral-Manual | Anterior |
| ‘SC good 4_2’ | Teninha | AF | Sequence 1 | Cane | 2224 | 2283 | 1.97 | Ingestive | Oral-Manual | Posterior |
| ‘SC good 5_1’ | Patricia | SAF | Sequence 1 | Cane | 151 | 225 | 2.47 | Ingestive | Oral | Anterior |
| ‘SC good 5_1’ | Patricia | SAF | Sequence 1 | Cane | 225 | 327 | 3.40 | Masticate |  |  |
| ‘SC good 5_1’ | Patricia | SAF | Sequence 1 | Cane | 327 | 346 | 0.63 | Ingestive | Oral | Anterior |
| ‘SC good 5_1’ | Patricia | SAF | Sequence 1 | Cane | 397 | 427 | 1.00 | Ingestive | Oral | Anterior |
| ‘SC good 5_1’ | Patricia | SAF | Sequence 1 | Cane | 442 | 450 | 0.27 | Ingestive | Oral | Anterior |
| ‘SC good 5_1’ | Patricia | SAF | Sequence 1 | Cane | 450 | 530 | 2.67 | Ingestive | Oral-Manual | Posterior |
| ‘SC good 5_1’ | Patricia | SAF | Sequence 1 | Cane | 530 | 631 | 3.37 | Masticate |  |  |
| ‘SC good 5_1’ | Patricia | SAF | Sequence 1 | Cane | 631 | 697 | 2.20 | Ingestive | Oral | Anterior |
| ‘SC good 5_1’ | Patricia | SAF | Sequence 1 | Cane | 781 | 818 | 1.23 | Ingestive | Oral | Anterior |
| ‘SC good 5_1’ | Patricia | SAF | Sequence 1 | Cane | 818 | 1020 | 6.73 | Masticate |  |  |
| ‘SC good 5_1’ | Patricia | SAF | Sequence 1 | Cane | 1020 | 1125 | 3.50 | Ingestive | Oral | Anterior |
| ‘SC good 5_1’ | Patricia | SAF | Sequence 1 | Cane | 1153 | 1174 | 0.70 | Ingestive | Oral | Anterior |
| ‘SC good 5_1’ | Patricia | SAF | Sequence 1 | Cane | 1209 | 1298 | 2.97 | Ingestive | Oral-Manual | Posterior |
| ‘SC good 5_1’ | Patricia | SAF | Sequence 1 | Cane | 1303 | 1322 | 0.63 | Ingestive | Oral | Anterior |
| ‘SC good 5_1’ | Patricia | SAF | Sequence 1 | Cane | 1322 | 1386 | 2.13 | Masticate |  |  |
| ‘SC good 5_1’ | Patricia | SAF | Sequence 1 | Cane | 1386 | 1402 | 0.53 | Ingestive | Oral | Anterior |
| ‘SC good 5_1’ | Patricia | SAF | Sequence 1 | Cane | 1402 | 1452 | 1.67 | Masticate |  |  |
| ‘SC good 5_1’ | Patricia | SAF | Sequence 1 | Cane | 1452 | 1639 | 6.23 | Ingestive | Oral | Anterior |
| ‘SC good 5_2’ | Patricia | SAF | Sequence 1 | Cane | 18 | 153 | 4.50 | Ingestive | Oral-Manual | Posterior |
| ‘SC good 5_2’ | Patricia | SAF | Sequence 1 | Cane | 153 | 222 | 2.30 | Masticate |  |  |
| ‘SC good 5_2’ | Patricia | SAF | Sequence 1 | Cane | 272 | 322 | 1.67 | Ingestive | Oral-Manual | Posterior |
| ‘SC good 5_2’ | Patricia | SAF | Sequence 1 | Cane | 322 | 405 | 2.77 | Masticate |  |  |
| ‘SC good 5_2’ | Patricia | SAF | Sequence 1 | Cane | 434 | 519 | 2.83 | Ingestive | Oral-Manual | Posterior |
| ‘SC good 5_2’ | Patricia | SAF | Sequence 1 | Cane | 559 | 624 | 2.17 | Ingestive | Oral-Manual | Posterior |
| ‘SC good 5_2’ | Patricia | SAF | Sequence 1 | Cane | 624 | 837 | 7.10 | Masticate |  |  |
| ‘SC good chews 6’ | Catu | AM | Sequence 1 | Cane | 1 | 106 | 3.50 | Masticate |  |  |
| ‘SC good chews 6’ | Catu | AM | Sequence 1 | Cane | 106 | 123 | 0.57 | Ingestive | Oral-Manual | Anterior |
| ‘SC good chews 6’ | Catu | AM | Sequence 1 | Cane | 123 | 187 | 2.13 | Ingestive | Oral-Manual | Posterior |
| ‘SC good chews 6’ | Catu | AM | Sequence 1 | Cane | 210 | 236 | 0.87 | Ingestive | Oral-Manual | Anterior |
| ‘SC good chews 6’ | Catu | AM | Sequence 1 | Cane | 236 | 318 | 2.73 | Masticate |  |  |
| ‘SC good chews 6’ | Catu | AM | Sequence 1 | Cane | 366 | 426 | 2.00 | Ingestive | Oral-Manual | Posterior |
| ‘SC good chews 6’ | Catu | AM | Sequence 1 | Cane | 426 | 469 | 1.43 | Masticate |  |  |
| ‘SC good chews 6’ | Catu | AM | Sequence 1 | Cane | 469 | 534 | 2.17 | Ingestive | Oral-Manual | Posterior |
| ‘SC good chews 6’ | Catu | AM | Sequence 1 | Cane | 685 | 697 | 0.40 | Ingestive | Oral-Manual | Anterior |
| ‘SC good chews 6’ | Catu | AM | Sequence 1 | Cane | 697 | 808 | 3.70 | Ingestive | Oral-Manual | Posterior |
| ‘SC good chews 6’ | Catu | AM | Sequence 1 | Cane | 886 | 974 | 2.93 | Ingestive | Oral-Manual | Posterior |
| ‘SC good chews 6’ | Catu | AM | Sequence 1 | Cane | 1030 | 1066 | 1.20 | Ingestive | Oral-Manual | Anterior |
| ‘SC good chews 6’ | Catu | AM | Sequence 1 | Cane | 1085 | 1137 | 1.73 | Ingestive | Oral-Manual | Posterior |
| ‘SC good_1’ | Tais | SAF | Sequence 1 | Cane | 1 | 29 | 0.93 | Ingestive | Oral-Manual | Posterior |
| ‘SC good_1’ | Tais | SAF | Sequence 1 | Cane | 59 | 94 | 1.17 | Ingestive | Oral | Anterior |
| ‘SC good_1’ | Tais | SAF | Sequence 1 | Cane | 119 | 149 | 1.00 | Ingestive | Oral-Manual | Posterior |
| ‘SC good_1’ | Tais | SAF | Sequence 1 | Cane | 149 | 185 | 1.20 | Masticate |  |  |
| ‘SC good_1’ | Tais | SAF | Sequence 1 | Cane | 201 | 289 | 2.93 | Ingestive | Oral | Anterior |
| ‘SC good_1’ | Tais | SAF | Sequence 1 | Cane | 305 | 395 | 3.00 | Ingestive | Oral-Manual | Posterior |
| ‘SC good_1’ | Tais | SAF | Sequence 1 | Cane | 436 | 532 | 3.20 | Ingestive | Oral-Manual | Posterior |
| ‘SC good_1’ | Tais | SAF | Sequence 1 | Cane | 577 | 677 | 3.33 | Ingestive | Oral-Manual | Posterior |
| ‘SC good_1’ | Tais | SAF | Sequence 1 | Cane | 714 | 728 | 0.47 | Ingestive | Oral | Anterior |
| ‘SC good_1’ | Tais | SAF | Sequence 1 | Cane | 751 | 765 | 0.47 | Ingestive | Oral | Anterior |
| ‘SC good_1’ | Tais | SAF | Sequence 1 | Cane | 791 | 816 | 0.83 | Ingestive | Oral-Manual | Posterior |
| ‘SC good_1’ | Tais | SAF | Sequence 1 | Cane | 834 | 869 | 1.17 | Ingestive | Oral-Manual | Anterior |
| ‘SC good_1’ | Tais | SAF | Sequence 1 | Cane | 869 | 963 | 3.13 | Ingestive | Oral-Manual | Posterior |
| ‘SC good_1’ | Tais | SAF | Sequence 1 | Cane | 996 | 1012 | 0.53 | Ingestive | Oral | Anterior |
| ‘SC good_1’ | Tais | SAF | Sequence 1 | Cane | 1079 | 1089 | 0.33 | Ingestive | Oral | Anterior |
| ‘SC good_1’ | Tais | SAF | Sequence 1 | Cane | 1169 | 1198 | 0.97 | Ingestive | Oral | Anterior |
| ‘SC good_1’ | Tais | SAF | Sequence 1 | Cane | 1213 | 1235 | 0.73 | Ingestive | Oral | Anterior |
| ‘SC good_1’ | Tais | SAF | Sequence 1 | Cane | 1257 | 1284 | 0.90 | Ingestive | Oral | Anterior |
| ‘SC good_1’ | Tais | SAF | Sequence 1 | Cane | 1284 | 1388 | 3.47 | Ingestive | Oral-Manual | Posterior |
| ‘SC good_1’ | Tais | SAF | Sequence 1 | Cane | 1388 | 1440 | 1.73 | Masticate |  |  |
| ‘SC good_1’ | Tais | SAF | Sequence 1 | Cane | 1440 | 1522 | 2.73 | Ingestive | Oral | Anterior |
| ‘SC good_1’ | Tais | SAF | Sequence 1 | Cane | 1522 | 1595 | 2.43 | Ingestive | Oral-Manual | Posterior |
| ‘SC good_1’ | Tais | SAF | Sequence 1 | Cane | 1613 | 1695 | 2.73 | Ingestive | Oral-Manual | Posterior |
| ‘SC good_1’ | Tais | SAF | Sequence 1 | Cane | 1716 | 1742 | 0.87 | Ingestive | Oral | Anterior |
| ‘SC good_2’ | Tais | SAF | Sequence 1 | Cane | 1 | 31 | 1.00 | Ingestive | Oral-Manual | Posterior |
| ‘SC good_2’ | Tais | SAF | Sequence 1 | Cane | 136 | 198 | 2.07 | Ingestive | Oral | Anterior |
| ‘SC good_2’ | Tais | SAF | Sequence 1 | Cane | 241 | 276 | 1.17 | Ingestive | Oral | Anterior |
| ‘SC good_2’ | Tais | SAF | Sequence 1 | Cane | 292 | 312 | 0.67 | Ingestive | Oral | Anterior |
| ‘SC good_2’ | Tais | SAF | Sequence 1 | Cane | 404 | 426 | 0.73 | Ingestive | Oral | Anterior |
| ‘SC good_2’ | Tais | SAF | Sequence 1 | Cane | 470 | 498 | 0.93 | Ingestive | Oral | Anterior |
| ‘SC good_2’ | Tais | SAF | Sequence 1 | Cane | 635 | 686 | 1.70 | Ingestive | Oral | Anterior |
| ‘SC good_2’ | Tais | SAF | Sequence 1 | Cane | 721 | 739 | 0.60 | Ingestive | Oral | Anterior |
| ‘SC good_2’ | Tais | SAF | Sequence 1 | Cane | 768 | 852 | 2.80 | Ingestive | Oral-Manual | Posterior |
| ‘SC good_2’ | Tais | SAF | Sequence 1 | Cane | 687 | 1009 | 10.73 | Ingestive | Oral-Manual | Posterior |
| ‘SC good_2’ | Tais | SAF | Sequence 1 | Cane | 1032 | 1130 | 3.27 | Ingestive | Oral-Manual | Posterior |
| ‘SC good_2’ | Tais | SAF | Sequence 1 | Cane | 1172 | 1207 | 1.17 | Ingestive | Oral | Anterior |
| ‘SC good_2’ | Tais | SAF | Sequence 1 | Cane | 1228 | 1328 | 3.33 | Ingestive | Oral-Manual | Posterior |
| ‘SC good_2’ | Tais | SAF | Sequence 1 | Cane | 1356 | 1454 | 3.27 | Ingestive | Oral-Manual | Posterior |
| ‘SC good_2’ | Tais | SAF | Sequence 1 | Cane | 1478 | 1513 | 1.17 | Ingestive | Oral | Anterior |
| ‘SC good_2’ | Tais | SAF | Sequence 1 | Cane | 1532 | 1541 | 0.30 | Ingestive | Oral-Manual | Posterior |
| ‘SC good_2’ | Tais | SAF | Sequence 1 | Cane | 1570 | 1657 | 2.90 | Ingestive | Oral-Manual | Posterior |
| ‘SC good_2’ | Tais | SAF | Sequence 1 | Cane | 1685 | 1697 | 0.40 | Ingestive | Oral | Anterior |
| ‘SC good_2’ | Tais | SAF | Sequence 1 | Cane | 1697 | 1764 | 2.23 | Masticate |  |  |
| ‘SC good_2’ | Tais | SAF | Sequence 1 | Cane | 1764 | 1798 | 1.13 | Ingestive | Oral | Anterior |
| ‘SC good_2’ | Tais | SAF | Sequence 1 | Cane | 1798 | 1821 | 0.77 | Ingestive | Oral-Manual | Posterior |
| ‘SC good_2’ | Tais | SAF | Sequence 1 | Cane | 1875 | 1885 | 0.33 | Ingestive | Oral | Anterior |
| ‘SC good_2’ | Tais | SAF | Sequence 1 | Cane | 1893 | 1972 | 2.63 | Ingestive | Oral-Manual | Posterior |
| ‘SC good_2’ | Tais | SAF | Sequence 1 | Cane | 1972 | 2052 | 2.67 | Masticate |  |  |
| ‘SC good_2’ | Tais | SAF | Sequence 1 | Cane | 2052 | 2075 | 0.77 | Ingestive | Oral | Anterior |
| ‘SC good_2’ | Tais | SAF | Sequence 1 | Cane | 2084 | 2163 | 2.63 | Ingestive | Oral-Manual | Posterior |
| ‘SC good_2’ | Tais | SAF | Sequence 1 | Cane | 2184 | 2283 | 3.30 | Ingestive | Oral | Anterior |
| ‘SC good_2’ | Tais | SAF | Sequence 1 | Cane | 2292 | 2384 | 3.07 | Ingestive | Oral | Anterior |
| ‘SC good_2’ | Tais | SAF | Sequence 1 | Cane | 2479 | 2507 | 0.93 | Ingestive | Oral | Anterior |
| ‘SC good_2’ | Tais | SAF | Sequence 1 | Cane | 2539 | 2564 | 0.83 | Ingestive | Oral | Anterior |
| ‘SC good_2’ | Tais | SAF | Sequence 1 | Cane | 2638 | 2671 | 1.10 | Ingestive | Oral | Anterior |
| ‘SC good_2’ | Tais | SAF | Sequence 1 | Cane | 2693 | 2775 | 2.73 | Ingestive | Oral-Manual | Posterior |
| ‘SC good_2’ | Tais | SAF | Sequence 1 | Cane | 2811 | 2940 | 4.30 | Ingestive | Oral-Manual | Posterior |
| ‘SC good_2’ | Tais | SAF | Sequence 1 | Cane | 2968 | 3061 | 3.10 | Ingestive | Oral-Manual | Posterior |
| ‘SC good_2’ | Tais | SAF | Sequence 1 | Cane | 3087 | 3155 | 2.27 | Ingestive | Oral-Manual | Posterior |
| ‘SC good_2’ | Tais | SAF | Sequence 1 | Cane | 3178 | 3239 | 2.03 | Ingestive | Oral-Manual | Posterior |
| ‘SC good_2’ | Tais | SAF | Sequence 1 | Cane | 3282 | 3305 | 0.77 | Ingestive | Oral | Anterior |
| ‘SC good_2’ | Tais | SAF | Sequence 1 | Cane | 3317 | 3432 | 3.83 | Ingestive | Oral-Manual | Posterior |
| ‘SC good_2’ | Tais | SAF | Sequence 1 | Cane | 3496 | 3517 | 0.70 | Ingestive | Oral | Anterior |
| ‘SC good_2’ | Tais | SAF | Sequence 1 | Cane | 3568 | 3600 | 1.07 | Ingestive | Oral | Anterior |
| ‘SC good_2’ | Tais | SAF | Sequence 1 | Cane | 3624 | 3663 | 1.30 | Ingestive | Oral-Manual | Posterior |
| ‘SC good_2’ | Tais | SAF | Sequence 1 | Cane | 3663 | 3766 | 3.43 | Masticate |  |  |
| ‘SC good_2’ | Tais | SAF | Sequence 1 | Cane | 3785 | 3800 | 0.50 | Ingestive | Oral | Anterior |
| ‘SC good_2’ | Tais | SAF | Sequence 1 | Cane | 3813 | 3855 | 1.40 | Ingestive | Oral-Manual | Posterior |
| ‘SC good_2’ | Tais | SAF | Sequence 1 | Cane | 3855 | 3961 | 3.53 | Masticate |  |  |
| ‘SC good_2’ | Tais | SAF | Sequence 1 | Cane | 3979 | 4003 | 0.80 | Ingestive | Oral | Anterior |
| ‘SC good_2’ | Tais | SAF | Sequence 1 | Cane | 4026 | 4175 | 4.97 | Ingestive | Oral-Manual | Posterior |
| ‘SC good_2’ | Tais | SAF | Sequence 1 | Cane | 4175 | 4211 | 1.20 | Masticate |  |  |
| ‘SC good_2’ | Tais | SAF | Sequence 1 | Cane | 4211 | 4261 | 1.67 | Ingestive | Oral-Manual | Posterior |
| ‘SC good_2’ | Tais | SAF | Sequence 1 | Cane | 4279 | 4430 | 5.03 | Ingestive | Oral | Anterior |
| ‘SC good1_1’ | Tais | SAF | Sequence 1 | Cane | 9 | 12 | 0.10 | Ingestive | Oral-Manual | Anterior |
| ‘SC good1_1’ | Tais | SAF | Sequence 1 | Cane | 12 | 74 | 2.07 | Masticate |  |  |
| ‘SC good1_2’ | Tais | SAF | Sequence 1 | Cane | 4 | 27 | 0.77 | Ingestive | Oral | Anterior |
| ‘SC good1_2’ | Tais | SAF | Sequence 1 | Cane | 53 | 114 | 2.03 | Ingestive | Oral-Manual | Posterior |
| ‘SC good1_2’ | Tais | SAF | Sequence 1 | Cane | 143 | 177 | 1.13 | Ingestive | Oral | Anterior |
| ‘SC good1_2’ | Tais | SAF | Sequence 1 | Cane | 210 | 230 | 0.67 | Ingestive | Oral-Manual | Posterior |
| ‘SC good1_2’ | Tais | SAF | Sequence 1 | Cane | 230 | 312 | 2.73 | Masticate |  |  |
| ‘SC good1_2’ | Tais | SAF | Sequence 1 | Cane | 329 | 360 | 1.03 | Ingestive | Oral | Anterior |
| ‘SC good1_2’ | Tais | SAF | Sequence 1 | Cane | 400 | 504 | 3.47 | Ingestive | Oral-Manual | Posterior |
| ‘SC good1_2’ | Tais | SAF | Sequence 1 | Cane | 564 | 615 | 1.70 | Ingestive | Oral | Anterior |
| ‘SC good1_2’ | Tais | SAF | Sequence 1 | Cane | 632 | 700 | 2.27 | Ingestive | Oral-Manual | Posterior |
| ‘SC good1_2’ | Tais | SAF | Sequence 1 | Cane | 724 | 806 | 2.73 | Ingestive | Oral-Manual | Posterior |
| ‘SC good1_2’ | Tais | SAF | Sequence 1 | Cane | 837 | 859 | 0.73 | Ingestive | Oral | Anterior |
| ‘SC good1_2’ | Tais | SAF | Sequence 1 | Cane | 868 | 918 | 1.67 | Ingestive | Oral-Manual | Posterior |
| ‘SC good1_2’ | Tais | SAF | Sequence 1 | Cane | 918 | 1021 | 3.43 | Masticate |  |  |
| ‘SC good1_2’ | Tais | SAF | Sequence 1 | Cane | 1021 | 1043 | 0.73 | Ingestive | Oral | Anterior |
| ‘SC good1_2’ | Tais | SAF | Sequence 1 | Cane | 1055 | 1090 | 1.17 | Ingestive | Oral-Manual | Posterior |
| ‘SC good1_2’ | Tais | SAF | Sequence 1 | Cane | 1090 | 1183 | 3.10 | Masticate |  |  |
| ‘SC good1_2’ | Tais | SAF | Sequence 1 | Cane | 1183 | 1254 | 2.37 | Ingestive | Oral | Anterior |
| ‘SC good1_2’ | Tais | SAF | Sequence 1 | Cane | 1291 | 1314 | 0.77 | Ingestive | Oral | Anterior |
| ‘SC good1_2’ | Tais | SAF | Sequence 1 | Cane | 1400 | 1425 | 0.83 | Ingestive | Oral | Anterior |
| ‘SC good1_2’ | Tais | SAF | Sequence 1 | Cane | 1441 | 1475 | 1.13 | Ingestive | Oral | Anterior |
| ‘SC good1_2’ | Tais | SAF | Sequence 1 | Cane | 1475 | 1589 | 3.80 | Masticate |  |  |
| ‘SC good1_2’ | Tais | SAF | Sequence 1 | Cane | 1589 | 1620 | 1.03 | Ingestive | Oral | Anterior |
| ‘SC good1_2’ | Tais | SAF | Sequence 1 | Cane | 1638 | 1738 | 3.33 | Ingestive | Oral-Manual | Posterior |
| ‘SC good1_2’ | Tais | SAF | Sequence 1 | Cane | 1738 | 1792 | 1.80 | Masticate |  |  |
| ‘SC good1_2’ | Tais | SAF | Sequence 1 | Cane | 1824 | 1874 | 1.67 | Ingestive | Oral | Anterior |
| ‘SC good1_2’ | Tais | SAF | Sequence 1 | Cane | 1892 | 1916 | 0.80 | Ingestive | Oral | Anterior |
| ‘SC good1_2’ | Tais | SAF | Sequence 1 | Cane | 1916 | 1984 | 2.27 | Masticate |  |  |
| ‘SC good1_2’ | Tais | SAF | Sequence 1 | Cane | 2001 | 2025 | 0.80 | Ingestive | Oral | Anterior |
| ‘SC good1_2’ | Tais | SAF | Sequence 1 | Cane | 2025 | 2131 | 3.53 | Masticate |  |  |
| ‘SC good1_2’ | Tais | SAF | Sequence 1 | Cane | 2131 | 2197 | 2.20 | Ingestive | Oral | Anterior |
| ‘SC good1_2’ | Tais | SAF | Sequence 1 | Cane | 2220 | 2256 | 1.20 | Ingestive | Oral | Anterior |
| ‘SC good1_2’ | Tais | SAF | Sequence 1 | Cane | 2256 | 2321 | 2.17 | Masticate |  |  |
| ‘SC good1_2’ | Tais | SAF | Sequence 1 | Cane | 2336 | 2363 | 0.90 | Ingestive | Oral | Anterior |
| ‘SC good1_2’ | Tais | SAF | Sequence 1 | Cane | 2428 | 2456 | 0.93 | Ingestive | Oral | Anterior |
| ‘SC good1_2’ | Tais | SAF | Sequence 1 | Cane | 2622 | 2657 | 1.17 | Ingestive | Oral | Anterior |
| ‘SC good1_2’ | Tais | SAF | Sequence 1 | Cane | 2676 | 2698 | 0.73 | Ingestive | Oral | Anterior |
| ‘SC good1_2’ | Tais | SAF | Sequence 1 | Cane | 2723 | 2800 | 2.57 | Ingestive | Oral | Anterior |
| ‘SC good1_2’ | Tais | SAF | Sequence 1 | Cane | 2808 | 2851 | 1.43 | Ingestive | Oral-Manual | Posterior |
| ‘SC good1_2’ | Tais | SAF | Sequence 1 | Cane | 2877 | 3008 | 4.37 | Ingestive | Oral-Manual | Posterior |
| ‘SC good1_2’ | Tais | SAF | Sequence 1 | Cane | 3021 | 3044 | 0.77 | Ingestive | Oral | Anterior |
| ‘SC good1_2’ | Tais | SAF | Sequence 1 | Cane | 3067 | 3113 | 1.53 | Ingestive | Oral-Manual | Posterior |
| ‘SC good1_2’ | Tais | SAF | Sequence 1 | Cane | 3133 | 3156 | 0.77 | Ingestive | Oral | Anterior |
| ‘SC good1_2’ | Tais | SAF | Sequence 1 | Cane | 3216 | 3225 | 0.30 | Ingestive | Oral | Anterior |
| ‘SC good1_2’ | Tais | SAF | Sequence 1 | Cane | 3297 | 3304 | 0.23 | Ingestive | Oral | Anterior |
| ‘SC good1_2’ | Tais | SAF | Sequence 1 | Cane | 3333 | 3350 | 0.57 | Ingestive | Oral | Anterior |
| ‘SC good1_2’ | Tais | SAF | Sequence 1 | Cane | 3359 | 3399 | 1.33 | Ingestive | Oral-Manual | Posterior |
| ‘SC good1_2’ | Tais | SAF | Sequence 1 | Cane | 3399 | 3513 | 3.80 | Masticate |  |  |
| ‘sloping anvil_3’ | Chuchu | AF | Sequence 1 | Piaçava | 157 | 236 | 2.63 | Manual |  |  |
| ‘sloping anvil_3’ | Chuchu | AF | Sequence 1 | Piaçava | 344 | 452 | 3.60 | Manual |  |  |
| ‘sloping anvil_4’ | Chuchu | AF | Sequence 1 | Piaçava | 8 | 77 | 2.30 | Manual |  |  |
| ‘sloping anvil_4’ | Chuchu | AF | Sequence 1 | Piaçava | 210 | 259 | 1.63 | Manual |  |  |
| ‘sloping anvil_4’ | Chuchu | AF | Sequence 1 | Piaçava | 259 | 299 | 1.33 | Ingestive | Oral-Manual | Anterior |
| ‘sloping anvil_4’ | Chuchu | AF | Sequence 1 | Piaçava | 347 | 354 | 0.23 | Ingestive | Oral-Manual | Anterior |
| ‘sloping anvil_4’ | Chuchu | AF | Sequence 1 | Piaçava | 354 | 492 | 4.60 | Masticate |  |  |
| ‘sloping anvil_4’ | Chuchu | AF | Sequence 1 | Piaçava | 492 | 545 | 1.77 | Ingestive | Oral-Manual | Anterior |
| ‘sloping anvil_4’ | Chuchu | AF | Sequence 1 | Piaçava | 617 | 620 | 0.10 | Ingestive | Oral-Manual | Anterior |
| ‘sloping anvil_4’ | Chuchu | AF | Sequence 1 | Piaçava | 620 | 715 | 3.17 | Masticate |  |  |
| ‘sloping anvil_4’ | Chuchu | AF | Sequence 1 | Piaçava | 715 | 737 | 0.73 | Ingestive | Oral-Manual | Posterior |
| ‘sloping anvil_7’ | Chuchu | AF | Sequence 1 | Piaçava | 124 | 178 | 1.80 | Manual |  |  |
| ‘sloping anvil_7’ | Chuchu | AF | Sequence 1 | Piaçava | 178 | 306 | 4.27 | Manual |  |  |
| ‘sloping anvil_7’ | Chuchu | AF | Sequence 1 | Piaçava | 306 | 329 | 0.77 | Ingestive | Oral-Manual | Anterior |
| ‘sloping anvil_7’ | Chuchu | AF | Sequence 1 | Piaçava | 402 | 460 | 1.93 | Ingestive | Oral-Manual | Anterior |
| ‘still more m de boi_1’ | Coco | SAM | Sequence 1 | tucum | 1 | 92 | 3.03 | Ingestive | Oral-Manual | Anterior |
| ‘still more m de boi_1’ | Coco | SAM | Sequence 1 | tucum | 92 | 121 | 0.97 | Masticate |  |  |
| ‘still more m de boi_1’ | Coco | SAM | Sequence 1 | tucum | 121 | 144 | 0.77 | Ingestive | Oral-Manual | Anterior |
| ‘still more m de boi_1’ | Coco | SAM | Sequence 1 | tucum | 198 | 226 | 0.93 | Ingestive | Oral-Manual | Anterior |
| ‘still more m de boi_1’ | Coco | SAM | Sequence 1 | tucum | 261 | 307 | 1.53 | Manual |  |  |
| ‘still more m de boi_1’ | Coco | SAM | Sequence 1 | tucum | 307 | 313 | 0.20 | Ingestive | Oral-Manual | Anterior |
| ‘still more m de boi_1’ | Coco | SAM | Sequence 1 | tucum | 313 | 414 | 3.37 | Masticate |  |  |
| ‘still more m de boi_1’ | Coco | SAM | Sequence 1 | tucum | 414 | 421 | 0.23 | Ingestive | Oral-Manual | Anterior |
| ‘still more m de boi_1’ | Coco | SAM | Sequence 1 | tucum | 421 | 444 | 0.77 | Masticate |  |  |
| ‘still more m de boi_1’ | Coco | SAM | Sequence 1 | tucum | 444 | 463 | 0.63 | Ingestive | Oral-Manual | Anterior |
| ‘still more m de boi_1’ | Coco | SAM | Sequence 1 | tucum | 463 | 495 | 1.07 | Masticate |  |  |
| ‘still more m de boi_1’ | Coco | SAM | Sequence 1 | tucum | 495 | 534 | 1.30 | Ingestive | Oral-Manual | Anterior |
| ‘still more m de boi_1’ | Coco | SAM | Sequence 1 | tucum | 534 | 553 | 0.63 | Masticate |  |  |
| ‘still more m de boi_1’ | Coco | SAM | Sequence 1 | tucum | 553 | 559 | 0.20 | Ingestive | Oral-Manual | Anterior |
| ‘still more m de boi_1’ | Coco | SAM | Sequence 1 | tucum | 559 | 575 | 0.53 | Masticate |  |  |
| ‘still more m de boi_1’ | Coco | SAM | Sequence 1 | tucum | 575 | 593 | 0.60 | Ingestive | Oral-Manual | Anterior |
| ‘still more m de boi_1’ | Coco | SAM | Sequence 1 | tucum | 593 | 626 | 1.10 | Masticate |  |  |
| ‘still more m de boi_1’ | Coco | SAM | Sequence 1 | tucum | 626 | 647 | 0.70 | Ingestive | Oral-Manual | Anterior |
| ‘still more m de boi_1’ | Coco | SAM | Sequence 1 | tucum | 647 | 703 | 1.87 | Masticate |  |  |
| ‘still more m de boi_1’ | Coco | SAM | Sequence 1 | tucum | 703 | 724 | 0.70 | Ingestive | Oral-Manual | Anterior |
| ‘still more m de boi_1’ | Coco | SAM | Sequence 1 | tucum | 724 | 756 | 1.07 | Masticate |  |  |
| ‘still more m de boi_1’ | Coco | SAM | Sequence 1 | tucum | 756 | 785 | 0.97 | Ingestive | Oral-Manual | Anterior |
| ‘still more m de boi_1’ | Coco | SAM | Sequence 1 | tucum | 785 | 828 | 1.43 | Masticate |  |  |
| ‘still more m de boi_1’ | Coco | SAM | Sequence 1 | tucum | 828 | 850 | 0.73 | Ingestive | Oral-Manual | Anterior |
| ‘still more m de boi_1’ | Coco | SAM | Sequence 1 | tucum | 850 | 876 | 0.87 | Masticate |  |  |
| ‘still more m de boi_1’ | Coco | SAM | Sequence 1 | tucum | 876 | 886 | 0.33 | Ingestive | Oral-Manual | Anterior |
| ‘still more m de boi_1’ | Coco | SAM | Sequence 1 | tucum | 886 | 908 | 0.73 | Masticate |  |  |
| ‘still more m de boi_1’ | Coco | SAM | Sequence 1 | tucum | 908 | 927 | 0.63 | Ingestive | Oral-Manual | Anterior |
| ‘still more m de boi_1’ | Coco | SAM | Sequence 1 | tucum | 927 | 952 | 0.83 | Masticate |  |  |
| ‘still more m de boi_1’ | Coco | SAM | Sequence 1 | tucum | 952 | 964 | 0.40 | Ingestive | Oral-Manual | Anterior |
| ‘still more m de boi_1’ | Coco | SAM | Sequence 1 | tucum | 983 | 1006 | 0.77 | Ingestive | Oral-Manual | Anterior |
| ‘still more m de boi_1’ | Coco | SAM | Sequence 1 | tucum | 1027 | 1050 | 0.77 | Ingestive | Oral-Manual | Anterior |
| ‘still more m de boi_1’ | Coco | SAM | Sequence 1 | tucum | 1050 | 1075 | 0.83 | Masticate |  |  |
| ‘still more m de boi_1’ | Coco | SAM | Sequence 1 | tucum | 1075 | 1121 | 1.53 | Ingestive | Oral-Manual | Anterior |
| ‘still more m de boi_2’ | Coco | SAM | Sequence 1 | tucum | 19 | 26 | 0.23 | Ingestive | Oral-Manual | Anterior |
| ‘still more m de boi_2’ | Coco | SAM | Sequence 1 | tucum | 47 | 67 | 0.67 | Ingestive | Oral-Manual | Anterior |
| ‘still more m de boi_2’ | Coco | SAM | Sequence 1 | tucum | 83 | 90 | 0.23 | Ingestive | Oral-Manual | Anterior |
| ‘still more m de boi_2’ | Coco | SAM | Sequence 1 | tucum | 124 | 134 | 0.33 | Ingestive | Oral-Manual | Anterior |
| ‘still more m de boi_2’ | Coco | SAM | Sequence 1 | tucum | 158 | 167 | 0.30 | Ingestive | Oral-Manual | Anterior |
| ‘still more m de boi_2’ | Coco | SAM | Sequence 1 | tucum | 187 | 192 | 0.17 | Ingestive | Oral-Manual | Anterior |
| ‘Tiese_tuber_spiderhead_2’ | Tais | SAF | Sequence 1 | USO | 117 | 157 | 1.33 | Ingestive | Oral-Manual | Anterior |
| ‘Tiese_tuber_spiderhead_2’ | Tais | SAF | Sequence 1 | USO | 157 | 307 | 5.00 | Ingestive | Oral-Manual | Posterior |
| ‘Tiese_tuber_spiderhead_2’ | Tais | SAF | Sequence 1 | USO | 307 | 429 | 4.07 | Masticate |  |  |
| ‘Tiese_tuber_spiderhead_2’ | Tais | SAF | Sequence 1 | USO | 429 | 452 | 0.77 | Ingestive | Oral-Manual | Anterior |
| ‘Tiese_tuber_spiderhead_2’ | Tais | SAF | Sequence 1 | USO | 487 | 519 | 1.07 | Ingestive | Oral-Manual | Anterior |
| ‘Tiese_tuber_spiderhead_2’ | Tais | SAF | Sequence 1 | USO | 519 | 638 | 3.97 | Ingestive | Oral-Manual | Posterior |
| ‘Tiese_tuber_spiderhead_2’ | Tais | SAF | Sequence 1 | USO | 638 | 687 | 1.63 | Ingestive | Oral-Manual | Posterior |
| ‘Tiese_tuber_spiderhead_2’ | Tais | SAF | Sequence 1 | USO | 687 | 776 | 2.97 | Masticate |  |  |
| ‘Tiese_tuber_spiderhead_2’ | Tais | SAF | Sequence 1 | USO | 776 | 991 | 7.17 | Ingestive | Oral-Manual | Posterior |
| ‘Tiese_tuber_spiderhead_3’ | Tais | SAF | Sequence 1 | USO | 1 | 51 | 1.67 | Ingestive | Oral-Manual | Anterior |
| ‘Tiese_tuber_spiderhead_3’ | Tais | SAF | Sequence 1 | USO | 87 | 248 | 5.37 | Ingestive | Oral-Manual | Posterior |
| ‘tiny bananas very good’ | Pacoca | AF | Sequence 1 | Banana | 113 | 302 | 6.30 | Masticate |  |  |
| ‘tiny bananas very good’ | Pacoca | AF | Sequence 1 | Banana | 693 | 986 | 9.77 | Ingestive | Oral-Manual | Anterior |
| ‘tiny bananas very good’ | Pacoca | AF | Sequence 1 | Banana | 986 | 1161 | 5.83 | Masticate |  |  |
| ‘tiny bananas very good’ | Pacoca | AF | Sequence 1 | Banana | 1425 | 1428 | 0.10 | Ingestive | Oral-Manual | Anterior |
| ‘tiny bananas very good’ | Pacoca | AF | Sequence 1 | Banana | 1428 | 1615 | 6.23 | Masticate |  |  |
| ‘tiny bananas very good’ | Pacoca | AF | Sequence 1 | Banana | 1629 | 1633 | 0.13 | Ingestive | Oral-Manual | Anterior |
| ‘tiny bananas very good’ | Pacoca | AF | Sequence 1 | Banana | 2839 | 2845 | 0.20 | Ingestive | Oral-Manual | Anterior |
| ‘tiny bananas very good’ | Pacoca | AF | Sequence 1 | Banana | 4783 | 4791 | 0.27 | Ingestive | Oral-Manual | Anterior |
| ‘tiny bananas very good’ | Pacoca | AF | Sequence 1 | Banana | 5108 | 5134 | 0.87 | Ingestive | Oral-Manual | Anterior |
| ‘tiny bananas very good’ | Pacoca | AF | Sequence 1 | Banana | 5134 | 5473 | 11.30 | Masticate |  |  |
| ‘tiny bananas very good’ | Pacoca | AF | Sequence 1 | Banana | 5525 | 5528 | 0.10 | Ingestive | Oral-Manual | Anterior |
| ‘tiny bananas very good’ | Pacoca | AF | Sequence 1 | Banana | 6074 | 6085 | 0.37 | Ingestive | Oral-Manual | Anterior |
| ‘tiny bananas very good’ | Pacoca | AF | Sequence 1 | Banana | 6085 | 6171 | 2.87 | Masticate |  |  |
| ‘tiny bananas very good’ | Pacoca | AF | Sequence 1 | Banana | 6981 | 6990 | 0.30 | Ingestive | Oral-Manual | Anterior |
| ‘tiny bananas very good’ | Pacoca | AF | Sequence 1 | Banana | 6990 | 7174 | 6.13 | Masticate |  |  |
| ‘tiny bananas very good’ | Pacoca | AF | Sequence 1 | Banana | 7259 | 7262 | 0.10 | Ingestive | Oral-Manual | Anterior |
| ‘tiny bananas very good’ | Pacoca | AF | Sequence 1 | Banana | 7262 | 7559 | 9.90 | Masticate |  |  |
| ‘tiny bananas very good’ | Pacoca | AF | Sequence 1 | Banana | 7559 | 7602 | 1.43 | Ingestive | Oral-Manual | Anterior |
| ‘tiny bananas very good’ | Pacoca | AF | Sequence 1 | Banana | 7891 | 7899 | 0.27 | Masticate |  |  |
| ‘tiny bananas very good’ | Pacoca | AF | Sequence 1 | Banana | 1014 | 1016 | 0.07 | Ingestive | Oral-Manual | Anterior |
| ‘tiny bananas very good’ | Pacoca | AF | Sequence 1 | Banana | 1016 | 1030 | 0.47 | Masticate |  |  |
| ‘tiny bananas very good’ | Pacoca | AF | Sequence 1 | Banana | 1030 | 1031 | 0.03 | Ingestive | Oral-Manual | Anterior |
| ‘watermelon a beach sequence good_1’ | Doree | AF | Sequence 1 | Berries | 723 | 767 | 1.47 | Manual |  |  |
| ‘watermelon a beach sequence good_1’ | Doree | AF | Sequence 1 | Berries | 767 | 801 | 1.13 | Ingestive | Oral-Manual | Posterior |
| ‘watermelon a beach sequence good_1’ | Doree | AF | Sequence 1 | Berries | 801 | 833 | 1.07 | Masticate |  |  |
| ‘watermelon a beach sequence good_1’ | Doree | AF | Sequence 1 | Berries | 892 | 924 | 1.07 | Ingestive | Oral-Manual | Posterior |
| ‘watermelon a beach sequence good_1’ | Doree | AF | Sequence 1 | Berries | 924 | 1121 | 6.57 | Masticate |  |  |
| ‘watermelon a beach sequence good_1’ | Doree | AF | Sequence 1 | Berries | 1233 | 1290 | 1.90 | Ingestive | Oral-Manual | Posterior |
| ‘watermelon a beach sequence good_1’ | Doree | AF | Sequence 1 | Berries | 1290 | 1349 | 1.97 | Masticate |  |  |
| ‘watermelon a beach sequence good_1’ | Doree | AF | Sequence 1 | Berries | 1349 | 1441 | 3.07 | Manual |  |  |
| ‘watermelon a beach sequence good_1’ | Doree | AF | Sequence 1 | Berries | 1441 | 1496 | 1.83 | Ingestive | Oral-Manual | Posterior |
| ‘watermelon a beach sequence good_1’ | Doree | AF | Sequence 1 | Berries | 1496 | 1553 | 1.90 | Masticate |  |  |
| ‘watermelon a beach sequence good_1’ | Doree | AF | Sequence 1 | Berries | 1553 | 1572 | 0.63 | Ingestive | Oral-Manual | Posterior |
| ‘watermelon a beach sequence good_1’ | Doree | AF | Sequence 1 | Berries | 1572 | 1857 | 9.50 | Masticate |  |  |
| ‘watermelon a beach sequence good_1’ | Doree | AF | Sequence 1 | Berries | 1857 | 1890 | 1.10 | Ingestive | Oral-Manual | Posterior |
| ‘watermelon a beach sequence good_1’ | Doree | AF | Sequence 1 | Berries | 1923 | 1942 | 0.63 | Ingestive | Oral-Manual | Posterior |
| ‘watermelon a beach sequence good_1’ | Doree | AF | Sequence 1 | Berries | 1942 | 2054 | 3.73 | Masticate |  |  |
| ‘watermelon a beach sequence good_1’ | Doree | AF | Sequence 1 | Berries | 2088 | 2095 | 0.23 | Ingestive | Oral-Manual | Posterior |
| ‘watermelon a beach sequence good_1’ | Doree | AF | Sequence 1 | Berries | 2131 | 2160 | 0.97 | Ingestive | Oral-Manual | Posterior |
| ‘watermelon a beach sequence good_1’ | Doree | AF | Sequence 1 | Berries | 2160 | 2394 | 7.80 | Masticate |  |  |
| ‘watermelon a beach sequence good_1’ | Cenoura | AF | Sequence 1 | Berries | 2547 | 2565 | 0.60 | Ingestive | Oral-Manual | Posterior |
| ‘watermelon a beach sequence good_1’ | Cenoura | AF | Sequence 1 | Berries | 2621 | 2637 | 0.53 | Ingestive | Oral-Manual | Posterior |
| ‘watermelon a beach sequence good_1’ | Cenoura | AF | Sequence 1 | Berries | 2637 | 2675 | 1.27 | Masticate |  |  |
| ‘watermelon a beach sequence good_1’ | Cenoura | AF | Sequence 1 | Berries | 2675 | 2679 | 0.13 | Ingestive | Oral-Manual | Anterior |
| ‘watermelon a beach sequence good_1’ | Cenoura | AF | Sequence 1 | Berries | 2720 | 2796 | 2.53 | Ingestive | Oral-Manual | Posterior |
| ‘watermelon a beach sequence good_1’ | Cenoura | AF | Sequence 1 | Berries | 2796 | 2878 | 2.73 | Masticate |  |  |
| ‘watermelon a beach sequence good_1’ | Cenoura | AF | Sequence 1 | Berries | 2878 | 2949 | 2.37 | Ingestive | Oral-Manual | Posterior |
| ‘watermelon a beach sequence good_1’ | Cenoura | AF | Sequence 1 | Berries | 2949 | 3070 | 4.03 | Masticate |  |  |
| ‘watermelon a beach sequence good_1’ | Cenoura | AF | Sequence 1 | Berries | 3070 | 3097 | 0.90 | Ingestive | Oral-Manual | Posterior |
| ‘watermelon a beach sequence good_1’ | Cenoura | AF | Sequence 1 | Berries | 3322 | 3364 | 1.40 | Ingestive | Oral-Manual | Posterior |
| ‘watermelon a beach sequence good_1’ | Cenoura | AF | Sequence 1 | Berries | 3616 | 3850 | 7.80 | Manual |  |  |
| ‘watermelon a beach sequence good_1’ | Cenoura | AF | Sequence 1 | Berries | 3850 | 3865 | 0.50 | Ingestive | Oral-Manual | Posterior |
| ‘watermelon a beach sequence good_1’ | Cenoura | AF | Sequence 1 | Berries | 3887 | 3943 | 1.87 | Ingestive | Oral-Manual | Anterior |
| ‘watermelon a beach sequence good_1’ | Cenoura | AF | Sequence 1 | Berries | 3994 | 4020 | 0.87 | Ingestive | Oral-Manual | Posterior |
| ‘watermelon a beach sequence good_1’ | Cenoura | AF | Sequence 1 | Berries | 4059 | 4084 | 0.83 | Ingestive | Oral-Manual | Posterior |
| ‘watermelon a beach sequence good_1’ | Cenoura | AF | Sequence 1 | Berries | 4084 | 4168 | 2.80 | Masticate |  |  |
| ‘watermelon a beach sequence good_1’ | Cenoura | AF | Sequence 1 | Berries | 4168 | 4227 | 1.97 | Ingestive | Oral-Manual | Posterior |
| ‘watermelon a beach sequence good_1’ | Cenoura | AF | Sequence 1 | Berries | 4227 | 4328 | 3.37 | Masticate |  |  |
| ‘watermelon a beach sequence good_1’ | Cenoura | AF | Sequence 1 | Berries | 4328 | 4352 | 0.80 | Ingestive | Oral-Manual | Posterior |
| ‘watermelon a beach sequence good_1’ | Cenoura | AF | Sequence 1 | Berries | 4352 | 4481 | 4.30 | Masticate |  |  |
| ‘watermelon a beach sequence good_1’ | Cenoura | AF | Sequence 1 | Berries | 4481 | 4484 | 0.10 | Ingestive | Oral-Manual | Posterior |
| ‘watermelon a beach sequence good_1’ | Cenoura | AF | Sequence 1 | Berries | 4484 | 4534 | 1.67 | Masticate |  |  |
| ‘watermelon a beach sequence good_1’ | Cenoura | AF | Sequence 1 | Berries | 4534 | 4585 | 1.70 | Ingestive | Oral-Manual | Posterior |
| ‘watermelon a beach sequence good_1’ | Cenoura | AF | Sequence 1 | Berries | 4585 | 4769 | 6.13 | Masticate |  |  |
| ‘watermelon a beach sequence good_1’ | Cenoura | AF | Sequence 1 | Berries | 4795 | 4824 | 0.97 | Ingestive | Oral-Manual | Anterior |
| ‘watermelon a beach sequence good_1’ | Cenoura | AF | Sequence 1 | Berries | 4824 | 4862 | 1.27 | Masticate |  |  |
| ‘watermelon a beach sequence good_1’ | Cenoura | AF | Sequence 1 | Berries | 4862 | 4921 | 1.97 | Ingestive | Oral-Manual | Posterior |
| ‘watermelon a beach sequence good_1’ | Cenoura | AF | Sequence 1 | Berries | 4921 | 5041 | 4.00 | Masticate |  |  |
| ‘watermelon a beach sequence good_1’ | Cenoura | AF | Sequence 1 | Berries | 5097 | 5185 | 2.93 | Ingestive | Oral-Manual | Anterior |
| ‘watermelon a beach sequence good_1’ | Cenoura | AF | Sequence 1 | Berries | 5185 | 5744 | 18.63 | Manual |  |  |
| ‘watermelon a beach sequence good_1’ | Cenoura | AF | Sequence 1 | Berries | 5744 | 5761 | 0.57 | Ingestive | Oral-Manual | Anterior |
| ‘watermelon a beach sequence good_1’ | Cenoura | AF | Sequence 1 | Berries | 5824 | 5838 | 0.47 | Ingestive | Oral-Manual | Posterior |
| ‘watermelon a beach sequence good_1’ | Cenoura | AF | Sequence 1 | Berries | 5838 | 5905 | 2.23 | Masticate |  |  |
| ‘watermelon a beach sequence good_1’ | Cenoura | AF | Sequence 1 | Berries | 5905 | 5908 | 0.10 | Ingestive | Oral-Manual | Posterior |
| ‘watermelon a beach sequence good_1’ | Cenoura | AF | Sequence 1 | Berries | 5908 | 5953 | 1.50 | Masticate |  |  |
| ‘watermelon a beach sequence good_1’ | Cenoura | AF | Sequence 1 | Berries | 5953 | 5959 | 0.20 | Ingestive | Oral-Manual | Anterior |
| ‘watermelon a beach sequence good_1’ | Cenoura | AF | Sequence 1 | Berries | 5959 | 6013 | 1.80 | Masticate |  |  |
| ‘watermelon a beach sequence good_1’ | Cenoura | AF | Sequence 1 | Berries | 6013 | 6017 | 0.13 | Ingestive | Oral-Manual | Anterior |
| ‘watermelon a beach sequence good_1’ | Cenoura | AF | Sequence 1 | Berries | 6017 | 6076 | 1.97 | Masticate |  |  |
| ‘watermelon a beach sequence good_1’ | Cenoura | AF | Sequence 1 | Berries | 6076 | 6081 | 0.17 | Ingestive | Oral-Manual | Posterior |
| ‘watermelon a beach sequence good_1’ | Cenoura | AF | Sequence 1 | Berries | 6081 | 6127 | 1.53 | Masticate |  |  |
| ‘watermelon a beach sequence good_1’ | Cenoura | AF | Sequence 1 | Berries | 6127 | 6130 | 0.10 | Ingestive | Oral-Manual | Anterior |
| ‘watermelon a beach sequence good_1’ | Cenoura | AF | Sequence 1 | Berries | 6130 | 6262 | 4.40 | Masticate |  |  |
| ‘watermelon a beach sequence good_1’ | Cenoura | AF | Sequence 1 | Berries | 6413 | 6488 | 2.50 | Ingestive | Oral-Manual | Posterior |
| ‘watermelon a beach sequence good_1’ | Cenoura | AF | Sequence 1 | Berries | 6509 | 6512 | 0.10 | Ingestive | Oral-Manual | Posterior |
| ‘watermelon a beach sequence good_1’ | Cenoura | AF | Sequence 1 | Berries | 6512 | 6573 | 2.03 | Masticate |  |  |
| ‘watermelon a beach sequence good_1’ | Cenoura | AF | Sequence 1 | Berries | 6573 | 6582 | 0.30 | Ingestive | Oral-Manual | Anterior |
| ‘watermelon a beach sequence good_1’ | Cenoura | AF | Sequence 1 | Berries | 6582 | 6606 | 0.80 | Masticate |  |  |
| ‘watermelon a beach sequence good_1’ | Cenoura | AF | Sequence 1 | Berries | 6606 | 6641 | 1.17 | Ingestive | Oral-Manual | Posterior |
| ‘watermelon a beach sequence good_1’ | Cenoura | AF | Sequence 1 | Berries | 6677 | 6687 | 0.33 | Ingestive | Oral-Manual | Anterior |
| ‘watermelon a beach sequence good_1’ | Cenoura | AF | Sequence 1 | Berries | 6687 | 6738 | 1.70 | Masticate |  |  |
| ‘watermelon a beach sequence good_1’ | Cenoura | AF | Sequence 1 | Berries | 6738 | 6742 | 0.13 |  |  |  |
| ‘watermelon a beach sequence good_1’ | Cenoura | AF | Sequence 1 | Berries | 6766 | 6775 | 0.30 |  |  |  |
| ‘watermelon a beach sequence good_1’ | Cenoura | AF | Sequence 1 | Berries | 6838 | 6883 | 1.50 | Ingestive | Oral-Manual | Posterior |
| ‘watermelon a beach sequence good_1’ | Cenoura | AF | Sequence 1 | Berries | 6883 | 7016 | 4.43 | Masticate |  |  |
| ‘watermelon a beach sequence good_1’ | Cenoura | AF | Sequence 1 | Berries | 7016 | 7019 | 0.10 | Ingestive | Oral-Manual | Posterior |
| ‘watermelon a beach sequence good_1’ | Cenoura | AF | Sequence 1 | Berries | 7019 | 7124 | 3.50 | Masticate |  |  |
| ‘watermelon a beach sequence good_1’ | Cenoura | AF | Sequence 1 | Berries | 7124 | 7128 | 0.13 | Ingestive | Oral-Manual | Posterior |
| ‘watermelon a beach sequence good_1’ | Cenoura | AF | Sequence 1 | Berries | 7128 | 7201 | 2.43 | Masticate |  |  |
| ‘watermelon a beach sequence good_1’ | Cenoura | AF | Sequence 1 | Berries | 7201 | 7223 | 0.73 | Ingestive | Oral-Manual | Posterior |
| ‘watermelon a beach sequence good_1’ | Cenoura | AF | Sequence 1 | Berries | 7223 | 7249 | 0.87 | Masticate |  |  |
| ‘watermelon a beach sequence good_1’ | Cenoura | AF | Sequence 1 | Berries | 7249 | 7307 | 1.93 | Ingestive | Oral-Manual | Posterior |
| ‘watermelon a beach sequence good_1’ | Cenoura | AF | Sequence 1 | Berries | 7307 | 7382 | 2.50 | Masticate |  |  |
| ‘watermelon a beach sequence good_1’ | Cenoura | AF | Sequence 1 | Berries | 7382 | 7445 | 2.10 | Ingestive | Oral-Manual | Posterior |
| ‘watermelon a beach sequence good_1’ | Cenoura | AF | Sequence 1 | Berries | 7445 | 7585 | 4.67 | Masticate |  |  |
| ‘watermelon a beach sequence good_1’ | Cenoura | AF | Sequence 1 | Berries | 7923 | 7953 | 1.00 | Ingestive | Oral-Manual | Anterior |
| ‘watermelon a beach sequence good_1’ | Cenoura | AF | Sequence 1 | Berries | 7980 | 7992 | 0.40 | Ingestive | Oral-Manual | Posterior |
| ‘watermelon a beach sequence good_1’ | Cenoura | AF | Sequence 1 | Berries | 8068 | 8129 | 2.03 | Ingestive | Oral-Manual | Anterior |
| ‘watermelon a beach sequence good_1’ | Cenoura | AF | Sequence 1 | Berries | 8321 | 8435 | 3.80 | Ingestive | Oral-Manual | Anterior |
| ‘watermelon a beach sequence good_1’ | Cenoura | AF | Sequence 1 | Berries | 8477 | 8516 | 1.30 | Ingestive | Oral-Manual | Anterior |
| ‘watermelon a beach sequence good_1’ | Cenoura | AF | Sequence 1 | Berries | 8516 | 8540 | 0.80 | Masticate |  |  |
| ‘watermelon a beach sequence good_1’ | Cenoura | AF | Sequence 1 | Berries | 8540 | 8550 | 0.33 | Ingestive | Oral-Manual | Anterior |
| ‘watermelon a beach sequence good_1’ | Cenoura | AF | Sequence 1 | Berries | 8550 | 8614 | 2.13 | Masticate |  |  |
| ‘watermelon a beach sequence good_1’ | Cenoura | AF | Sequence 1 | Berries | 8614 | 8636 | 0.73 | Ingestive | Oral-Manual | Anterior |
| ‘watermelon a beach sequence good_1’ | Cenoura | AF | Sequence 1 | Berries | 8636 | 8723 | 2.90 | Masticate |  |  |
| ‘watermelon a beach sequence good_1’ | Cenoura | AF | Sequence 1 | Berries | 8723 | 8725 | 0.07 | Ingestive | Oral-Manual | Anterior |
| ‘watermelon a beach sequence good_1’ | Cenoura | AF | Sequence 1 | Berries | 8725 | 8890 | 5.50 | Masticate |  |  |
| ‘watermelon a beach sequence good_1’ | Cenoura | AF | Sequence 1 | Berries | 8890 | 8891 | 0.03 | Ingestive | Oral-Manual | Anterior |
| ‘watermelon a beach sequence good_1’ | Cenoura | AF | Sequence 1 | Berries | 8891 | 8994 | 3.43 | Masticate |  |  |
| ‘watermelon a beach sequence good_1’ | Cenoura | AF | Sequence 1 | Berries | 8994 | 8996 | 0.07 | Ingestive | Oral-Manual | Anterior |
| ‘watermelon a beach sequence good_1’ | Cenoura | AF | Sequence 1 | Berries | 8996 | 9100 | 3.47 | Masticate |  |  |
| ‘watermelon a beach sequence good_1’ | Cenoura | AF | Sequence 1 | Berries | 9818 | 9831 | 0.43 | Ingestive | Oral-Manual | Posterior |
| ‘watermelon a beach sequence good_2’ | Cenoura | AF | Sequence 1 | Berries | 1 | 38 | 1.23 | Ingestive | Oral-Manual | Anterior |
| ‘watermelon a beach sequence good_2’ | Cenoura | AF | Sequence 1 | Berries | 63 | 111 | 1.60 | Ingestive | Oral-Manual | Anterior |
| ‘watermelon a beach sequence good_2’ | Cenoura | AF | Sequence 1 | Berries | 134 | 173 | 1.30 | Ingestive | Oral-Manual | Anterior |
| ‘watermelon a beach sequence good_2’ | Cenoura | AF | Sequence 1 | Berries | 194 | 208 | 0.47 | Ingestive | Oral-Manual | Anterior |
| ‘watermelon a beach sequence good_2’ | Cenoura | AF | Sequence 1 | Berries | 263 | 304 | 1.37 | Ingestive | Oral-Manual | Anterior |
| ‘watermelon a beach sequence good_2’ | Cenoura | AF | Sequence 1 | Berries | 413 | 543 | 4.33 | Ingestive | Oral-Manual | Anterior |
| ‘watermelon a beach sequence good_2’ | Cenoura | AF | Sequence 1 | Berries | 543 | 638 | 3.17 | Ingestive | Oral-Manual | Posterior |
| ‘watermelon a beach sequence good_2’ | Cenoura | AF | Sequence 1 | Berries | 687 | 703 | 0.53 | Ingestive | Oral-Manual | Anterior |
| ‘watermelon a beach sequence good_2’ | Cenoura | AF | Sequence 1 | Berries | 758 | 888 | 4.33 | Ingestive | Oral-Manual | Anterior |
| ‘watermelon a beach sequence good_2’ | Cenoura | AF | Sequence 1 | Berries | 918 | 939 | 0.70 | Ingestive | Oral-Manual | Anterior |
| ‘watermelon a beach sequence good_2’ | Cenoura | AF | Sequence 1 | Berries | 965 | 974 | 0.30 | Ingestive | Oral-Manual | Posterior |
| ‘watermelon a beach sequence good_2’ | Cenoura | AF | Sequence 1 | Berries | 974 | 1132 | 5.27 | Masticate |  |  |
| ‘watermelon a beach sequence good_3’ | Cenoura | AF | Sequence 1 | Berries | 1 | 32 | 1.03 | Ingestive | Oral-Manual | Anterior |
| ‘watermelon a beach sequence good_3’ | Cenoura | AF | Sequence 1 | Berries | 54 | 85 | 1.03 | Ingestive | Oral-Manual | Posterior |
| ‘watermelon a beach sequence good_3’ | Cenoura | AF | Sequence 1 | Berries | 110 | 125 | 0.50 | Ingestive | Oral-Manual | Anterior |
| ‘watermelon a beach sequence good_3’ | Cenoura | AF | Sequence 1 | Berries | 182 | 199 | 0.57 | Ingestive | Oral-Manual | Anterior |
| ‘watermelon a beach sequence good_3’ | Cenoura | AF | Sequence 1 | Berries | 227 | 237 | 0.33 | Ingestive | Oral-Manual | Posterior |
| ‘watermelon a beach sequence good_3’ | Cenoura | AF | Sequence 1 | Berries | 324 | 399 | 2.50 | Ingestive | Oral-Manual | Anterior |
| ‘watermelon a beach sequence good_3’ | Cenoura | AF | Sequence 1 | Berries | 417 | 437 | 0.67 | Ingestive | Oral-Manual | Anterior |
| ‘watermelon a beach sequence good_3’ | Cenoura | AF | Sequence 1 | Berries | 460 | 562 | 3.40 | Ingestive | Oral-Manual | Posterior |
| ‘watermelon a beach sequence good_3’ | Cenoura | AF | Sequence 1 | Berries | 616 | 665 | 1.63 | Ingestive | Oral-Manual | Anterior |
| ‘watermelon a beach sequence good_3’ | Cenoura | AF | Sequence 1 | Berries | 694 | 715 | 0.70 | Ingestive | Oral-Manual | Anterior |
| ‘watermelon a beach sequence good_3’ | Cenoura | AF | Sequence 1 | Berries | 739 | 753 | 0.47 | Ingestive | Oral-Manual | Anterior |
| ‘watermelon a beach sequence good_3’ | Cenoura | AF | Sequence 1 | Berries | 753 | 846 | 3.10 | Ingestive | Oral-Manual | Posterior |

**SOM Table S3**

Links to all behavior videos. Video names correspond with the videos in SOM Table S2.

| **Video name** | **Link** |
| --- | --- |
| ‘B5IL4F~K’ | https://drive.google.com/file/d/12X5pilZT43A1zx9WsylbhL762H8H_nCM/view?usp=sharing |
| ‘big tuber’ | https://drive.google.com/file/d/12YfPb7ymn6xwZF3mb7QXbsHdb5PO3VUV/view?usp=sharing |
| ‘biig tuber’ | https://drive.google.com/file/d/12dGbXbVWydLsIiISyUjNcELm7rO50n4v/view?usp=sharing |
| ‘brom flowers ok_1’ | https://drive.google.com/file/d/1uP9DhFeNiLzZXejh3CzrNGyzmwXnM0XA/view?usp=sharing |
| ‘brom flowers ok_2’ | https://drive.google.com/file/d/1kHf_CHpl_kl8E6Mh1_MAhLk7rrPErg8C/view?usp=sharing |
| ‘brom flowers ok_3’ | https://drive.google.com/file/d/1XWPUmXdeJx-QaP9R_QhzsDW3aQReH0-G/view?usp=sharing |
| ‘bromedliad_extra good_1’ | https://drive.google.com/file/d/1aMxiCE8mGeEsvqvHqIm4wYCFrENRgjes/view?usp=sharing |
| ‘bromedliad_extra good_2’ | https://drive.google.com/file/d/1-082a6Shg38gZYk0nDaNyjFeLQDgGVNV/view?usp=sharing |
| ‘bromedliad_extra good_3’ | https://drive.google.com/file/d/1c15GW-XfFB6lbi4Kgq5j6gvC782c_hF8/view?usp=sharing |
| ‘Coco_jatoba pod_1_1’ | https://drive.google.com/file/d/1mGs7vkV6nettn2mPaXS0lstZOCuuOWxg/view?usp=sharing |
| ‘Coco_jatoba pod_1_2’ | https://drive.google.com/file/d/1HgKbWM49XiUbQFljqiLIZizsDFSJac2D/view?usp=sharing |
| ‘complete tuber seq_1’ | https://drive.google.com/file/d/18FUNtk2N_2N8SFqFC07oH8a0O0TvwcK7/view?usp=sharing |
| ‘complete tuber seq_2’ | https://drive.google.com/file/d/1irzzI2DpM0x6Aw7x_ZIeLA-VBBmsZfT0/view?usp=sharing |
| ‘eating summit’ | https://drive.google.com/file/d/132sx7e7LsYLHGeyGMSfuwcaozBrPoDa6/view?usp=sharing |
| ‘family got the tuber’ | https://drive.google.com/file/d/135APAmQ_kF7vwf670j-5GpFS9ca9Nbug/view?usp=sharing |
| ‘good m de boi’ | https://drive.google.com/file/d/132xIJcMWOgwTHLL-HKfY0yDhHaYmBfF3/view?usp=sharing |
| ‘‘i’m yellow podding’ 2’ | https://drive.google.com/file/d/132xIJcMWOgwTHLL-HKfY0yDhHaYmBfF3/view?usp=sharing |
| ‘i’m yellow podding’ | https://drive.google.com/file/d/13F-9r_QO0BGbGzZ3RPcQc-iT8UkDAPra/view?usp=sharing |
| ‘jatoba complete tucum seq 4’ | https://drive.google.com/file/d/13Mc0zgNekxribaum9rXahOgeCGXn2C97/view?usp=sharing |
| ‘jatoba complete tucum seq 5’ | https://drive.google.com/file/d/13SLUqbuJbL5ij_rF15srbrkFPdA4Np8e/view?usp=sharing |
| ‘jatoba cracks and rejects’ | https://drive.google.com/file/d/13at3qVniqiOXkK7eYxJE9VS4pC3coFrc/view?usp=sharing |
| ‘jatoba nut scratching’ | https://drive.google.com/file/d/13aK8e8eOJNpLDRMeTh1uMz8DDBZ4XCtx/view?usp=sharing |
| ‘jatoba on anvil’ | https://drive.google.com/file/d/13o6F6Bgvi2pk6l3d3OnFzPhZVoPVUj8q/view?usp=sharing |
| ‘jatoba Piaçava seq 1_1’ | https://drive.google.com/file/d/18j_yktmYKGltz6rvLyjfB_KDDHjW-RfE/view?usp=sharing |
| ‘jatoba Piaçava seq 1_2’ | https://drive.google.com/file/d/1hyZzCVIkSMSXzMrrYPac_BBZ0uLKbRU3/view?usp=sharing |
| ‘juvie fruit nice_1’ | https://drive.google.com/file/d/1NuhJAhMSD8aw5vYnP7QeEo6T9a8P2iX8/view?usp=sharing |
| ‘juvie fruit nice_2’ | https://drive.google.com/file/d/1QFAuiHhQQSCCiSzdDQ67vq6BJ3T09act/view?usp=sharing |
| ‘m de boi more’ | https://drive.google.com/file/d/13wLOHJkfm8ph2WtX5zin-p_dX_uQ7pNT/view?usp=sharing |
| ‘m de boi nahce_1’ | https://drive.google.com/file/d/128XkVr2neZAjgOG5CxGmVnqgrY3_TgyI/view?usp=sharing |
| ‘m de boi nahce_2’ | https://drive.google.com/file/d/1LfZo6XeI18VWgrQlb0WwmSZE-OG2fxJR/view?usp=sharing |
| ‘m de boi nahce_3’ | https://drive.google.com/file/d/1zvDZgtNfI9-yfT-sqvyZbNkxxWDE-1vF/view?usp=sharing |
| ‘m de boi nahce_4’ | https://drive.google.com/file/d/1OlREkZEGbwplfdS3VW_CkEnH3gSu50oH/view?usp=sharing |
| ‘m de boi nice seqs’ | https://drive.google.com/file/d/14AHKfQctdx3gjGrGEaY4Zqk1k3i-TIEa/view?usp=sharing |
| ‘m de boi nice’ | https://drive.google.com/file/d/14-2-IHYWbiu9h9HlweRaIc7Sfdtucj2E/view?usp=sharing |
| ‘m de boi seq’ | https://drive.google.com/file/d/14Fi70nfIsqJuj3s7aGcCjsVEp1HRlxvs/view?usp=sharing |
| ‘m de boi sum momo’ | https://drive.google.com/file/d/14PSlMzOTpjMZ3CrqMiA4Ol1BKF1LszTe/view?usp=sharing |
| ‘m de boi’ | https://drive.google.com/file/d/14P474JjYtT5Ec1gJbkKiy8eepr9yl38J/view?usp=sharing |
| ‘marm de boi_1’ | https://drive.google.com/file/d/1b7Lzv6dRLkHfYJOa6VQoLBl6qjeN2pXH/view?usp=sharing |
| ‘marm de boi_2’ | https://drive.google.com/file/d/10skzaS6LCQfJTJslflL8JgaVCXOLuTbB/view?usp=sharing |
| ‘monk marmalatta’ | https://drive.google.com/file/d/14dcj0-I3KWe4naXHLhxPQxyOLiM2lZM9/view?usp=sharing |
| ‘monkey twigging’ | https://drive.google.com/file/d/1ct7iMdxd_tG7M0nnpyMrEW9nLuW-QM4J/view?usp=sharing |
| ‘nice juvie feeding’ | https://drive.google.com/file/d/14jW00DWJP1g1_aSitmng4uwcDmEZFzUa/view?usp=sharing |
| ‘nice juvie marmalatta_1’ | https://drive.google.com/file/d/1EJUfad75cSD7jBH-3RJXG2-sP7IAn68T/view?usp=sharing |
| ‘nice juvie marmalatta_2’ | https://drive.google.com/file/d/1VfRLqu7UxWTWodU74QRCw-PIVqV3QAbD/view?usp=sharing |
| ‘nice monk green fruit_1’ | https://drive.google.com/file/d/1lck0_v0uZs5rA4t8hEiDdCHkVXQnJc23/view?usp=sharing |
| ‘nice monk green fruit_2’ | https://drive.google.com/file/d/1acFvFKnUu4jkyrohkj4jz3xJXtLKr-aI/view?usp=sharing |
| ‘nice monk green fruit_3’ | https://drive.google.com/file/d/18Oun7Oci72TwMeSwAT4dNQHGeM9NjerF/view?usp=sharing |
| ‘nice pc biting_1’ | https://drive.google.com/file/d/1Ka4XKVUiAo2a9FC-hFkPkFyHtmwsxItO/view?usp=sharing |
| ‘nice pc biting_2’ | https://drive.google.com/file/d/1eXk1UE1OIBPlb5SNYeYYvY13s4e8MwU9/view?usp=sharing |
| ‘nice pc biting_3’ | https://drive.google.com/file/d/1wQdrlgQn5B4pnGWKppKVOkWCmVAh8dUC/view?usp=sharing |
| ‘nice pc biting_4’ | https://drive.google.com/file/d/1s0H91tlD21juogIbvdOloY5YF5Y4C0u7/view?usp=sharing |
| ‘nice tuberosity_1’ | https://drive.google.com/file/d/13aSl6vAH4DJdMzAkLt-ONo9V6A8H8Pnn/view?usp=sharing |
| ‘nice tuberosity_2’ | https://drive.google.com/file/d/1A_afSVjfn99yjATxHDelUAKuBtNBjLLI/view?usp=sharing |
| ‘nice tuberosity_3’ | https://drive.google.com/file/d/1kZhGuGfK2WNnWkFmmeWhcxBLrDODJbX2/view?usp=sharing |
| ‘nice tuberosity_4’ | https://drive.google.com/file/d/1ZbOWZ0UW6SEaK2i7OJdhCf-moyFKvP5x/view?usp=sharing |
| ‘nice tuberosity_5’ | https://drive.google.com/file/d/18o8vPnu2OmntrwRCdOKzuu4ENbEVT8SP/view?usp=sharing |
| ‘palm 1_1’ | https://drive.google.com/file/d/1GwMlPEsrLV-qKZWDQzo3HqWH3ESe0Dn8/view?usp=sharing |
| ‘palm 1_2’ | https://drive.google.com/file/d/1RgNbhI2x08HQLDoIpauag9PlPLVFGgRE/view?usp=sharing |
| ‘palm 1_3’ | https://drive.google.com/file/d/1Ka-ieZ-nuy57SOv7AunboT8MTSv8gGbi/view?usp=sharing |
| ‘palm biting for bugs_1’ | https://drive.google.com/file/d/153uIoUSVrCFJ10Sy6mMQEbm5zxFbxB_r/view?usp=sharing |
| ‘palm biting for bugs_2’ | https://drive.google.com/file/d/1gNKwwY--33xchMcP67dkryaGdJGItWOW/view?usp=sharing |
| ‘piasava nutting’ | https://drive.google.com/file/d/15BOlD4ioXa8sgIiyOAg66jearQdYZ7vO/view?usp=sharing |
| ‘random monkeys’ | https://drive.google.com/file/d/15EZz-uzR9bo8A23gaRSxIK2EknkA8pVb/view?usp=sharing |
| ‘SC good 3_1’ | https://drive.google.com/file/d/1xQgIU-SHTlkA_-xK2-BS4dmZJvujpkPJ/view?usp=sharing |
| ‘SC good 3_2’ | https://drive.google.com/file/d/1s-9n_w0tGmIq66Mf2v8mh_fDo-hqVJoD/view?usp=sharing |
| ‘SC good 3_3’ | https://drive.google.com/file/d/1szn-1CAVeKujEqhKhXPXU7b2FxfqYaaD/view?usp=sharing |
| ‘SC good 4_1’ | https://drive.google.com/file/d/1O_geiv-bJDv-s6O6lne63NI-5vef95hV/view?usp=sharing |
| ‘SC good 4_2’ | https://drive.google.com/file/d/1FxlHtXnSl8hwmGlmMFpOVOcFJy0H_McT/view?usp=sharing |
| ‘SC good 5_1’ | https://drive.google.com/file/d/1hcUrPMIgx0jcW-tdSYsVOgLDVDeImRnl/view?usp=sharing |
| ‘SC good 5_2’ | https://drive.google.com/file/d/1wfCycKwFAX3lAzKqMobF8d-iNPF3S_N0/view?usp=sharing |
| ‘SC good chews 6’ | https://drive.google.com/file/d/15g-xJRfkM3WdnzYSmwiG0NfhnRCmRbP2/view?usp=sharing |
| ‘SC good_1’ | https://drive.google.com/file/d/1yZpeOQuSYH_FZeMeFV4UUwRilNnJoRvi/view?usp=sharing |
| ‘SC good_2’ | https://drive.google.com/file/d/1qd7xq1s6nLJiMEyIMlmZfnoX3cbiSwlg/view?usp=sharing |
| ‘SC good1_1’ | https://drive.google.com/file/d/1xyCPVN__qUsaVgbz8eV7pcZGFhK8mzP9/view?usp=sharing |
| ‘SC good1_2’ | https://drive.google.com/file/d/1dzwAim0BfDTlk7uPvsjrXDxOOj4yu4ql/view?usp=sharing |
| ‘sloping anvil_3’ | https://drive.google.com/file/d/1FAbcQ851NAfW7HGcI29m-uvRCvhl5KRL/view?usp=sharing |
| ‘sloping anvil_4’ | https://drive.google.com/file/d/1exyEacQhv_LJXwG3vfT6LycmJ2rIbsWc/view?usp=sharing |
| ‘sloping anvil_7’ | https://drive.google.com/file/d/1X08hKBjwjYAKb9mY6UobtNjoTZ-L1Bpl/view?usp=sharing |
| ‘still more m de boi_1’ | https://drive.google.com/file/d/1MTYNZiP1kZRYWcO2XfJ_OzEQxUunUOfl/view?usp=sharing |
| ‘still more m de boi_2’ | https://drive.google.com/file/d/1rtdKwoPrHgUqW4I7JJP5vizErLkGQMzF/view?usp=sharing |
| ‘Tiese_tuber_spiderhead_2’ | https://drive.google.com/file/d/14-ZIIH8GLdmx0eY14W6rIy8Vwy7R61t6/view?usp=sharing |
| ‘Tiese_tuber_spiderhead_3’ | https://drive.google.com/file/d/1UUFhkCGIN6YryRvzrhiE5AdI6HTmWxF0/view?usp=sharing |
| ‘tiny bananas very good’ | https://drive.google.com/file/d/162w35VqtyWh6jtMb1HupCMXcnV27BvND/view?usp=sharing |
| ‘watermelon a beach sequence good_1’ | https://drive.google.com/file/d/14mOmXIvBnQXOhl222nEgwHugbJAYdGcc/view?usp=sharing |
| ‘watermelon a beach sequence good_2’ | https://drive.google.com/file/d/1ZN8AM-aTTRM3pryGfnpelMU2K8enPsGk/view?usp=sharing |
| ‘watermelon a beach sequence good_3’ | https://drive.google.com/file/d/16YFf24LLL3TdtViuKda1movO_KAWrVov/view?usp=sharing |

**SOM Table S4 (formerly s3)**

Results of a Dunn test for multiple comparisons with a Bonferroni correction testing for pairwise differences in toughness values between food items. Significant values are bolded. Pairwise comparisons suggest that almost all foods overlap in their range of toughness values, but the exocarp of the accessory hypocarp (also known as the pseudo-fruit or apple) of ‘fruta de caju’ (*Anacardium* sp.) was significantly tougher than ‘piaçava’ kernel (*p* = 0.03) and mesocarp (*p* = 0.01). Cane was significantly tougher than ‘fruta d'anta’ exocarp (*p* = 0.02) and ‘piaçava’ kernel (*p* = 0.03) and mesocarp (*p* = 0.02).

| Test number | Comparison | | Z | Unadjusted *p* | | Adjusted *p* | | |
| --- | --- | --- | --- | --- | --- | --- | --- | --- |
| 1 | Bananinha- Berry | -0.22488 | | | 8.22E-01 | | 1 |  |
| 2 | Bananinha- Bromeliad leaf | 1.293041 | | | 1.96E-01 | | 1 |  |
| 3 | Berry - Bromeliad leaf | 1.385663 | | | 1.66E-01 | | 1 |  |
| 4 | Bananinha- Cane | -1.41123 | | | 1.58E-01 | | 1 |  |
| 5 | Berry - Cane | -0.92393 | | | 3.56E-01 | | 1 |  |
| 6 | Bromeliad leaf - Cane | -2.73819 | | | 6.18E-03 | | 1 |  |
| 7 | Bananinha- Fruta podoin-seed | 0.590837 | | | 5.55E-01 | | 1 |  |
| 8 | Berry - Fruta podoin-seed | 0.753337 | | | 4.51E-01 | | 1 |  |
| 9 | Bromeliad leaf - Fruta podoin-seed | -0.76458 | | | 4.45E-01 | | 1 |  |
| 10 | Cane - Fruta podoin-seed | 2.158586 | | | 3.09E-02 | | 1 |  |
| 11 | Bananinha- Fruta d'anta-endocarp | 1.883343 | | | 5.97E-02 | | 1 |  |
| 12 | Berry - Fruta d'anta-endocarp | 1.924532 | | | 5.43E-02 | | 1 |  |
| 13 | Bromeliad leaf - Fruta d'anta-endocarp | 0.538869 | | | 5.90E-01 | | 1 |  |
| 14 | Cane - Fruta d'anta-endocarp | 3.443732 | | | 5.74E-04 | | 0.12 |  |
| 15 | Fruta podoin-seed - Fruta d'anta-endocarp | 1.354882 | | | 1.75E-01 | | 1 |  |
| 16 | Bananinha- Fruta d'anta-exocarp | 1.83624 | | | 6.63E-02 | | 1 |  |
| 17 | Berry - Fruta d'anta-exocarp | 1.84165 | | | 6.55E-02 | | 1 |  |
| 18 | Bromeliad leaf - Fruta d'anta-exocarp | 0.144566 | | | 8.85E-01 | | 1 |  |
| 19 | Cane - Fruta d'anta-exocarp | 4.418726 | | | 9.93E-06 | | **<0.01** |  |
| 20 | Fruta podoin-seed - Fruta d'anta-exocarp | 1.154001 | | | 2.48E-01 | | 1 |  |
| 21 | Fruta d'anta-endocarp - Fruta d'anta-exocarp | -0.51541 | | | 6.06E-01 | | 1 |  |
| 22 | Bananinha- Fruta d'anta-mesocarp | 1.548839 | | | 1.21E-01 | | 1 |  |
| 23 | Berry - Fruta d'anta-mesocarp | 1.597298 | | | 1.10E-01 | | 1 |  |
| 24 | Bromeliad leaf - Fruta d'anta-mesocarp | -0.05889 | | | 9.53E-01 | | 1 |  |
| 25 | Cane - Fruta d'anta-mesocarp | 3.836357 | | | 1.25E-04 | | **0.03** |  |
| 26 | Fruta podoin-seed - Fruta d'anta-mesocarp | 0.888263 | | | 3.74E-01 | | 1 |  |
| 27 | Fruta d'anta-endocarp - Fruta d'anta-mesocarp | -0.70296 | | | 4.82E-01 | | 1 |  |
| 28 | Fruta d'anta-exocarp - Fruta d'anta-mesocarp | -0.2763 | | | 7.82E-01 | | 1 |  |
| 29 | Bananinha- Fruta de Caju-endocarp | -0.54055 | | | 5.89E-01 | | 1 |  |
| 30 | Berry - Fruta de Caju-endocarp | -0.25861 | | | 7.96E-01 | | 1 |  |
| 31 | Bromeliad leaf - Fruta de Caju-endocarp | -1.77653 | | | 7.56E-02 | | 1 |  |
| 32 | Cane - Fruta de Caju-endocarp | 0.727479 | | | 4.67E-01 | | 1 |  |
| 33 | Fruta podoin-seed - Fruta de Caju-endocarp | -1.13139 | | | 2.58E-01 | | 1 |  |
| 34 | Fruta d'anta-endocarp - Fruta de Caju-endocarp | -2.36683 | | | 1.79E-02 | | 1 |  |
| 35 | Fruta d'anta-exocarp - Fruta de Caju-endocarp | -2.46042 | | | 1.39E-02 | | 1 |  |
| 36 | Fruta d'anta-mesocarp - Fruta de Caju-endocarp | -2.15319 | | | 3.13E-02 | | 1 |  |
| 37 | Bananinha- Fruta de Caju-exocarp | -1.84794 | | | 6.46E-02 | | 1 |  |
| 38 | Berry - Fruta de Caju-exocarp | -1.42797 | | | 1.53E-01 | | 1 |  |
| 39 | Bromeliad leaf - Fruta de Caju-exocarp | -2.94589 | | | 3.22E-03 | | 0.68 |  |
| 40 | Cane - Fruta de Caju-exocarp | -0.92624 | | | 3.54E-01 | | 1 |  |
| 41 | Fruta podoin-seed - Fruta de Caju-exocarp | -2.43877 | | | 1.47E-02 | | 1 |  |
| 42 | Fruta d'anta-endocarp - Fruta de Caju-exocarp | -3.53619 | | | 4.06E-04 | | 0.09 |  |
| 43 | Fruta d'anta-exocarp - Fruta de Caju-exocarp | -3.97005 | | | 7.19E-05 | | **0.02** |  |
| 44 | Fruta d'anta-mesocarp - Fruta de Caju-exocarp | -3.61489 | | | 3.00E-04 | | 0.06 |  |
| 45 | Fruta de Caju-endocarp - Fruta de Caju-exocarp | -1.30738 | | | 1.91E-01 | | 1 |  |
| 46 | Bananinha- Fruta de Caju-mesocarp | -0.62855 | | | 5.30E-01 | | 1 |  |
| 47 | Berry - Fruta de Caju-mesocarp | -0.33732 | | | 7.36E-01 | | 1 |  |
| 48 | Bromeliad leaf - Fruta de Caju-mesocarp | -1.85523 | | | 6.36E-02 | | 1 |  |
| 49 | Cane - Fruta de Caju-mesocarp | 0.616171 | | | 5.38E-01 | | 1 |  |
| 50 | Fruta podoin-seed - Fruta de Caju-mesocarp | -1.21939 | | | 2.23E-01 | | 1 |  |
| 51 | Fruta d'anta-endocarp - Fruta de Caju-mesocarp | -2.44553 | | | 1.45E-02 | | 1 |  |
| 52 | Fruta d'anta-exocarp - Fruta de Caju-mesocarp | -2.56203 | | | 1.04E-02 | | 1 |  |
| 53 | Fruta d'anta-mesocarp - Fruta de Caju-mesocarp | -2.25158 | | | 2.43E-02 | | 1 |  |
| 54 | Fruta de Caju-endocarp - Fruta de Caju-mesocarp | -0.088 | | | 9.30E-01 | | 1 |  |
| 55 | Fruta de Caju-exocarp - Fruta de Caju-mesocarp | 1.219386 | | | 2.23E-01 | | 1 |  |
| 56 | Bananinha- Fruta podoin-mesocarp | -0.39918 | | | 6.90E-01 | | 1 |  |
| 57 | Berry - Fruta podoin-mesocarp | -0.09428 | | | 9.25E-01 | | 1 |  |
| 58 | Bromeliad leaf - Fruta podoin-mesocarp | -1.79137 | | | 7.32E-02 | | 1 |  |
| 59 | Cane - Fruta podoin-mesocarp | 1.257361 | | | 2.09E-01 | | 1 |  |
| 60 | Fruta podoin-seed - Fruta podoin-mesocarp | -1.08142 | | | 2.80E-01 | | 1 |  |
| 61 | Fruta d'anta-endocarp - Fruta podoin-mesocarp | -2.45134 | | | 1.42E-02 | | 1 |  |
| 62 | Fruta d'anta-exocarp - Fruta podoin-mesocarp | -2.73782 | | | 6.18E-03 | | 1 |  |
| 63 | Fruta d'anta-mesocarp - Fruta podoin-mesocarp | -2.33411 | | | 1.96E-02 | | 1 |  |
| 64 | Fruta de Caju-endocarp - Fruta podoin-mesocarp | 0.224994 | | | 8.22E-01 | | 1 |  |
| 65 | Fruta de Caju-exocarp - Fruta podoin-mesocarp | 1.73463 | | | 8.28E-02 | | 1 |  |
| 66 | Fruta de Caju-mesocarp - Fruta podoin-mesocarp | 0.326604 | | | 7.44E-01 | | 1 |  |
| 67 | Bananinha- Insects | 0.817114 | | | 4.14E-01 | | 1 |  |
| 68 | Berry - Insects | 0.955726 | | | 3.39E-01 | | 1 |  |
| 69 | Bromeliad leaf - Insects | -0.56219 | | | 5.74E-01 | | 1 |  |
| 70 | Cane - Insects | 2.444807 | | | 1.45E-02 | | 1 |  |
| 71 | Fruta podoin-seed - Insects | 0.226278 | | | 8.21E-01 | | 1 |  |
| 72 | Fruta d'anta-endocarp - Insects | -1.15249 | | | 2.49E-01 | | 1 |  |
| 73 | Fruta d'anta-exocarp - Insects | -0.89272 | | | 3.72E-01 | | 1 |  |
| 74 | Fruta d'anta-mesocarp - Insects | -0.63528 | | | 5.25E-01 | | 1 |  |
| 75 | Fruta de Caju-endocarp - Insects | 1.357667 | | | 1.75E-01 | | 1 |  |
| 76 | Fruta de Caju-exocarp - Insects | 2.66505 | | | 7.70E-03 | | 1 |  |
| 77 | Fruta de Caju-mesocarp - Insects | 1.445664 | | | 1.48E-01 | | 1 |  |
| 78 | Fruta podoin-mesocarp - Insects | 1.342705 | | | 1.79E-01 | | 1 |  |
| 79 | Bananinha- Piaçava-exocarp | -0.7929 | | | 4.28E-01 | | 1 |  |
| 80 | Berry - Piaçava-exocarp | -0.46223 | | | 6.44E-01 | | 1 |  |
| 81 | Bromeliad leaf - Piaçava-exocarp | -2.06226 | | | 3.92E-02 | | 1 |  |
| 82 | Cane - Piaçava-exocarp | 0.528898 | | | 5.97E-01 | | 1 |  |
| 83 | Fruta podoin-seed - Piaçava-exocarp | -1.42453 | | | 1.54E-01 | | 1 |  |
| 84 | Fruta d'anta-endocarp - Piaçava-exocarp | -2.68449 | | | 7.26E-03 | | 1 |  |
| 85 | Fruta d'anta-exocarp - Piaçava-exocarp | -2.94967 | | | 3.18E-03 | | 0.67 |  |
| 86 | Fruta d'anta-mesocarp - Piaçava-exocarp | -2.58892 | | | 9.63E-03 | | 1 |  |
| 87 | Fruta de Caju-endocarp - Piaçava-exocarp | -0.21502 | | | 8.30E-01 | | 1 |  |
| 88 | Fruta de Caju-exocarp - Piaçava-exocarp | 1.182628 | | | 2.37E-01 | | 1 |  |
| 89 | Fruta de Caju-mesocarp - Piaçava-exocarp | -0.12095 | | | 9.04E-01 | | 1 |  |
| 90 | Fruta podoin-mesocarp - Piaçava-exocarp | -0.50089 | | | 6.16E-01 | | 1 |  |
| 91 | Insects - Piaçava-exocarp | -1.66643 | | | 9.56E-02 | | 1 |  |
| 92 | Bananinha- Piaçava-kernel | 1.563916 | | | 1.18E-01 | | 1 |  |
| 93 | Berry - Piaçava-kernel | 1.602038 | | | 1.09E-01 | | 1 |  |
| 94 | Bromeliad leaf - Piaçava-kernel | -0.12619 | | | 9.00E-01 | | 1 |  |
| 95 | Cane - Piaçava-kernel | 4.184548 | | | 2.86E-05 | | **0.01** |  |
| 96 | Fruta podoin-seed - Piaçava-kernel | 0.864828 | | | 3.87E-01 | | 1 |  |
| 97 | Fruta d'anta-endocarp - Piaçava-kernel | -0.79828 | | | 4.25E-01 | | 1 |  |
| 98 | Fruta d'anta-exocarp - Piaçava-kernel | -0.39402 | | | 6.94E-01 | | 1 |  |
| 99 | Fruta d'anta-mesocarp - Piaçava-kernel | -0.08865 | | | 9.29E-01 | | 1 |  |
| 100 | Fruta de Caju-endocarp - Piaçava-kernel | 2.203506 | | | 2.76E-02 | | 1 |  |
| 101 | Fruta de Caju-exocarp - Piaçava-kernel | 3.750423 | | | 1.77E-04 | | **0.04** |  |
| 102 | Fruta de Caju-mesocarp - Piaçava-kernel | 2.307626 | | | 2.10E-02 | | 1 |  |
| 103 | Fruta podoin-mesocarp - Piaçava-kernel | 2.447151 | | | 1.44E-02 | | 1 |  |
| 104 | Insects - Piaçava-kernel | 0.597093 | | | 5.50E-01 | | 1 |  |
| 105 | Piaçava-exocarp - Piaçava-kernel | 2.687996 | | | 7.19E-03 | | 1 |  |
| 106 | Bananinha- Piaçava-mesocarp | 1.493377 | | | 1.35E-01 | | 1 |  |
| 107 | Berry - Piaçava-mesocarp | 1.538517 | | | 1.24E-01 | | 1 |  |
| 108 | Bromeliad leaf - Piaçava-mesocarp | -0.21422 | | | 8.30E-01 | | 1 |  |
| 109 | Cane - Piaçava-mesocarp | 4.210817 | | | 2.54E-05 | | **0.01** |  |
| 110 | Fruta podoin-seed - Piaçava-mesocarp | 0.780801 | | | 4.35E-01 | | 1 |  |
| 111 | Fruta d'anta-endocarp - Piaçava-mesocarp | -0.89585 | | | 3.70E-01 | | 1 |  |
| 112 | Fruta d'anta-exocarp - Piaçava-mesocarp | -0.53216 | | | 5.95E-01 | | 1 |  |
| 113 | Fruta d'anta-mesocarp - Piaçava-mesocarp | -0.21065 | | | 8.33E-01 | | 1 |  |
| 114 | Fruta de Caju-endocarp - Piaçava-mesocarp | 2.145308 | | | 3.19E-02 | | 1 |  |
| 115 | Fruta de Caju-exocarp - Piaçava-mesocarp | 3.722071 | | | 1.98E-04 | | **0.04** |  |
| 116 | Fruta de Caju-mesocarp - Piaçava-mesocarp | 2.251436 | | | 2.44E-02 | | 1 |  |
| 117 | Fruta podoin-mesocarp - Piaçava-mesocarp | 2.3947 | | | 1.66E-02 | | 1 |  |
| 118 | Insects - Piaçava-mesocarp | 0.5079 | | | 6.12E-01 | | 1 |  |
| 119 | Piaçava-exocarp - Piaçava-mesocarp | 2.639908 | | | 8.29E-03 | | 1 |  |
| 120 | Piaçava-kernel - Piaçava-mesocarp | -0.13174 | | | 8.95E-01 | | 1 |  |
| 121 | Bananinha- Pod | 0.290197 | | | 7.72E-01 | | 1 |  |
| 122 | Berry - Pod | 0.51404 | | | 6.07E-01 | | 1 |  |
| 123 | Bromeliad leaf - Pod | -1.30022 | | | 1.94E-01 | | 1 |  |
| 124 | Cane - Pod | 2.690192 | | | 7.14E-03 | | 1 |  |
| 125 | Fruta podoin-seed - Pod | -0.45716 | | | 6.48E-01 | | 1 |  |
| 126 | Fruta d'anta-endocarp - Pod | -2.00576 | | | 4.49E-02 | | 1 |  |
| 127 | Fruta d'anta-exocarp - Pod | -2.22219 | | | 2.63E-02 | | 1 |  |
| 128 | Fruta d'anta-mesocarp - Pod | -1.77307 | | | 7.62E-02 | | 1 |  |
| 129 | Fruta de Caju-endocarp - Pod | 0.973948 | | | 3.30E-01 | | 1 |  |
| 130 | Fruta de Caju-exocarp - Pod | 2.627671 | | | 8.60E-03 | | 1 |  |
| 131 | Fruta de Caju-mesocarp - Pod | 1.085256 | | | 2.78E-01 | | 1 |  |
| 132 | Fruta podoin-mesocarp - Pod | 0.939172 | | | 3.48E-01 | | 1 |  |
| 133 | Insects - Pod | -0.74338 | | | 4.57E-01 | | 1 |  |
| 134 | Piaçava-exocarp - Pod | 1.373356 | | | 1.70E-01 | | 1 |  |
| 135 | Piaçava-kernel - Pod | -1.8753 | | | 6.08E-02 | | 1 |  |
| 136 | Piaçava-mesocarp - Pod | -1.80464 | | | 7.11E-02 | | 1 |  |
| 137 | Bananinha- USO | -1.32224 | | | 1.86E-01 | | 1 |  |
| 138 | Berry - USO | -0.85866 | | | 3.91E-01 | | 1 |  |
| 139 | Bromeliad leaf - USO | -2.64755 | | | 8.11E-03 | | 1 |  |
| 140 | Cane - USO | 0.094689 | | | 9.25E-01 | | 1 |  |
| 141 | Fruta podoin-seed - USO | -2.05508 | | | 3.99E-02 | | 1 |  |
| 142 | Fruta d'anta-endocarp - USO | -3.34322 | | | 8.28E-04 | | 0.17 |  |
| 143 | Fruta d'anta-exocarp - USO | -4.1999 | | | 2.67E-05 | | **0.01** |  |
| 144 | Fruta d'anta-mesocarp - USO | -3.65425 | | | 2.58E-04 | | **0.05** |  |
| 145 | Fruta de Caju-endocarp - USO | -0.65176 | | | 5.15E-01 | | 1 |  |
| 146 | Fruta de Caju-exocarp - USO | 0.969847 | | | 3.32E-01 | | 1 |  |
| 147 | Fruta de Caju-mesocarp - USO | -0.54262 | | | 5.87E-01 | | 1 |  |
| 148 | Fruta podoin-mesocarp - USO | -1.13892 | | | 2.55E-01 | | 1 |  |
| 149 | Insects - USO | -2.33574 | | | 1.95E-02 | | 1 |  |
| 150 | Piaçava-exocarp - USO | -0.44762 | | | 6.54E-01 | | 1 |  |
| 151 | Piaçava-kernel - USO | -3.95614 | | | 7.62E-05 | | **0.02** |  |
| 152 | Piaçava-mesocarp - USO | -3.96639 | | | 7.30E-05 | | **0.02** |  |
| 153 | Pod - USO | -2.47031 | | | 1.35E-02 | | 1 |  |
| 154 | Bananinha- Tucum-exocarp | -0.07984 | | | 9.36E-01 | | 1 |  |
| 155 | Berry - Tucum-exocarp | 0.182279 | | | 8.55E-01 | | 1 |  |
| 156 | Bromeliad leaf - Tucum-exocarp | -1.5148 | | | 1.30E-01 | | 1 |  |
| 157 | Cane - Tucum-exocarp | 1.708985 | | | 8.75E-02 | | 1 |  |
| 158 | Fruta podoin-seed - Tucum-exocarp | -0.76208 | | | 4.46E-01 | | 1 |  |
| 159 | Fruta d'anta-endocarp - Tucum-exocarp | -2.17478 | | | 2.96E-02 | | 1 |  |
| 160 | Fruta d'anta-exocarp - Tucum-exocarp | -2.3467 | | | 1.89E-02 | | 1 |  |
| 161 | Fruta d'anta-mesocarp - Tucum-exocarp | -1.9612 | | | 4.99E-02 | | 1 |  |
| 162 | Fruta de Caju-endocarp - Tucum-exocarp | 0.54434 | | | 5.86E-01 | | 1 |  |
| 163 | Fruta de Caju-exocarp - Tucum-exocarp | 2.053976 | | | 4.00E-02 | | 1 |  |
| 164 | Fruta de Caju-mesocarp - Tucum-exocarp | 0.64595 | | | 5.18E-01 | | 1 |  |
| 165 | Fruta podoin-mesocarp - Tucum-exocarp | 0.391117 | | | 6.96E-01 | | 1 |  |
| 166 | Insects - Tucum-exocarp | -1.02336 | | | 3.06E-01 | | 1 |  |
| 167 | Piaçava-exocarp - Tucum-exocarp | 0.850713 | | | 3.95E-01 | | 1 |  |
| 168 | Piaçava-kernel - Tucum-exocarp | -2.04127 | | | 4.12E-02 | | 1 |  |
| 169 | Piaçava-mesocarp - Tucum-exocarp | -1.97658 | | | 4.81E-02 | | 1 |  |
| 170 | Pod - Tucum-exocarp | -0.48755 | | | 6.26E-01 | | 1 |  |
| 171 | USO - Tucum-exocarp | 1.576205 | | | 1.15E-01 | | 1 |  |
| 172 | Bananinha- Tucum-kernel | -0.80562 | | | 4.20E-01 | | 1 |  |
| 173 | Berry - Tucum-kernel | -0.44627 | | | 6.55E-01 | | 1 |  |
| 174 | Bromeliad leaf - Tucum-kernel | -2.14335 | | | 3.21E-02 | | 1 |  |
| 175 | Cane - Tucum-kernel | 0.682567 | | | 4.95E-01 | | 1 |  |
| 176 | Fruta podoin-seed - Tucum-kernel | -1.48786 | | | 1.37E-01 | | 1 |  |
| 177 | Fruta d'anta-endocarp - Tucum-kernel | -2.80333 | | | 5.06E-03 | | 1 |  |
| 178 | Fruta d'anta-exocarp - Tucum-kernel | -3.23561 | | | 1.21E-03 | | 0.25 |  |
| 179 | Fruta d'anta-mesocarp - Tucum-kernel | -2.80873 | | | 4.97E-03 | | 1 |  |
| 180 | Fruta de Caju-endocarp - Tucum-kernel | -0.18145 | | | 8.56E-01 | | 1 |  |
| 181 | Fruta de Caju-exocarp - Tucum-kernel | 1.328189 | | | 1.84E-01 | | 1 |  |
| 182 | Fruta de Caju-mesocarp - Tucum-kernel | -0.07984 | | | 9.36E-01 | | 1 |  |
| 183 | Fruta podoin-mesocarp - Tucum-kernel | -0.49779 | | | 6.19E-01 | | 1 |  |
| 184 | Insects - Tucum-kernel | -1.74915 | | | 8.03E-02 | | 1 |  |
| 185 | Piaçava-exocarp - Tucum-kernel | 0.055654 | | | 9.56E-01 | | 1 |  |
| 186 | Piaçava-kernel - Tucum-kernel | -2.96373 | | | 3.04E-03 | | 0.64 |  |
| 187 | Piaçava-mesocarp - Tucum-kernel | -2.92686 | | | 3.42E-03 | | 0.72 |  |
| 188 | Pod - Tucum-kernel | -1.51397 | | | 1.30E-01 | | 1 |  |
| 189 | USO - Tucum-kernel | 0.582381 | | | 5.60E-01 | | 1 |  |
| 190 | Tucum-exocarp - Tucum-kernel | -0.8889 | | | 3.74E-01 | | 1 |  |
| 191 | Bananinha- Tucum-mesocarp | -0.56611 | | | 5.71E-01 | | 1 |  |
| 192 | Berry - Tucum-mesocarp | -0.23885 | | | 8.11E-01 | | 1 |  |
| 193 | Bromeliad leaf - Tucum-mesocarp | -1.93593 | | | 5.29E-02 | | 1 |  |
| 194 | Cane - Tucum-mesocarp | 1.021285 | | | 3.07E-01 | | 1 |  |
| 195 | Fruta podoin-seed - Tucum-mesocarp | -1.24835 | | | 2.12E-01 | | 1 |  |
| 196 | Fruta d'anta-endocarp - Tucum-mesocarp | -2.59591 | | | 9.43E-03 | | 1 |  |
| 197 | Fruta d'anta-exocarp - Tucum-mesocarp | -2.94227 | | | 3.26E-03 | | 0.68 |  |
| 198 | Fruta d'anta-mesocarp - Tucum-mesocarp | -2.52905 | | | 1.14E-02 | | 1 |  |
| 199 | Fruta de Caju-endocarp - Tucum-mesocarp | 0.058063 | | | 9.54E-01 | | 1 |  |
| 200 | Fruta de Caju-exocarp - Tucum-mesocarp | 1.567699 | | | 1.17E-01 | | 1 |  |
| 201 | Fruta de Caju-mesocarp - Tucum-mesocarp | 0.159673 | | | 8.73E-01 | | 1 |  |
| 202 | Fruta podoin-mesocarp - Tucum-mesocarp | -0.20445 | | | 8.38E-01 | | 1 |  |
| 203 | Insects - Tucum-mesocarp | -1.50964 | | | 1.31E-01 | | 1 |  |
| 204 | Piaçava-exocarp - Tucum-mesocarp | 0.318024 | | | 7.50E-01 | | 1 |  |
| 205 | Piaçava-kernel - Tucum-mesocarp | -2.65932 | | | 7.83E-03 | | 1 |  |
| 206 | Piaçava-mesocarp - Tucum-mesocarp | -2.61326 | | | 8.97E-03 | | 1 |  |
| 207 | Pod - Tucum-mesocarp | -1.17525 | | | 2.40E-01 | | 1 |  |
| 208 | USO - Tucum-mesocarp | 0.910343 | | | 3.63E-01 | | 1 |  |
| 209 | Tucum-exocarp - Tucum-mesocarp | -0.59557 | | | 5.51E-01 | | 1 |  |
| 210 | Tucum-kernel - Tucum-mesocarp | 0.293338 | | | 7.69E-01 | | 1 |  |

**SOM Table S5**

Results of a Dunn test of multiple comparisons with a Bonferroni correction testing for differences in elastic modulus values between food items. Significant values are bolded. In pairwise comparisons of elastic modulus values (without ‘piaçava’endocarp), ‘piaçava’ exocarp had significantly higher elastic modulus values than all other foods (all *p* < 0.01). Without ‘piaçava’endocarp, elastic modulus values from ‘piaçava’ mesocarp were significantly higher than all other foods except tucum mesocarp (*p* = 0.08) and ‘fruta podoin’ (*Copaifera* sp.) endocarp (*p* = 0.60), and elastic modulus values from tucum kernel were significantly higher than all foods except tucum mesocarp (*p* = 0.20), ‘fruta podoin’ endocarp (*p* = 0.67), and ‘piaçava’ mesocarp (*p* = 0.99).

| Test number | Comparison | Z | Unadjusted *p* | Adjusted *p* |
| --- | --- | --- | --- | --- |
| 1 | Bananinha- Berry | 0.20087 | 8.41E-01 | 1 |
| 2 | Bananinha- Insects | -2.69221 | 7.10E-03 | 0.55 |
| 3 | Berry - Insects | -2.84647 | 4.42E-03 | 0.34 |
| 4 | Bananinha- Cane | -2.31461 | 2.06E-02 | 1 |
| 5 | Berry - Cane | -2.55777 | 1.05E-02 | 0.82 |
| 6 | Insects - Cane | 1.127511 | 2.60E-01 | 1 |
| 7 | Bananinha- Fruta d'anta-endocarp | -2.04551 | 4.08E-02 | 1 |
| 8 | Berry - Fruta d'anta-endocarp | -2.19392 | 2.82E-02 | 1 |
| 9 | Insects - Fruta d'anta-endocarp | 0.515887 | 6.06E-01 | 1 |
| 10 | Cane - Fruta d'anta-endocarp | -0.48763 | 6.26E-01 | 1 |
| 11 | Bananinha- Fruta d'anta-mesocarp | -1.88941 | 5.88E-02 | 1 |
| 12 | Berry - Fruta d'anta-mesocarp | -2.03641 | 4.17E-02 | 1 |
| 13 | Insects - Fruta d'anta-mesocarp | 0.640411 | 5.22E-01 | 1 |
| 14 | Cane - Fruta d'anta-mesocarp | -0.33318 | 7.39E-01 | 1 |
| 15 | Fruta d'anta-endocarp - Fruta d'anta-mesocarp | 0.124524 | 9.01E-01 | 1 |
| 16 | Bananinha- Fruta Podoin-endocarp | -1.95702 | 5.03E-02 | 1 |
| 17 | Berry - Fruta Podoin-endocarp | -2.04441 | 4.09E-02 | 1 |
| 18 | Insects - Fruta Podoin-endocarp | -0.25158 | 8.01E-01 | 1 |
| 19 | Cane - Fruta Podoin-endocarp | -0.98465 | 3.25E-01 | 1 |
| 20 | Fruta d'anta-endocarp - Fruta Podoin-endocarp | -0.61636 | 5.38E-01 | 1 |
| 21 | Fruta d'anta-mesocarp - Fruta Podoin-endocarp | -0.70442 | 4.81E-01 | 1 |
| 22 | Bananinha- Piaçava-exocarp | -4.65758 | 3.20E-06 | **<0.01** |
| 23 | Berry - Piaçava-exocarp | -4.85544 | 1.20E-06 | **<0.01** |
| 24 | Insects - Piaçava-exocarp | -1.26466 | 2.06E-01 | 1 |
| 25 | Cane - Piaçava-exocarp | -2.88725 | 3.89E-03 | 0.30 |
| 26 | Fruta d'anta-endocarp - Piaçava-exocarp | -1.81617 | 6.93E-02 | 1 |
| 27 | Fruta d'anta-mesocarp - Piaçava-exocarp | -1.94929 | 5.13E-02 | 1 |
| 28 | Fruta Podoin-endocarp - Piaçava-exocarp | -0.6041 | 5.46E-01 | 1 |
| 29 | Bananinha- Piaçava-kernel | -3.09496 | 1.97E-03 | 0.15 |
| 30 | Berry - Piaçava-kernel | -3.29359 | 9.89E-04 | 0.08 |
| 31 | Insects - Piaçava-kernel | 0.115356 | 9.08E-01 | 1 |
| 32 | Cane - Piaçava-kernel | -1.20129 | 2.30E-01 | 1 |
| 33 | Fruta d'anta-endocarp - Piaçava-kernel | -0.46142 | 6.44E-01 | 1 |
| 34 | Fruta d'anta-mesocarp - Piaçava-kernel | -0.60065 | 5.48E-01 | 1 |
| 35 | Fruta Podoin-endocarp - Piaçava-kernel | 0.34209 | 7.32E-01 | 1 |
| 36 | Piaçava-exocarp - Piaçava-kernel | 1.565463 | 1.17E-01 | 1 |
| 37 | Bananinha- Piaçava-mesocarp | -5.40619 | 6.44E-08 | **<0.01** |
| 38 | Berry - Piaçava-mesocarp | -5.71258 | 1.11E-08 | **<0.01** |
| 39 | Insects - Piaçava-mesocarp | -0.92451 | 3.55E-01 | 1 |
| 40 | Cane - Piaçava-mesocarp | -3.0205 | 2.52E-03 | 0.20 |
| 41 | Fruta d'anta-endocarp - Piaçava-mesocarp | -1.56439 | 1.18E-01 | 1 |
| 42 | Fruta d'anta-mesocarp - Piaçava-mesocarp | -1.71885 | 8.56E-02 | 1 |
| 43 | Fruta Podoin-endocarp - Piaçava-mesocarp | -0.30329 | 7.62E-01 | 1 |
| 44 | Piaçava-exocarp - Piaçava-mesocarp | 0.603965 | 5.46E-01 | 1 |
| 45 | Piaçava-kernel - Piaçava-mesocarp | -1.26494 | 2.06E-01 | 1 |
| 46 | Bananinha- Pod | -1.49191 | 1.36E-01 | 1 |
| 47 | Berry - Pod | -1.70249 | 8.87E-02 | 1 |
| 48 | Insects - Pod | 1.566185 | 1.17E-01 | 1 |
| 49 | Cane - Pod | 0.6706 | 5.02E-01 | 1 |
| 50 | Fruta d'anta-endocarp - Pod | 0.944002 | 3.45E-01 | 1 |
| 51 | Fruta d'anta-mesocarp - Pod | 0.79382 | 4.27E-01 | 1 |
| 52 | Fruta Podoin-endocarp - Pod | 1.273554 | 2.03E-01 | 1 |
| 53 | Piaçava-exocarp - Pod | 3.308792 | 9.37E-04 | 0.07 |
| 54 | Piaçava-kernel - Pod | 1.712137 | 8.69E-02 | 1 |
| 55 | Piaçava-mesocarp - Pod | 3.518355 | 4.34E-04 | **0.03** |
| 56 | Bananinha- Tucum-kernel | -4.41991 | 9.87E-06 | **<0.01** |
| 57 | Berry - Tucum-kernel | -4.63613 | 3.55E-06 | **<0.01** |
| 58 | Insects - Tucum-kernel | -0.86318 | 3.88E-01 | 1 |
| 59 | Cane - Tucum-kernel | -2.50601 | 1.22E-02 | 0.95 |
| 60 | Fruta d'anta-endocarp - Tucum-kernel | -1.43996 | 1.50E-01 | 1 |
| 61 | Fruta d'anta-mesocarp - Tucum-kernel | -1.57918 | 1.14E-01 | 1 |
| 62 | Fruta Podoin-endocarp - Tucum-kernel | -0.31027 | 7.56E-01 | 1 |
| 63 | Piaçava-exocarp - Tucum-kernel | 0.500169 | 6.17E-01 | 1 |
| 64 | Piaçava-kernel - Tucum-kernel | -1.12992 | 2.59E-01 | 1 |
| 65 | Piaçava-mesocarp - Tucum-kernel | -0.03978 | 9.68E-01 | 1 |
| 66 | Pod - Tucum-kernel | -2.96567 | 3.02E-03 | 0.24 |
| 67 | Bananinha- Tucum-mesocarp | -3.51948 | 4.32E-04 | **0.03** |
| 68 | Berry - Tucum-mesocarp | -3.70448 | 2.12E-04 | **0.02** |
| 69 | Insects - Tucum-mesocarp | -0.39461 | 6.93E-01 | 1 |
| 70 | Cane - Tucum-mesocarp | -1.76402 | 7.77E-02 | 1 |
| 71 | Fruta d'anta-endocarp - Tucum-mesocarp | -0.94612 | 3.44E-01 | 1 |
| 72 | Fruta d'anta-mesocarp - Tucum-mesocarp | -1.07924 | 2.80E-01 | 1 |
| 73 | Fruta Podoin-endocarp - Tucum-mesocarp | -0.00974 | 9.92E-01 | 1 |
| 74 | Piaçava-exocarp - Tucum-mesocarp | 0.93976 | 3.47E-01 | 1 |
| 75 | Piaçava-kernel - Tucum-mesocarp | -0.57487 | 5.65E-01 | 1 |
| 76 | Piaçava-mesocarp - Tucum-mesocarp | 0.519263 | 6.04E-01 | 1 |
| 77 | Pod - Tucum-mesocarp | -2.22365 | 2.62E-02 | 1 |
| 78 | Tucum-kernel - Tucum-mesocarp | 0.490425 | 6.24E-01 | 1 |

**SOM Table S6**

Results from the LME models for each hypothesis. Abbreviations: USO = underground storage organs.

| **Hypothesis 1-Feeding sequence-manual behaviors, ingestive behaviors, and mastication** | | | | | | | |  | |
| --- | --- | --- | --- | --- | --- | --- | --- | --- | --- |
| **Toughness-manual vs ingestive behaviors** | **Estimate** | **Error** | **df** | | **t-value** | | **Pr(>\|t\|)** | |  |
| (Intercept) | 2267.88 | 593.18 | 9.03 | | 3.82 | | <0.01 | |  |
| Manual behaviors | 1969.92 | 139.69 | 1409.87 | | 14.10 | | <0.01 | |  |
| **Post-hoc Tukey comparison** | **Estimate** | **Error** | **z-value** | | **Pr(>\|z\|)** | |  | |  |
| Manual-Ingestive | 1969.90 | 139.70 | 14.10 | | <0.01 | |  | |  |
| **Elastic modulus-manual vs ingestive behaviors** | **Estimate** | **Error** | **df** | | **t-value** | | **Pr(>\|t\|)** | |  |
| (Intercept) | 10.85 | 12.56 | 6.77 | | 0.86 | | 0.42 | |  |
| Manual behaviors | 46.30 | 1.67 | 990.77 | | 27.81 | | <0.01 | |  |
| **Post-hoc Tukey comparison** | **Estimate** | **Error** | **z-value** | | **Pr(>\|z\|)** | |  | |  |
| Manual-Ingestive | 46.30 | 1.67 | 27.81 | | <0.01 | |  | |  |
| **Duration-mastication, manual, vs ingestive behaviors** | **Estimate** | **Error** | **df** | | **t-value** | | **Pr(>\|t\|)** | |  |
| (Intercept) | 1.37 | 0.15 | 7.37 | | 8.91 | | <0.01 | |  |
| Manual behaviors | 2.15 | 0.31 | 1312.00 | | 7.01 | | <0.01 | |  |
| Mastication | 1.95 | 0.13 | 1416.52 | | 14.54 | | <0.01 | |  |
| **Post-hoc Tukey comparison** | **Estimate** | **Error** | **z-value** | | **Pr(>\|z\|)** | |  | |  |
| Manual - Ingestive | 2.15 | 0.31 | 7.01 | | <0.01 | |  | |  |
| Masticate - Ingestive | 1.95 | 0.13 | 14.54 | | <0.01 | |  | |  |
| Mastication - Manual | -0.20 | 0.32 | -0.63 | | 0.80 | |  | |  |
| **Hypothesis 2-Oral and oral-manual ingestive behaviors** | | | |  | |  | |  | |
| **Toughness-oral vs oral-manual ingestive behaviors** | **Estimate** | **Error** | **df** | | **t-value** | | **Pr(>\|t\|)** | |  |
| (Intercept) | 2445.02 | 669.26 | 9.06 | | 3.65 | | <0.01 | |  |
| Oral-Manual | -129.90 | 42.05 | 997.22 | | -3.09 | | <0.01 | |  |
| **Post-hoc Tukey comparison** | **Estimate** | **Error** | **z-value** | | **Pr(>\|z\|)** | |  | |  |
| Oral-Manual-oral | -129.90 | 42.05 | -3.09 | | <0.01 | |  | |  |
| **Elastic modulus-oral vs oral-manual ingestive behaviors** | **Estimate** | **Error** | **df** | | **t-value** | | **Pr(>\|t\|)** | |  |
| (Intercept) | 15.09 | 9.38 | 6.00 | | 1.61 | | 0.16 | |  |
| Oral-Manual | -0.06 | 0.04 | 702.00 | | -1.61 | | 0.11 | |  |
| **Post-hoc Tukey comparison** | **Estimate** | **Error** | **z-value** | | **Pr(>\|z\|)** | |  | |  |
| Oral-Manual-oral | -0.06 | 0.04 | -1.61 | | 0.11 | |  | |  |
| **Duration-oral vs oral-manual ingestive behaviors** | **Estimate** | **Error** | **df** | | **t-value** | | **Pr(>\|t\|)** | |  |
| (Intercept) | 0.93 | 0.25 | 85.31 | | 3.72 | | <0.01 | |  |
| Oral-Manual | 0.47 | 0.26 | 231.42 | | 1.84 | | 0.07 | |  |
| **Post-hoc Tukey comparison** | **Estimate** | **Error** | **z-value** | | **Pr(>\|z\|)** | |  | |  |
| Oral-Manual-oral | 0.47 | 0.26 | 1.84 | | 0.07 | |  | |  |
| **Hypothesis 3-Toothrow positioning** |  |  |  | |  | |  | |  |
| **Toughness-anterior vs posterior ingestive behaviors** | **Estimate** | **Error** | **df** | | **t-value** | | **Pr(>\|t\|)** | |  |
| (Intercept) | 2902.06 | 508.65 | 8.95 | | 5.71 | | <0.01 | |  |
| Oral-Manual ingestive behaviors-Posterior dentition | -111.21 | 34.14 | 973.99 | | -3.26 | | <0.01 | |  |
| Oral ingestive behaviors-Anterior dentition | 201.51 | 55.30 | 975.91 | | 3.64 | | <0.01 | |  |
| **Post-hoc Tukey comparison** | **Estimate** | **Error** | **z-value** | | **Pr(>\|z\|)** | |  | |  |
| Oral-Manual Posterior-Oral-Manual Anterior | -111.21 | 34.14 | -3.26 | | <0.01 | |  | |  |
| Oral Anterior-Oral-Manual Anterior | 201.51 | 55.30 | 3.64 | | <0.01 | |  | |  |
| Oral Anterior-Oral-Manual Posterior | 312.71 | 52.16 | 6.00 | | <0.01 | |  | |  |
| **Elastic modulus-anterior vs posterior ingestive behaviors** | **Estimate** | **Error** | **df** | | **t-value** | | **Pr(>\|t\|)** | |  |
| (Intercept) | 13.85 | 5.03 | 12.54 | | 2.75 | | 0.02 | |  |
| Oral-Manual ingestive behaviors-Posterior dentition | -0.24 | 0.03 | 685.01 | | -6.97 | | <0.01 | |  |
| Oral ingestive behaviors-Anterior dentition | -0.05 | 0.05 | 685.01 | | -1.03 | | 0.30 | |  |
| **Post-hoc Tukey comparison** | **Estimate** | **Error** | **z-value** | | **Pr(>\|z\|)** | |  | |  |
| Oral-Manual Posterior-Oral-Manual Anterior | -0.24 | 0.03 | -6.97 | | <0.01 | |  | |  |
| Oral Anterior-Oral-Manual Anterior | -0.05 | 0.05 | -1.03 | | 0.55 | |  | |  |
| Oral Anterior-Oral-Manual Posterior | 0.19 | 0.04 | 4.24 | | <0.01 | |  | |  |
| **Duration-anterior vs posterior ingestive behaviors** | **Estimate** | **Error** | **df** | | **t-value** | | **Pr(>\|t\|)** | |  |
| (Intercept) | 1.11 | 0.09 | 13.34 | | 12.75 | | <0.01 | |  |
| Oral-Manual ingestive behaviors-Posterior dentition | 0.72 | 0.11 | 515.84 | | 6.52 | | <0.01 | |  |
| Oral ingestive behaviors-Anterior dentition | -0.19 | 0.15 | 349.52 | | -1.29 | | 0.20 | |  |
| **Post-hoc Tukey comparison** | **Estimate** | **Error** | **z-value** | | **Pr(>\|z\|)** | |  | |  |
| Oral-Manual Posterior-Oral-Manual Anterior | 0.72 | 0.11 | 6.52 | | <0.01 | |  | |  |
| Oral Anterior-Oral-Manual Anterior | -0.19 | 0.15 | -1.29 | | 0.40 | |  | |  |
| Oral Anterior-Oral-Manual Posterior | -0.91 | 0.15 | -5.96 | | <0.01 | |  | |  |
| **Hypothesis 1-Within foods-Feeding sequence-manual behaviors, ingestive behaviors, and mastication** | | | | | | | | | |
| **Fruit-Duration-mastication, manual, vs ingestive behaviors** | **Estimate** | **Error** | **df** | | **t-value** | | **Pr(>\|t\|)** | |  |
| (Intercept) | 1.05 | 0.17 | 6.37 | | 6.11 | | <0.01 | |  |
| Manual behaviors | 0.74 | 0.26 | 334.89 | | 2.85 | | <0.01 | |  |
| Mastication | 1.09 | 0.13 | 335.60 | | 8.65 | | <0.01 | |  |
| **Post-hoc Tukey comparison** | **Estimate** | **Error** | **z-value** | | **Pr(>\|z\|)** | |  | |  |
| Manual - Ingestive | 0.74 | 0.26 | 2.85 | | 0.01 | |  | |  |
| Masticate - Ingestive | 1.09 | 0.13 | 8.65 | | <0.01 | |  | |  |
| Mastication - Manual | 0.35 | 0.27 | 1.28 | | 0.39 | |  | |  |
| **Insects-Duration-mastication, manual, vs ingestive behaviors** | **Estimate** | **Error** | **df** | | **t-value** | | **Pr(>\|t\|)** | |  |
| (Intercept) | 1.10 | 0.22 | 2.55 | | 5.04 | | 0.02 | |  |
| Mastication | 0.65 | 0.29 | 84.68 | | 2.23 | | 0.03 | |  |
| **Post-hoc Tukey comparison** | **Estimate** | **Error** | **z-value** | | **Pr(>\|z\|)** | |  | |  |
| Masticate - Ingestive | 0.65 | 0.29 | 2.23 | | 0.03 | |  | |  |
| **Piaçava-Duration-mastication, manual, vs ingestive behaviors** | **Estimate** | **Error** | **df** | | **t-value** | | **Pr(>\|t\|)** | |  |
| (Intercept) | 1.70 | 0.09 | 235.00 | | 18.67 | | <0.01 | |  |
| Mastication | 1.14 | 0.24 | 235.00 | | 4.65 | | <0.01 | |  |
| **Post-hoc Tukey comparison** | **Estimate** | **Error** | **z-value** | | **Pr(>\|z\|)** | |  | |  |
| Manual - Ingestive | 3.60 | 0.62 | 5.83 | | <0.01 | |  | |  |
| Masticate - Ingestive | 4.28 | 0.66 | 6.49 | | <0.01 | |  | |  |
| Mastication - Manual | 0.67 | 0.78 | 0.86 | | 0.66 | |  | |  |
| **Cane-Duration-mastication, manual, vs ingestive behaviors** | **Estimate** | **Error** | **df** | | **t-value** | | **Pr(>\|t\|)** | |  |
| (Intercept) | 1.70 | 0.09 | 235.00 | | 18.67 | | < 2e-16 | |  |
| Mastication | 1.14 | 0.24 | 235.00 | | 4.65 | | <0.01 | |  |
| **Post-hoc Tukey comparison** | **Estimate** | **Error** | **z-value** | | **Pr(>\|z\|)** | |  | |  |
| Masticate - Ingestive | 1.14 | 0.24 | 4.65 | | <0.01 | |  | |  |
| **Bromeliad-Duration-mastication, manual, vs ingestive behaviors** | **Estimate** | **Error** | **df** | | **t-value** | | **Pr(>\|t\|)** | |  |
| (Intercept) | 1.50 | 0.33 | 1.31 | | 4.57 | | 0.09 | |  |
| Manual behaviors | 0.78 | 1.18 | 43.38 | | 0.66 | | 0.51 | |  |
| Mastication | 0.21 | 0.37 | 43.65 | | 0.57 | | 0.57 | |  |
| **Post-hoc Tukey comparison** | **Estimate** | **Error** | **z-value** | | **Pr(>\|z\|)** | |  | |  |
| Manual - Ingestive | 0.78 | 1.18 | 0.66 | | 0.77 | |  | |  |
| Masticate - Ingestive | 0.21 | 0.37 | 0.57 | | 0.83 | |  | |  |
| Mastication - Manual | -0.57 | 1.21 | -0.47 | | 0.88 | |  | |  |
| **Tucum-Duration-mastication, manual, vs ingestive behaviors** | **Estimate** | **Error** | **df** | | **t-value** | | **Pr(>\|t\|)** | |  |
| (Intercept) | 1.24 | 1.10 | 1.08 | | 1.13 | | 0.45 | |  |
| Manual behaviors | 0.45 | 1.11 | 84.16 | | 0.41 | | 0.68 | |  |
| Mastication | 2.01 | 0.53 | 84.02 | | 3.80 | | <0.01 | |  |
| **Post-hoc Tukey comparison** | **Estimate** | **Error** | **z-value** | | **Pr(>\|z\|)** | |  | |  |
| Manual - Ingestive | 0.45 | 1.11 | 0.41 | | 0.91 | |  | |  |
| Masticate - Ingestive | 2.01 | 0.53 | 3.80 | | <0.01 | |  | |  |
| Mastication - Manual | 1.56 | 1.13 | 1.38 | | 0.34 | |  | |  |
| **Berries-Duration-mastication, manual, vs ingestive behaviors** | **Estimate** | **Error** | **df** | | **t-value** | | **Pr(>\|t\|)** | |  |
| (Intercept) | 1.83 | 0.81 | 145.00 | | 2.27 | | 0.02 | |  |
| Manual behaviors | 5.91 | 4.03 | 145.00 | | 1.47 | | 0.14 | |  |
| Mastication | -0.38 | 1.40 | 145.00 | | -0.27 | | 0.79 | |  |
| **Post-hoc Tukey comparison** | **Estimate** | **Error** | **z-value** | | **Pr(>\|z\|)** | |  | |  |
| Manual - Ingestive | 5.91 | 4.03 | 1.47 | | 0.29 | |  | |  |
| Masticate - Ingestive | -0.38 | 1.40 | -0.27 | | 0.96 | |  | |  |
| Mastication - Manual | -6.29 | 4.11 | -1.53 | | 0.26 | |  | |  |
| **USO-Duration-mastication, manual, vs ingestive behaviors** | **Estimate** | **Error** | **df** | | **t-value** | | **Pr(>\|t\|)** | |  |
| (Intercept) | 1.39 | 0.26 | 2.30 | | 5.32 | | 0.02 | |  |
| Manual behaviors | 2.90 | 1.77 | 321.56 | | 1.64 | | 0.10 | |  |
| Mastication | 2.64 | 0.37 | 322.42 | | 7.14 | | <0.01 | |  |
| **Post-hoc Tukey comparison** | **Estimate** | **Error** | **z-value** | | **Pr(>\|z\|)** | |  | |  |
| Manual - Ingestive | 2.90 | 1.77 | 1.64 | | 0.21 | |  | |  |
| Masticate - Ingestive | 2.64 | 0.37 | 7.14 | | <0.01 | |  | |  |
| Mastication - Manual | -0.26 | 1.79 | -0.15 | | 0.99 | |  | |  |
